# Supplementary material for: Heterologous Hyaluronic Acid Production in Kluyveromyces lactis
Source: Microorganisms. 2019 Aug 28;7(9):294. doi: 10.3390/microorganisms7090294 (PMC6780701; doi:10.3390/microorganisms7090294)

Supplementary Material

Heterologous Hyaluronic Acid production in *Kluyveromyces lactis*

Antônio M. V. Gomes[1], João H. C. M. Netto[1], Lucas S. Carvalho[1] and Nádia S. Parachin[1]*

[1]Grupo de Engenharia de Biocatalisadores, Departamento de Biologia Celular, Instituto de Ciências Biológicas, Universidade de Brasília (UnB), Campus Darcy Ribeiro, Bloco K. 70.790-900. Brasília, Distrito Federal, Brazil.

*** Correspondence:** nadiasp@unb.br;

SUPPORTING INFORMATION CONTENTS

Figure S01. pBSK-hasB---04

Figure S02. p424-GPD-B---05

Figure S03. pBSK-HASAP---06

Figure S04. pBSK-HASA1---07

Figure S05. pBSK-HASA2---08

Figure S06. pBSK-HASA3---09

Figure S07. pKlac2-B---10

Figure S08. pKlac2-BP---11

Figure S09. pKlac2-B1---12

Figure S10. pKlac2-B2---13

Figure S11. pKlac2-B3---14

Sequence S01. pBSK-HASB---15

Sequence S02. p424-GPD-B---16

Sequence S03. pBSK-HASAP---18

Sequence S04. pBSK-HASA1---20

Sequence S05. pBSK-HASA2---22

Sequence S06. pBSK-HASA3---23

Sequence S07. pkLAC2-B---25

Sequence S08. pkLAC2-BP---28

Sequence S09. pkLAC2-B1---33

Sequence S10. pkLAC2-B2--37

Sequence S11. pkLAC2-B3--41

Table S01. Primers utilized in study---45

Figure S12. RNA Integrity Gel--46

Figure S13. RNA Integrity Gel--46

Figure S14. Melt Curve Analysis---47

Figure S15. Melt Curve Analysis---48

Figure S16. Melt Curve Analysis---49

Figure S17. Melt Curve Analysis---50

Figure S18. Melt Curve Analysis---51

Figure S19. Growth profile of the strains--52

Figure S20. Transcript analysis of strains---52

Figure S21. Copy Number Gene---53

Figure S22. GPC analysis of standard HA (Sigma)--54

Figure S23. Standard Curve of Carbazole Method---54

SUPPLEMENTARY INFORMATION

Figure S01. Map of the synthetic plasmid pBSK-HASB containing the *hasB* gene (UDP-Glucose Dehydrogenase) from *Xenopus laevis* (xl*hasB*) optimized for the yeast *K. lactis*. The sites for the *Bam*HI and *Sal*I enzymes are highlighted. The annealing site of the oligonucleotide primers for transcripts analysis (Rt-PCR HASB-F and Rt-PCR HASB-R) and amplification of *hasB* gene (HASB-F and HASB-R) is also shown.

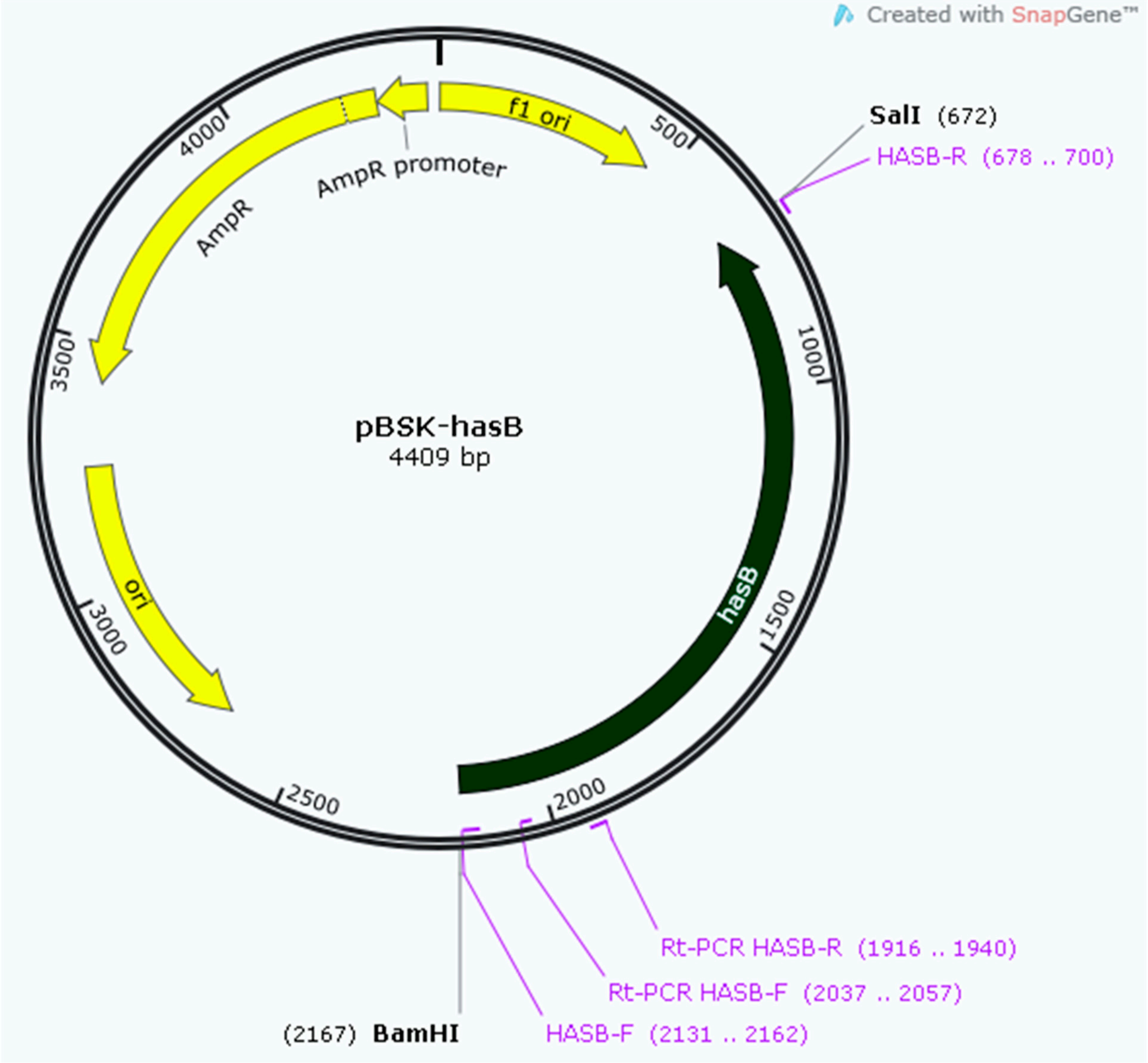

Figure S02. Map of the plasmid p424-GPD-B with xl*hasB* gene (UDP-Glucose Dehydrogenase) cloned between GPD promoter and CYC1 terminator.

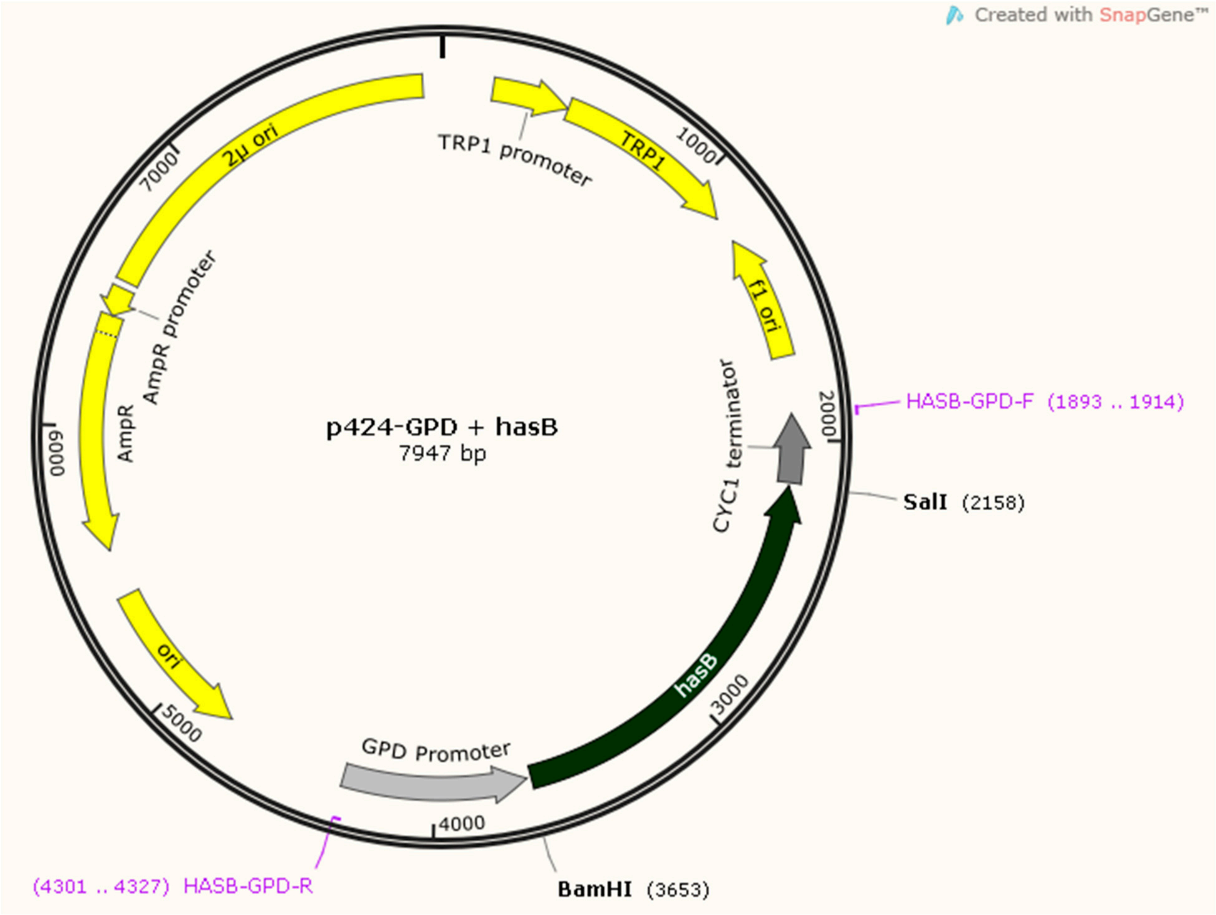

Figure S03. Map of the synthetic plasmid pBSK-HASAP containing the *hasAP* gene (Hyaluronic Acid Synthase) from *Pasteurella multocida* (pm*hasA*) and optimized for the yeast *K. lactis*.

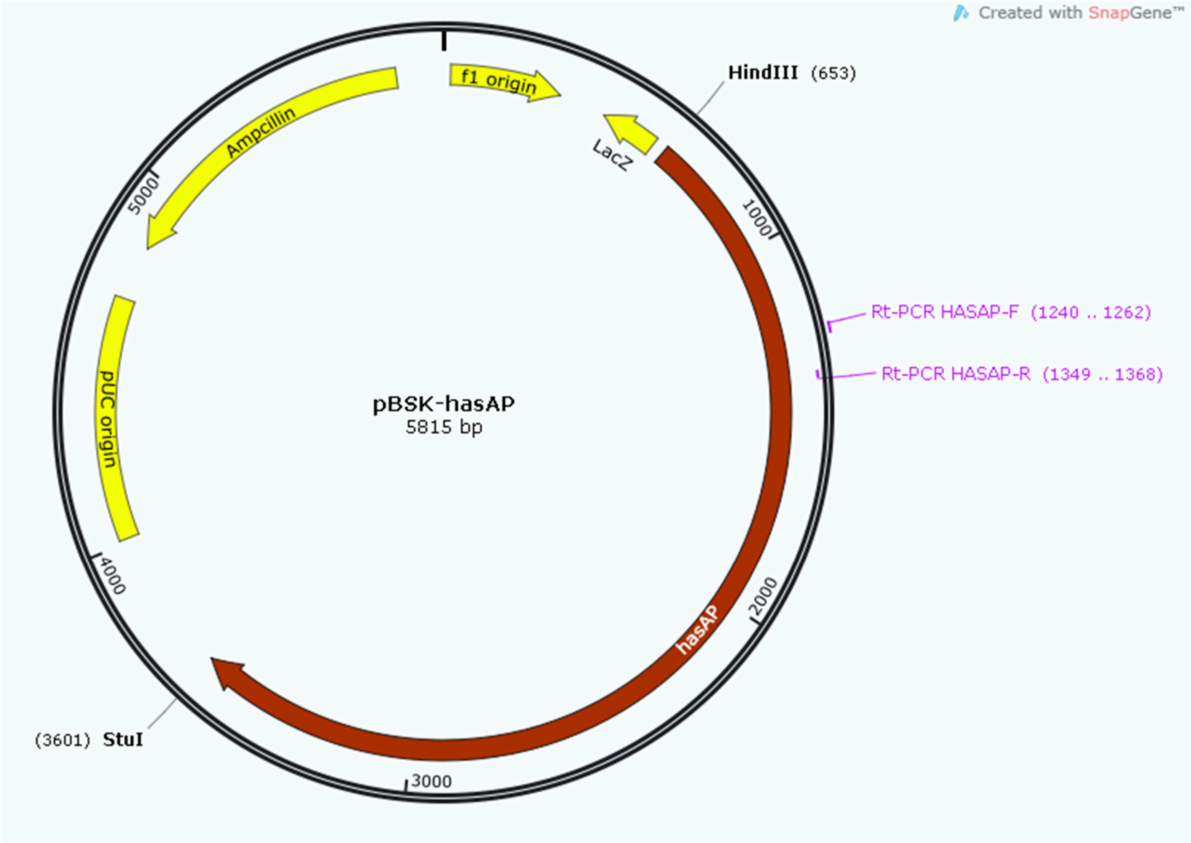

Figure S04. Map of the synthetic plasmid pBSK-HASA1 containing the *hasA1* gene (Hyaluronic Acid Synthase) isoform 1 from *Homo sapiens* (hs*hasA1*) and optimized for the yeast *K. lactis*.

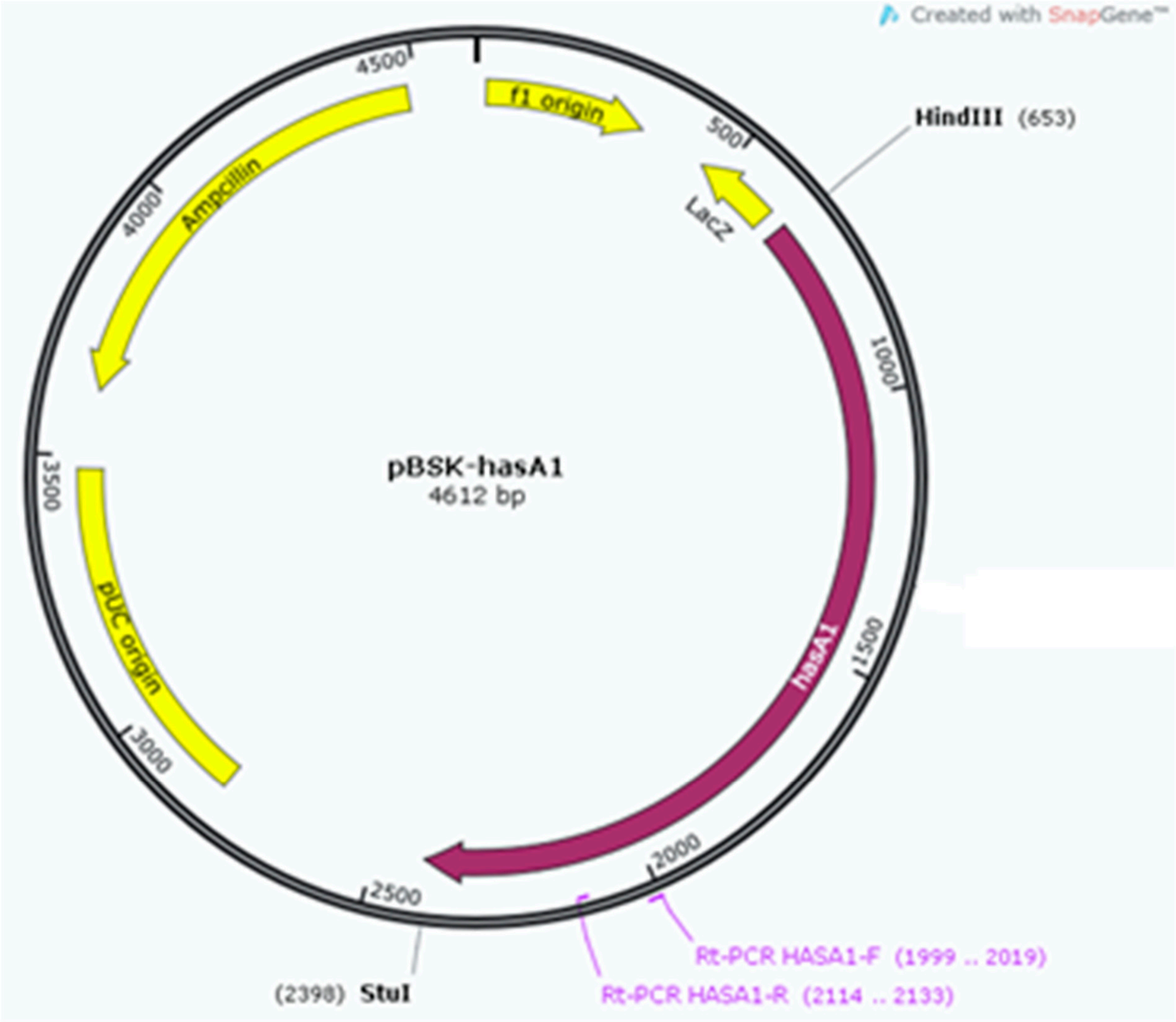

Figure S05. Map of the synthetic plasmid pBSK-HASA2 containing the *hasA2* gene (Hyaluronic Acid Synthase) isoform 2 from *Homo sapiens* (hs*hasA2*) and optimized for the yeast *K. lactis*.

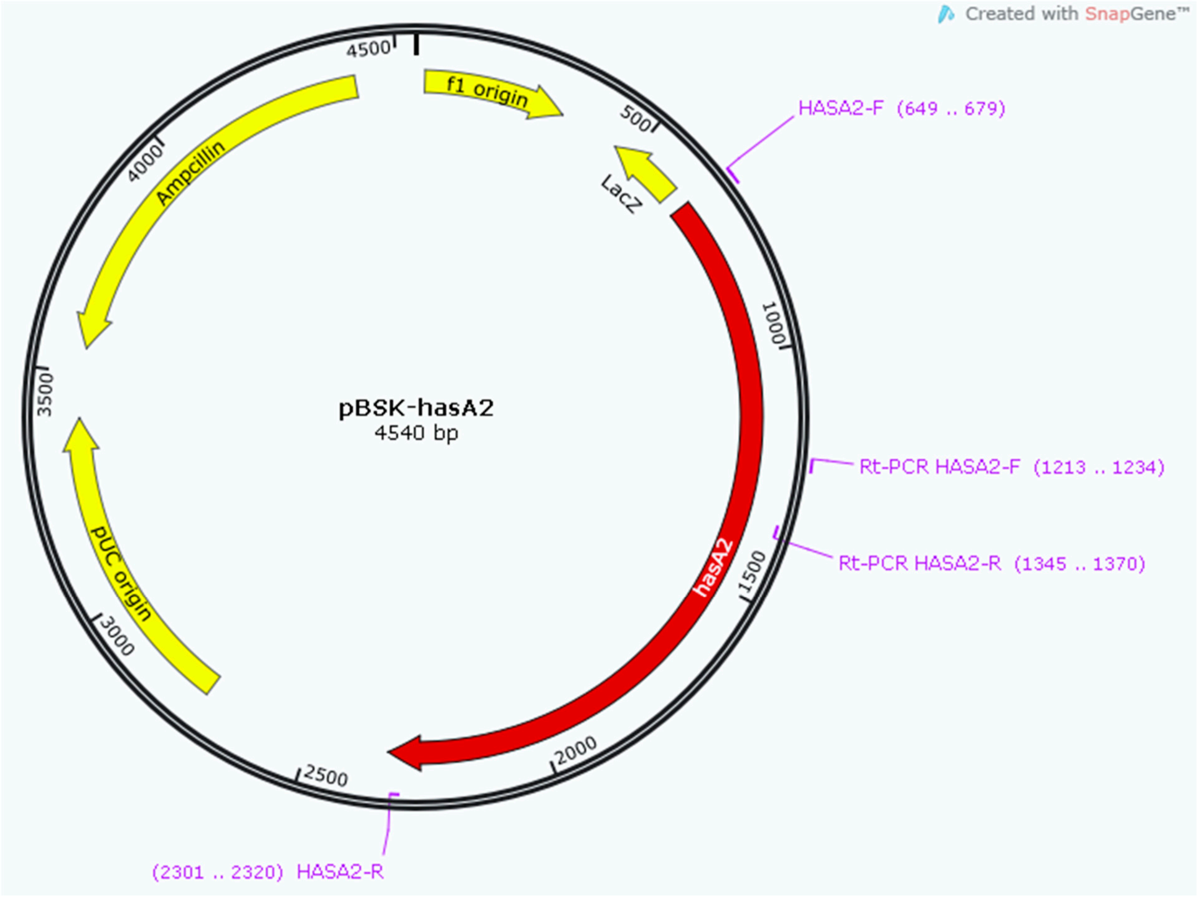

Figure S06. Map of the synthetic plasmid pBSK-HASA3 containing the *hasA3* gene (Hyaluronic Acid Synthase) isoform 3 from *Homo sapiens* (hs*hasA3*) and optimized for the yeast *K. lactis*.

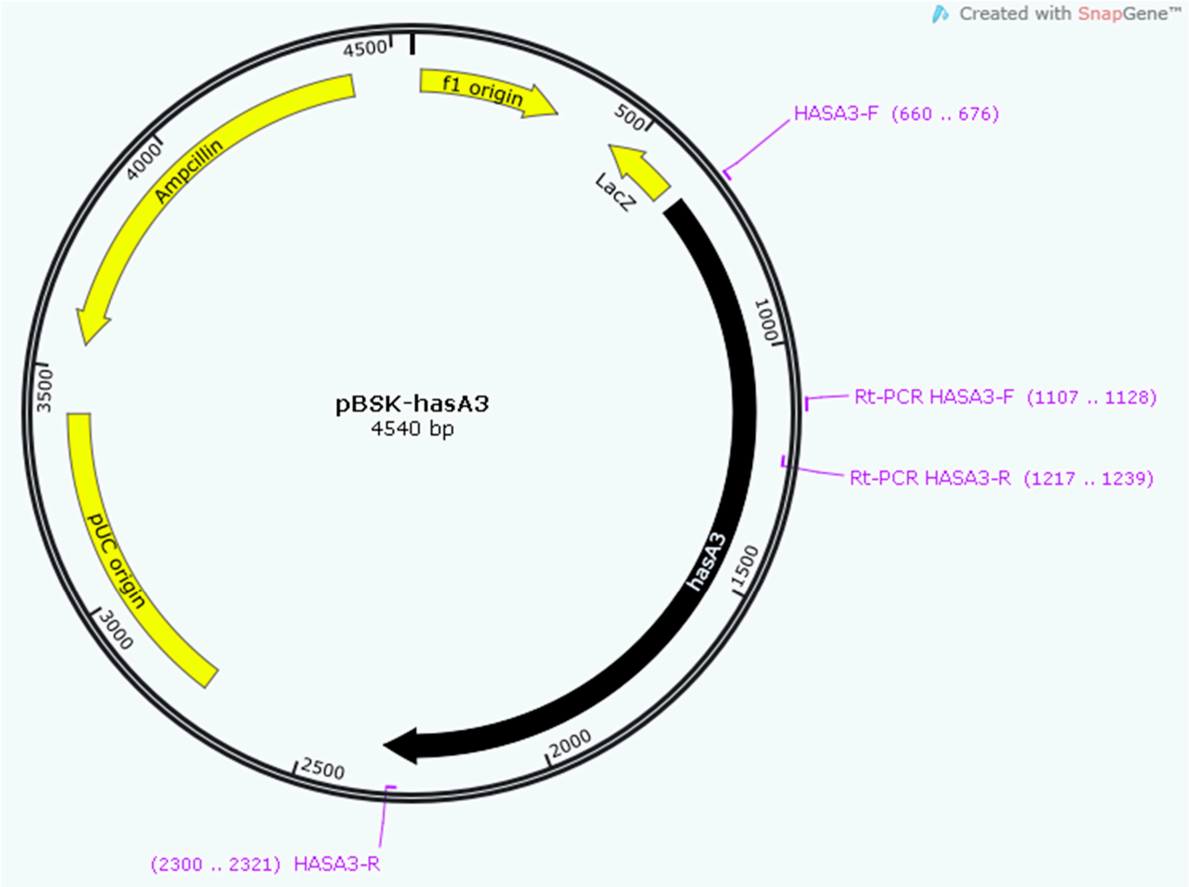

Figure S07. Map of the plasmid pKlac2-B used in this study for integration of the *hasB* gene (UDP-Glucose Dehydrogenase) in genome of *Kluyveromyces lactis*.

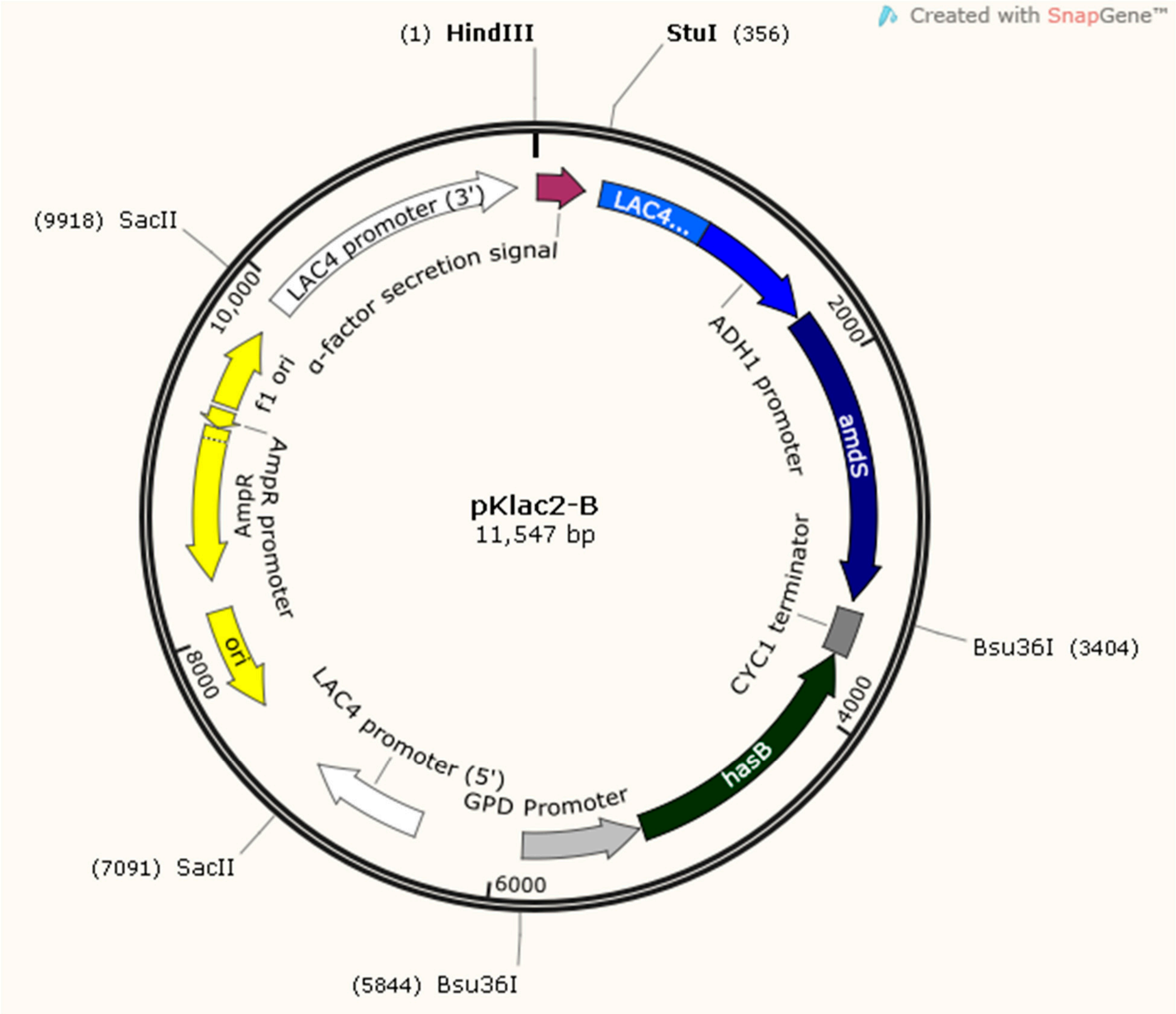

Figure S08. Map of the plasmid pKlac2-BP used in this study for integration of the *hasB* gene (UDP-Glucose Dehydrogenase) from *Xenopus laevis* and *hasAP* gene (Hyaluronic Acid Synthase) from *Pasteurella multocida* in genome of *Kluyveromyces lactis*.

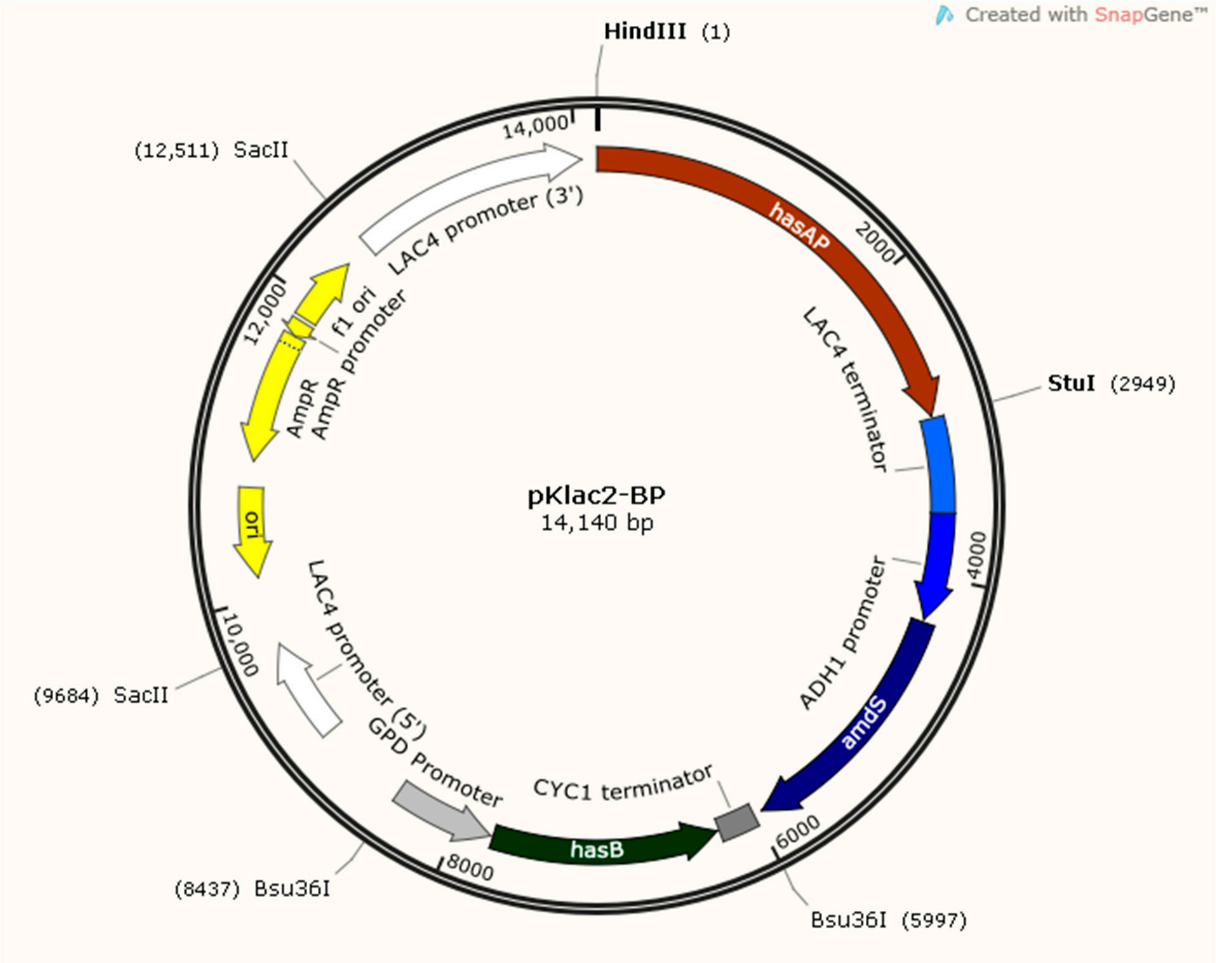

Figure S09. Map of the plasmid pKlac2-B1 used in this study for integration of the *hasB* gene (UDP-Glucose Dehydrogenase) from *Xenopus laevis* and *hasA1* gene (Hyaluronic Acid Synthase) Isoform 1 from *Homo sapiens* in genome of *Kluyveromyces lactis*.

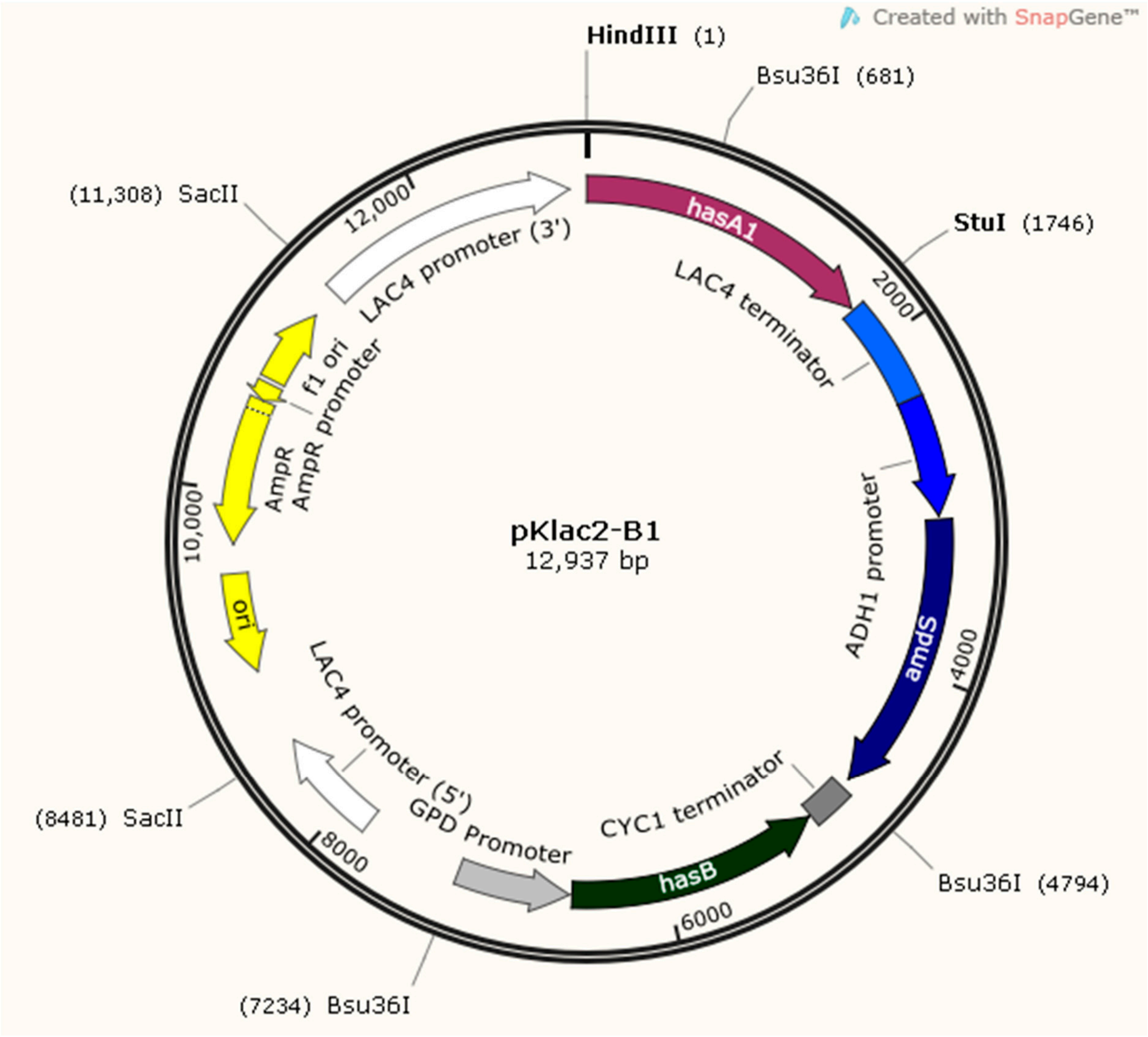

Figure S10. Map of the plasmid pKlac2-B2 used in this study for integration of the *hasB* gene (UDP-Glucose Dehydrogenase) from *Xenopus laevis* and *hasA2* gene (Hyaluronic Acid Synthase) Isoform 2 from *Homo sapiens* in genome of *Kluyveromyces lactis*.

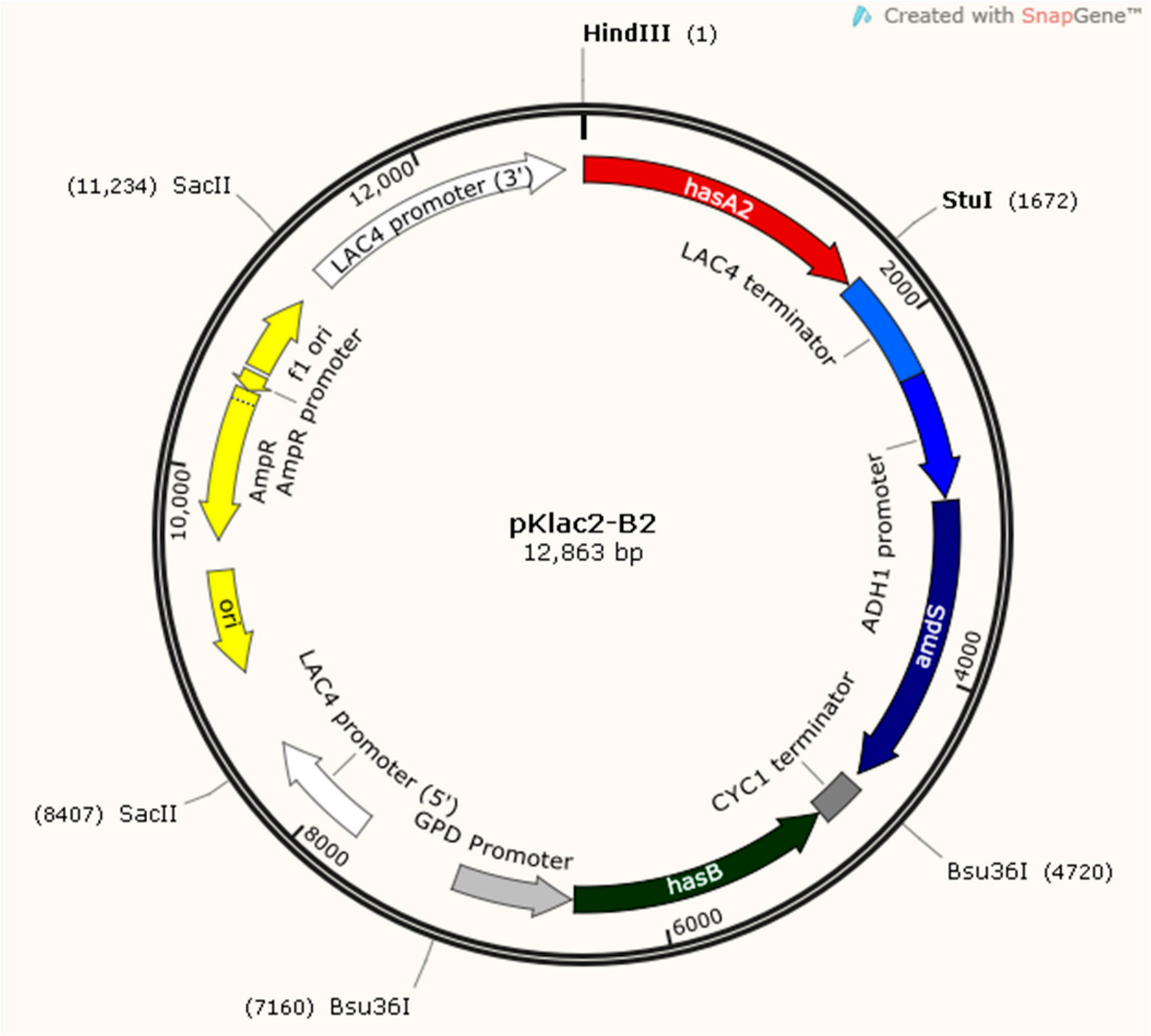

Figure S11. Map of the plasmid pKlac2-B3 used in this study for integration of the *hasB* gene (UDP-Glucose Dehydrogenase) from *Xenopus laevis* and *hasA3* gene (Hyaluronic Acid Synthase) Isoform 3 from *Homo sapiens* in genome of *Kluyveromyces lactis*.

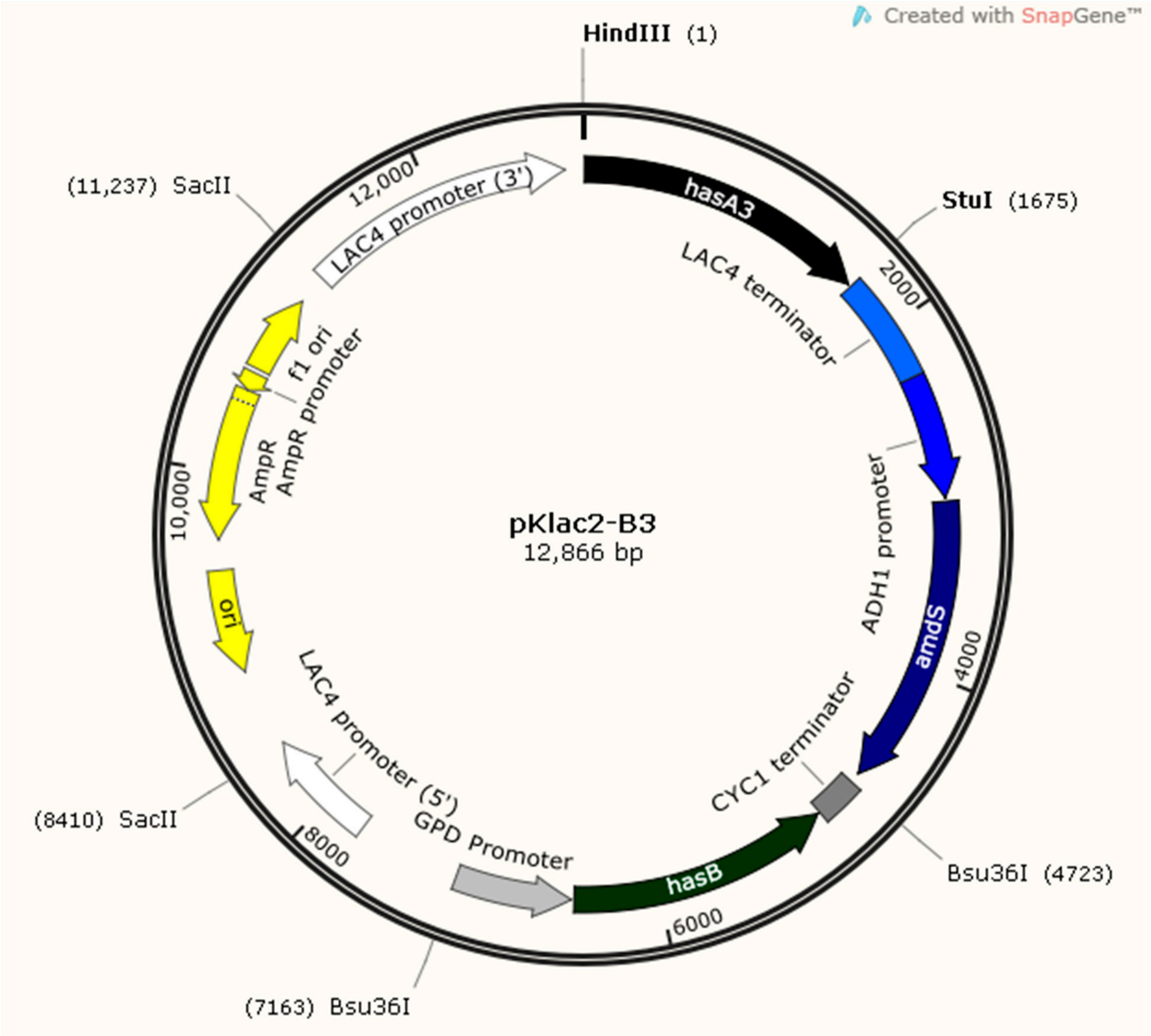

Sequence S01. Sequence of the 4409 PB plasmid pBSK-HASB. The Coding sequence (CDS) of *hasB* gene is highlighted in yellow.

CTGACGCGCCCTGTAGCGGCGCATTAAGCGCGGCGGGTGTGGTGGTTACGCGCAGCGTGACCGCT
ACACTTGCCAGCGCCCTAGCGCCCGCTCCTTTCGCTTTCTTCCCTTCCTTTCTCGCCACGTTCGCCG
GCTTTCCCCGTCAAGCTCTAAATCGGGGGCTCCCTTTAGGGTTCCGATTTAGTGCTTTACGGCACCT
CGACCCCAAAAAACTTGATTAGGGTGATGGTTCACGTAGTGGGCCATCGCCCTGATAGACGGTTTT
TCGCCCTTTGACGTTGGAGTCCACGTTCTTTAATAGTGGACTCTTGTTCCAAACTGGAACAACACT
CAACCCTATCTCGGTCTATTCTTTTGATTTATAAGGGATTTTGCCGATTTCGGCCTATTGGTTAAAAA
ATGAGCTGATTTAACAAAAATTTAACGCGAATTTTAACAAAATATTAACGCTTACAATTTGCCATTC
GCCATTCAGGCTGCGCAACTGTTGGGAAGGGCGATCGGTGCGGGCCTCTTCGCTATTACGCCAGC
TGGCGAAAGGGGGATGTGCTGCAAGGCGATTAAGTTGGGTAACGCCAGGGTTTTCCCAGTCACGA
CGTTGTAAAACGACGGCCAGTGAATTGTAATACGACTCACTATAGGGCGAATTGGGTACCGGGCC
CCCCCTCGAGGTCGACTTACACACGTTGCTTCTTGTGTGGCAAGTCCTGTAAACCGAACTTAGGGA
TATCAGCAGTTGGAGTGAATGGTATTCTTTTTGAAGCTACCTTCTTTCCGATGGTTTCCACCTGAAA
TCCAATGTTTTGCAATTCTCCATGCAATTCATCTAAAACACGTCTACCATCGAATATGAAAGCAGG
CTTTAACATCATCCTATGGATTCTATTGAAATCTAATTCCTTGAACATATCCCATTCAGTACAAATGA
CCATAGCGTGTGCATTCTCACAGGCTTCGTACAAATCTGTACTTATGTGGACCAATTGAGAAACCC
TGTCGTCAGCTGCAACACCAGGTTGACTCAAGTCAGTGATGATCTGCTCACGTGGGACCTTTGGAT
CGTAGATATGTAACTTAGCACCTTCATCCATCAAATACTTAGAGATATAGATTGAACTACTCTCTCT
AGTATCACCTGTATCCTTCTTGAAAGCGAAACCTAACAATGCGATTTTCTTATCGGTCACGGTGTTA
AACAAACAATCGATTATCCTAGTTGTAAAACGTCTCCTTTGATAATCATTCATATCAATCACTTGTT
GCCAGTACTTGGCCACTTCGTGCAAGTTTAACACCTCACACAAGTAAACCAAGTTCAAAACGTCC
TTCTGAAAACATGAACCTCCAAATCCCACTGAAGCCTTCAAAAACTTGTTACCAATTCTTTGATCC
ATACCAATAGCTCTGGCAACCTCTTCAACGTCAGCTCCTGTGGCTTCACATAAGGCACTAATTGAG
TTGATTGAAGAAATTCTTTGAGCTAAGAATGCGTTGGCTGCTAACTTACTCAACTCAGAAGACCAG
GTGTTTGTGGTTATGATTTTCTCAGATGGTACCCAGTGTTCGTATACGTCACACAAAGCTCTAACAG
CTTTCTGACCTTCAGGGGTTTCGTCACCACCTATCAAAACTCTATCAGGGTTCTTCAAATCCTTAAT
GGCTGTACCCTCTGCCAAAAACTCTGGGTTACTCAATACCTGCAAGTTCAAATCTGGTTTAGTATTT
GCATCGAAGATACGTCTTATTGATTCAGCAGCTCTAACTGGCACAGTAGATTTCTCTGTAACAATCT
TGTATCCGTTACTATTCTGTACTATTCTTCTAGCGCAAGCCTCAATGTATTTCAAGTCGGCTGCCCTT
CCCTTACCCATACCGTAAGTTTTTGTTGGAGTGTTGACTGAGATGAACACCAAATCAGCTTCTTGA
ATTGCACCATCAATGTCAGTTGAGTAGAACAAATTCTTTCCCCTGCATGACTCTACGACTTCCTTCA
AACCTGGTTCGTAGATAGGCAAAGTGTCACTATTCCAAGCATTGATCCTGGCTTGGTTCACATCCA
CAACAGTGACCTTAATGTCAGGGCACATCTGTGCAATGACAGAACAGGTTGGACCACCGACGTA
ACCGGCACCAATACAACAAATCTTCTTGATCTGAAACATTTTTGGATCCACTAGTTCTAGAGCGGC
CGCCACCGCGGTGGAGCTCCAGCTTTTGTTCCCTTTAGTGAGGGTTAATTTCGAGCTTGGCGTAAT
CATGGTCATAGCTGTTTCCTGTGTGAAATTGTTATCCGCTCACAATTCCACACAACATACGAGCCG
GAAGCATAAAGTGTAAAGCCTGGGGTGCCTAATGAGTGAGCTAACTCACATTAATTGCGTTGCGC
TCACTGCCCGCTTTCCAGTCGGGAAACCTGTCGTGCCAGCTGCATTAATGAATCGGCCAACGCGC
GGGGAGAGGCGGTTTGCGTATTGGGCGCTCTTCCGCTTCCTCGCTCACTGACTCGCTGCGCTCGGT
CGTTCGGCTGCGGCGAGCGGTATCAGCTCACTCAAAGGCGGTAATACGGTTATCCACAGAATCAG
GGGATAACGCAGGAAAGAACATGTGAGCAAAAGGCCAGCAAAAGGCCAGGAACCGTAAAAAG
GCCGCGTTGCTGGCGTTTTTCCATAGGCTCCGCCCCCCTGACGAGCATCACAAAAATCGACGCTC
AAGTCAGAGGTGGCGAAACCCGACAGGACTATAAAGATACCAGGCGTTTCCCCCTGGAAGCTCC

CTCGTGCGCTCTCCTGTTCCGACCCTGCCGCTTACCGGATACCTGTCCGCCTTTCTCCCTTCGGGAA
GCGTGGCGCTTTCTCATAGCTCACGCTGTAGGTATCTCAGTTCGGTGTAGGTCGTTCGCTCCAAGCT
GGGCTGTGTGCACGAACCCCCCGTTCAGCCCGACCGCTGCGCCTTATCCGGTAACTATCGTCTTGA
GTCCAACCCGGTAAGACACGACTTATCGCCACTGGCAGCAGCCACTGGTAACAGGATTAGCAGA
GCGAGGTATGTAGGCGGTGCTACAGAGTTCTTGAAGTGGTGGCCTAACTACGGCTACACTAGAAG
GACAGTATTTGGTATCTGCGCTCTGCTGAAGCCAGTTACCTTCGGAAAAAGAGTTGGTAGCTCTTG
ATCCGGCAAACAAACCACCGCTGGTAGCGGTGGTTTTTTTGTTTGCAAGCAGCAGATTACGCGCA
GAAAAAAAGGATCTCAAGAAGATCCTTTGATCTTTTCTACGGGGTCTGACGCTCAGTGGAACGAA
AACTCACGTTAAGGGATTTTGGTCATGAGATTATCAAAAAGGATCTTCACCTAGATCCTTTTAAATT
AAAAATGAAGTTTTAAATCAATCTAAAGTATATATGAGTAAACTTGGTCTGACAGTTACCAATGCT
TAATCAGTGAGGCACCTATCTCAGCGATCTGTCTATTTCGTTCATCCATAGTTGCCTGACTCCCCGT
CGTGTAGATAACTACGATACGGGAGGGCTTACCATCTGGCCCCAGTGCTGCAATGATACCGCGAG
ACCCACGCTCACCGGCTCCAGATTTATCAGCAATAAACCAGCCAGCCGGAAGGGCCGAGCGCAG
AAGTGGTCCTGCAACTTTATCCGCCTCCATCCAGTCTATTAATTGTTGCCGGGAAGCTAGAGTAAG
TAGTTCGCCAGTTAATAGTTTGCGCAACGTTGTTGCCATTGCTACAGGCATCGTGGTGTCACGCTC
GTCGTTTGGTATGGCTTCATTCAGCTCCGGTTCCCAACGATCAAGGCGAGTTACATGATCCCCCAT
GTTGTGCAAAAAAGCGGTTAGCTCCTTCGGTCCTCCGATCGTTGTCAGAAGTAAGTTGGCCGCAG
TGTTATCACTCATGGTTATGGCAGCACTGCATAATTCTCTTACTGTCATGCCATCCGTAAGATGCTTT
TCTGTGACTGGTGAGTACTCAACCAAGTCATTCTGAGAATAGTGTATGCGGCGACCGAGTTGCTCT
TGCCCGGCGTCAATACGGGATAATACCGCGCCACATAGCAGAACTTTAAAAGTGCTCATCATTGG
AAAACGTTCTTCGGGGCGAAAACTCTCAAGGATCTTACCGCTGTTGAGATCCAGTTCGATGTAAC
CCACTCGTGCACCCAACTGATCTTCAGCATCTTTTACTTTCACCAGCGTTTCTGGGTGAGCAAAAA
CAGGAAGGCAAAATGCCGCAAAAAAGGGAATAAGGGCGACACGGAAATGTTGAATACTCATACT
CTTCCTTTTTCAATATTATTGAAGCATTTATCAGGGTTATTGTCTCATGAGCGGATACATATTTGAAT
GTATTTAGAAAAATAAACAAATAGGGGTTCCGCGCACATTTCCCCGAAAAGTGCCAC

Sequence S02. Sequence of the 7947 PB plasmid p424-GPD-B. The sequence of GPD Promoter and CYC1 terminator is highlighted in yellow and the coding sequence of *hasB* gene is highlighted in gray.

TCGCGCGTTTCGGTGATGACGGTGAAAACCTCTGACACATGCAGCTCCCGGAGACGGTCACAGCT
TGTCTGTAAGCGGATGCCGGGAGCAGACAAGCCCGTCAGGGCGCGTCAGCGGGTGTTGGCGGGT
GTCGGGGCTGGCTTAACTATGCGGCATCAGAGCAGATTGTACTGAGAGTGCACCATAAACGACAT
TACTATATATATAATATAGGAAGCATTTAATAGACAGCATCGTAATATATGTGTACTTTGCAGTTATG
ACGCCAGATGGCAGTAGTGGAAGATATTCTTTATTGAAAAATAGCTTGTCACCTTACGTACAATCT
TGATCCGGAGCTTTTCTTTTTTTGCCGATTAAGAATTAATTCGGTCGAAAAAAGAAAAGGAGAGG
GCCAAGAGGGAGGGCATTGGTGACTATTGAGCACGTGAGTATACGTGATTAAGCACACAAAGGC
AGCTTGGAGTATGTCTGTTATTAATTTCACAGGTAGTTCTGGTCCATTGGTGAAAGTTTGCGGCTTG
CAGAGCACAGAGGCCGCAGAATGTGCTCTAGATTCCGATGCTGACTTGCTGGGTATTATATGTGTG
CCCAATAGAAAGAGAACAATTGACCCGGTTATTGCAAGGAAAATTTCAAGTCTTGTAAAAGCATA
TAAAAATAGTTCAGGCACTCCGAAATACTTGGTTGGCGTGTTTCGTAATCAACCTAAGGAGGATGT
TTTGGCTCTGGTCAATGATTACGGCATTGATATCGTCCAACTGCATGGAGATGAGTCGTGGCAAGA
ATACCAAGAGTTCCTCGGTTTGCCAGTTATTAAAAGACTCGTATTTCCAAAAGACTGCAACATACT
ACTCAGTGCAGCTTCACAGAAACCTCATTCGTTTATTCCCTTGTTTGATTCAGAAGCAGGTGGGAC

AGGTGAACTTTTGGATTGGAACTCGATTTCTGACTGGGTTGGAAGGCAAGAGAGCCCCGAAAGCT
TACATTTTATGTTAGCTGGTGGACTGACGCCAGAAAATGTTGGTGATGCGCTTAGATTAAATGGCG
TTATTGGTGTTGATGTAAGCGGAGGTGTGGAGACAAATGGTGTAAAAGACTCTAACAAAATAGCA
AATTTCGTCAAAAATGCTAAGAAATAGGTTATTACTGAGTAGTATTTATTTAAGTATTGTTTGTGCAC
TTGCCTATGCGGTGTGAAATACCGCACAGATGCGTAAGGAGAAAATACCGCATCAGGAAATTGTA
AACGTTAATATTTTGTTAAAATTCGCGTTAAATTTTTGTTAAATCAGCTCATTTTTTAACCAATAGGC
CGAAATCGGCAAAATCCCTTATAAATCAAAAGAATAGACCGAGATAGGGTTGAGTGTTGTTCCAG
TTTGGAACAAGAGTCCACTATTAAAGAACGTGGACTCCAACGTCAAAGGGCGAAAAACCGTCTA
TCAGGGCGATGGCCCACTACGTGAACCATCACCCTAATCAAGTTTTTTGGGGTCGAGGTGCCGTA
AAGCACTAAATCGGAACCCTAAAGGGAGCCCCCGATTTAGAGCTTGACGGGGAAAGCCGGCGAA
CGTGGCGAGAAAGGAAGGGAAGAAAGCGAAAGGAGCGGGCGCTAGGGCGCTGGCAAGTGTAG
CGGTCACGCTGCGCGTAACCACCACACCCGCCGCGCTTAATGCGCCGCTACAGGGCGCGTCGCGC
CATTCGCCATTCAGGCTGCGCAACTGTTGGGAAGGGCGATCGGTGCGGGCCTCTTCGCTATTACGC
CAGCTGGCGAAAGGGGGATGTGCTGCAAGGCGATTAAGTTGGGTAACGCCAGGGTTTTCCCAGTC
ACGACGTTGTAAAACGACGGCCAGTGAGCGCGCGTAATACGACTCACTATAGGGCGAATTGGGTA
CCGGCCGCAAATTAAAGCCTTCGAGCGTCCCAAAACCTTCTCAAGCAAGGTTTTCAGTATAATGTT
ACATGCGTACACGCGTCTGTACAGAAAAAAAAGAAAAATTTGAAATATAAATAACGTTCTTAATA
CTAACATAACTATAAAAAAATAAATAGGGACCTAGACTTCAGGTTGTCTAACTCCTTCCTTTTCGGT
TAGAGCGGATGTGGGGGGAGGGCGTGAATGTAAGCGTGACATAACTAATTACATGACTCGAGGTC
GACTTACACACGTTGCTTCTTGTGTGGCAAGTCCTGTAAACCGAACTTAGGGATATCAGCAGTTGG
AGTGAATGGTATTCTTTTTGAAGCTACCTTCTTTCCGATGGTTTCCACCTGAAATCCAATGTTTTGCA
ATTCTCCATGCAATTCATCTAAAACACGTCTACCATCGAATATGAAAGCAGGCTTTAACATCATCCT
ATGGATTCTATTGAAATCTAATTCCTTGAACATATCCCATTCAGTACAAATGACCATAGCGTGTGCA
TTCTCACAGGCTTCGTACAAATCTGTACTTATGTGGACCAATTGAGAAACCCTGTCGTCAGCTGCA
ACACCAGGTTGACTCAAGTCAGTGATGATCTGCTCACGTGGGACCTTTGGATCGTAGATATGTAAC
TTAGCACCTTCATCCATCAAATACTTAGAGATATAGATTGAACTACTCTCTCTAGTATCACCTGTATC
CTTCTTGAAAGCGAAACCTAACAATGCGATTTTCTTATCGGTCACGGTGTTAAACAAACAATCGAT
TATCCTAGTTGTAAAACGTCTCCTTTGATAATCATTCATATCAATCACTTGTTGCCAGTACTTGGCCA
CTTCGTGCAAGTTTAACACCTCACACAAGTAAACCAAGTTCAAAACGTCCTTCTGAAAACATGAA
CCTCCAAATCCCACTGAAGCCTTCAAAAACTTGTTACCAATTCTTTGATCCATACCAATAGCTCTG
GCAACCTCTTCAACGTCAGCTCCTGTGGCTTCACATAAGGCACTAATTGAGTTGATTGAAGAAATT
CTTTGAGCTAAGAATGCGTTGGCTGCTAACTTACTCAACTCAGAAGACCAGGTGTTTGTGGTTATG
ATTTTCTCAGATGGTACCCAGTGTTCGTATACGTCACACAAAGCTCTAACAGCTTTCTGACCTTCAG
GGGTTTCGTCACCACCTATCAAAACTCTATCAGGGTTCTTCAAATCCTTAATGGCTGTACCCTCTGC
CAAAAACTCTGGGTTACTCAATACCTGCAAGTTCAAATCTGGTTTAGTATTTGCATCGAAGATACG
TCTTATTGATTCAGCAGCTCTAACTGGCACAGTAGATTTCTCTGTAACAATCTTGTATCCGTTACTAT
TCTGTACTATTCTTCTAGCGCAAGCCTCAATGTATTTCAAGTCGGCTGCCCTTCCCTTACCCATACC
GTAAGTTTTTGTTGGAGTGTTGACTGAGATGAACACCAAATCAGCTTCTTGAATTGCACCATCAAT
GTCAGTTGAGTAGAACAAATTCTTTCCCCTGCATGACTCTACGACTTCCTTCAAACCTGGTTCGTA
GATAGGCAAAGTGTCACTATTCCAAGCATTGATCCTGGCTTGGTTCACATCCACAACAGTGACCTT
AATGTCAGGGCACATCTGTGCAATGACAGAACAGGTTGGACCACCGACGTAACCGGCACCAATA
CAACAAATCTTCTTGATCTGAAACATTTTTGGATCCACTAGTTCTAGATCCGTCGAAACTAAGTTCT
TGGTGTTTTAAAACTAAAAAAAAGACTAACTATAAAAGTAGAATTTAAGAAGTTTAAGAAATAGA
TTTACAGAATTACAATCAATACCTACCGTCTTTATATACTTATTAGTCAAGTAGGGGAATAATTTCAG

GGAACTGGTTTAAACCTTTTTTTTCAGCTTTTTCCAAATCAGAGAGAGCAGAAGGTAATAGAAGGT
GTAAGAAAATGAGATAGATACATGCGTGGGTCAATTGCCTTGTGTCATCATTTACTCCAGGCAGGT
TGCATCACTCCATTGAGGTTGTGCCCGTTTTTTGCCTGTTTGTGCCCTGTTCTCTGTAGTTGCGCTAA
GAGAATGGACCTATGAACTGATGGTTGGTGAAGAAAACAATATTTTGGTGCTGGGATTCTTTTTTT
TCTGGATGCCAGCTTAAAAAGCGGGCTCCATTATATTTAGTGGATGCCAGGAATAAACCTGTTCAC
CCAAGCACCATCAGTGTTATATATTCTGTGTAACCCGCCCCCTATTTTGGCATGTACGGGTTACAGC
AGAATTAAAAGGCTAATTTTTTGACTAAATAAAGTTAGGAAAATCACTACTATTAATTATTTACGTA
TTCTTTGAAATGGCAGTATTGATAATGATAAACTCGAGAGCTCCAGCTTTTGTTCCCTTTAGTGAGG
GTTAATTGCGCGCTTGGCGTAATCATGGTCATAGCTGTTTCCTGTGTGAAATTGTTATCCGCTCACA
ATTCCACACAACATAGGAGCCGGAAGCATAAAGTGTAAAGCCTGGGGTGCCTAATGAGTGAGGT
AACTCACATTAATTGCGTTGCGCTCACTGCCCGCTTTCCAGTCGGGAAACCTGTCGTGCCAGCTGC
ATTAATGAATCGGCCAACGCGCGGGGAGAGGCGGTTTGCGTATTGGGCGCTCTTCCGCTTCCTCGC
TCACTGACTCGCTGCGCTCGGTCGTTCGGCTGCGGCGAGCGGTATCAGCTCACTCAAAGGCGGTA
ATACGGTTATCCACAGAATCAGGGGATAACGCAGGAAAGAACATGTGAGCAAAAGGCCAGCAA
AAGGCCAGGAACCGTAAAAAGGCCGCGTTGCTGGCGTTTTTCCATAGGCTCCGCCCCCCTGACGA
GCATCACAAAAATCGACGCTCAAGTCAGAGGTGGCGAAACCCGACAGGACTATAAAGATACCAG
GCGTTTCCCCCTGGAAGCTCCCTCGTGCGCTCTCCTGTTCCGACCCTGCCGCTTACCGGATACCTGT
CCGCCTTTCTCCCTTCGGGAAGCGTGGCGCTTTCTCATAGCTCACGCTGTAGGTATCTCAGTTCGGT
GTAGGTCGTTCGCTCCAAGCTGGGCTGTGTGCACGAACCCCCCGTTCAGCCCGACCGCTGCGCCT
TATCCGGTAACTATCGTCTTGAGTCCAACCCGGTAAGACACGACTTATCGCCACTGGCAGCAGCC
ACTGGTAACAGGATTAGCAGAGCGAGGTATGTAGGCGGTGCTACAGAGTTCTTGAAGTGGTGGCC
TAACTACGGCTACACTAGAAGGACAGTATTTGGTATCTGCGCTCTGCTGAAGCCAGTTACCTTCGG
AAAAAGAGTTGGTAGCTCTTGATCCGGCAAACAAACCACCGCTGGTAGCGGTGGTTTTTTTGTTTG
CAAGCAGCAGATTACGCGCAGAAAAAAAGGATCTCAAGAAGATCCTTTGATCTTTTCTACGGGGT
CTGACGCTCAGTGGAACGAAAACTCACGTTAAGGGATTTTGGTCATGAGATTATCAAAAAGGATC
TTCACCTAGATCCTTTTAAATTAAAAATGAAGTTTTAAATCAATCTAAAGTATATATGAGTAAACTT
GGTCTGACAGTTACCAATGCTTAATCAGTGAGGCACCTATCTCAGCGATCTGTCTATTTCGTTCATC
CATAGTTGCCTGACTCCCCGTCGTGTAGATAACTACGATACGGGAGGGCTTACCATCTGGCCCCAG
TGCTGCAATGATACCGCGAGACCCACGCTCACCGGCTCCAGATTTATCAGCAATAAACCAGCCAG
CCGGAAGGGCCGAGCGCAGAAGTGGTCCTGCAACTTTATCCGCCTCCATCCAGTCTATTAATTGTT
GCCGGGAAGCTAGAGTAAGTAGTTCGCCAGTTAATAGTTTGCGCAACGTTGTTGCCATTGCTACAG
GCATCGTGGTGTCACGCTCGTCGTTTGGTATGGCTTCATTCAGCTCCGGTTCCCAACGATCAAGGC
GAGTTACATGATCCCCCATGTTGTGCAAAAAAGCGGTTAGCTCCTTCGGTCCTCCGATCGTTGTCA
GAAGTAAGTTGGCCGCAGTGTTATCACTCATGGTTATGGCAGCACTGCATAATTCTCTTACTGTCAT
GCCATCCGTAAGATGCTTTTCTGTGACTGGTGAGTACTCAACCAAGTCATTCTGAGAATAGTGTAT
GCGGCGACCGAGTTGCTCTTGCCCGGCGTCAATACGGGATAATACCGCGCCACATAGCAGAACTT
TAAAAGTGCTCATCATTGGAAAACGTTCTTCGGGGCGAAAACTCTCAAGGATCTTACCGCTGTTG
AGATCCAGTTCGATGTAACCCACTCGTGCACCCAACTGATCTTCAGCATCTTTTACTTTCACCAGC
GTTTCTGGGTGAGCAAAAACAGGAAGGCAAAATGCCGCAAAAAAGGGAATAAGGGCGACACGG
AAATGTTGAATACTCATACTCTTCCTTTTTCAATATTATTGAAGCATTTATCAGGGTTATTGTCTCATG
AGCGGATACATATTTGAATGTATTTAGAAAAATAAACAAATAGGGGTTCCGCGCACATTTCCCCGA
AAAGTGCCACCTGAACGAAGCATCTGTGCTTCATTTTGTAGAACAAAAATGCAACGCGAGAGCG
CTAATTTTTCAAACAAAGAATCTGAGCTGCATTTTTACAGAACAGAAATGCAACGCGAAAGCGCT
ATTTTACCAACGAAGAATCTGTGCTTCATTTTTGTAAAACAAAAATGCAACGCGAGAGCGCTAATT

TTTCAAACAAAGAATCTGAGCTGCATTTTTACAGAACAGAAATGCAACGCGAGAGCGCTATTTTA
CCAACAAAGAATCTATACTTCTTTTTTGTTCTACAAAAATGCATCCCGAGAGCGCTATTTTTCTAAC
AAAGCATCTTAGATTACTTTTTTTCTCCTTTGTGCGCTCTATAATGCAGTCTCTTGATAACTTTTTGCA
CTGTAGGTCCGTTAAGGTTAGAAGAAGGCTACTTTGGTGTCTATTTTCTCTTCCATAAAAAAAGCCT
GACTCCACTTCCCGCGTTTACTGATTACTAGCGAAGCTGCGGGTGCATTTTTTCAAGATAAAGGCA
TCCCCGATTATATTCTATACCGATGTGGATTGCGCATACTTTGTGAACAGAAAGTGATAGCGTTGAT
GATTCTTCATTGGTCAGAAAATTATGAACGGTTTCTTCTATTTTGTCTCTATATACTACGTATAGGAA
ATGTTTACATTTTCGTATTGTTTTCGATTCACTCTATGAATAGTTCTTACTACAATTTTTTTGTCTAAA
GAGTAATACTAGAGATAAACATAAAAAAATGTAGAGGTCGAGTTTAGATGCAAGTTCAAGGAGCGA
AAGGTGGATGGGTAGGTTATATAGGGATATAGCACAGAGATATATAGCAAAGAGATACTTTTGAGC
AATGTTTGTGGAAGCGGTATTCGCAATATTTTAGTAGCTCGTTACAGTCCGGTGCGTTTTTGGTTTTT
TGAAAGTGCGTCTTCAGAGCGCTTTTGGTTTTCAAAAGCGCTCTGAAGTTCCTATACTTTCTAGAG
AATAGGAACTTCGGAATAGGAACTTCAAAGCGTTTCCGAAAACGAGCGCTTCCGAAAATGCAAC
GCGAGCTGCGCACATACAGCTCACTGTTCACGTCGCACCTATATCTGCGTGTTGCCTGTATATATAT
ATACATGAGAAGAACGGCATAGTGCGTGTTTATGCTTAAATGCGTACTTATATGCGTCTATTTATGTA
GGATGAAAGGTAGTCTAGTACCTCCTGTGATATTATCCCATTCCATGCGGGGTATCGTATGCTTCCTT
CAGCACTACCCTTTAGCTGTTCTATATGCTGCCACTCCTCAATTGGATTAGTCTCATCCTTCAATGCT
ATCATTTCCTTTGATATTGGATCATATTAAGAAACCATTATTATCATGACATTAACCTATAAAAATAG
GCGTATCACGAGGCCCTTTCGTC

Sequence S03. Sequence of the 5815 PB plasmid pBSK-HASAP. The sequence of the coding Sequence of *hasAP* gene is highlighted in yellow.

CTGACGCGCCCTGTAGCGGCGCATTAAGCGCGGCGGGTGTGGTGGTTACGCGCAGCGTGACCGCT
ACACTTGCCAGCGCCCTAGCGCCCGCTCCTTTCGCTTTCTTCCCTTCCTTTCTCGCCACGTTCGCCG
GCTTTCCCCGTCAAGCTCTAAATCGGGGGCTCCCTTTAGGGTTCCGATTTAGTGCTTTACGGCACCT
CGACCCCAAAAAACTTGATTAGGGTGATGGTTCACGTAGTGGGCCATCGCCCTGATAGACGGTTTT
TCGCCCTTTGACGTTGGAGTCCACGTTCTTTAATAGTGGACTCTTGTTCCAAACTGGAACAACACT
CAACCCTATCTCGGTCTATTCTTTTGATTTATAAGGGATTTTGCCGATTTCGGCCTATTGGTTAAAAA
ATGAGCTGATTTAACAAAAATTTAACGCGAATTTTAACAAAATATTAACGCTTACAATTTGCCATTC
GCCATTCAGGCTGCGCAACTGTTGGGAAGGGCGATCGGTGCGGGCCTCTTCGCTATTACGCCAGC
TGGCGAAAGGGGGATGTGCTGCAAGGCGATTAAGTTGGGTAACGCCAGGGTTTTCCCAGTCACGA
CGTTGTAAAACGACGGCCAGTGAATTGTAATACGACTCACTATAGGGCGACCCTTTAAGCTTATGA
ATACCTTATCTCAAGCCATCAAGGCATACAATTCAAATGACTATCAATTGGCTTTGAAATTGTTCGA
AAAGTCAGCAGAAATCTACGGACGTAAGATAGTAGAGTTTCAGATTACTAAGTGCAAGGAGAAAT
TGTCTGCCCATCCAAGTGTCAATTCAGCTCATCCTTCAGTCAACTCAGCACATTTGTCAGTAAACA
AAGAGGAAAAGGTTAATGTGTGCGATTCTCCATTGGATATTGCTACCCAATTGTTGTTGTCAAATGT
CAAGAAATTAGTGTTATCTGACAGTGAGAAGAACACATTGAAGAATAAGTGGAAGTTGTTAACTG
AAAAGAAAAGTGAGAATGCTGAAGTTCGTGCTGTAGCATTGGTACCAAAGGATTTCCCAAAGGAT
TTGGTGTTGGCACCATTACCTGACCATGTGAATGACTTTACTTGGTACAAGAAGAGAAAGAAACG
TTTAGGTATCAAACCAGAACATCAACACGTGGGTTTGTCAATAATTGTCACCACTTTCAATCGTCC
TGCAATCTTAAGTATAACTTTGGCATGCTTAGTTAATCAAAAGACTCACTATCCATTCGAGGTGATT
GTCACAGATGATGGATCACAAGAAGATTTGTCTCCAATCATAAGACAATATGAAAACAAATTGGA
TATCCGTTATGTCAGACAAAAGGACAATGGTTTCCAAGCTAGTGCTGCTAGGAATATGGGTTTGAG
ATTAGCAAAGTATGATTTCATTGGTTTGTTGGATTGCGATATGGCACCTAACCCATTATGGGTGCATT

CATATGTCGCTGAATTGTTAGAAGATGATGATTTGACAATCATTGGACCAAGAAAGTACATTGATA
CACAACATATCGACCCAAAGGACTTCTTAAACAATGCATCTTTGTTGGAATCATTGCCAGAAGTTA
AGACCAATAACTCAGTGGCCGCAAAAGGTGAAGGTACCGTTTCATTGGATTGGAGGTTGGAGCA
ATTCGAAAAGACTGAAAACTTAAGATTGTCAGACTCTCCTTTTAGATTCTTCGCAGCTGGTAATGT
TGCTTTCGCCAAGAAGTGGTTGAACAAATCTGGATTCTTTGATGAAGAGTTCAACCATTGGGGTGG
TGAAGATGTTGAGTTTGGATATAGATTGTTTAGGTATGGTTCATTCTTCAAGACTATTGACGGTATCA
TGGCCTACCATCAAGAGCCACCTGGTAAGGAAAACGAAACAGATAGGGAAGCTGGAAAGAACA
TCACATTGGATATTATGAGGGAGAAGGTACCATATATTTACAGGAAGTTGTTGCCTATCGAAGATTC
ACACATCAATAGAGTCCCTTTGGTTTCTATCTATATCCCAGCTTACAACTGTGCCAATTATATTCAAC
GTTGTGTTGATTCTGCCTTGAACCAGACAGTTGTAGATTTGGAAGTCTGTATTTGCAATGATGGTTC
TACAGATAATACTTTGGAAGTTATCAACAAGTTGTACGGTAACAATCCAAGAGTCAGAATCATGA
GTAAACCAAATGGTGGTATTGCTAGTGCTTCTAATGCAGCAGTGAGTTTTGCCAAAGGATATTACA
TAGGTCAATTAGATTCAGATGACTATTTGGAGCCAGATGCCGTAGAGTTATGTTTGAAAGAGTTCTT
GAAAGACAAAACTTTGGCTTGTGTATATACAACAAACAGAAATGTCAATCCTGATGGTTCTTTGAT
AGCAAATGGTTACAACTGGCCAGAGTTTAGTAGGGAGAAGTTGACTACTGCAATGATTGCTCATC
ACTTCCGTATGTTCACTATCAGGGCATGGCATTTGACCGATGGTTTTAATGAGAAGATTGAGAATG
CTGTGGACTACGATATGTTCTTGAAGTTGAGTGAAGTTGGTAAGTTCAAGCACTTAAACAAAATCT
GCTATAACAGGGTATTGCATGGTGATAATACAAGTATTAAGAAGTTGGGTATCCAAAAGAAGAAC
CATTTCGTGGTCGTCAACCAGAGTTTGAACAGGCAAGGAATCACTTACTACAATTACGACGAGTT
CGATGACTTAGATGAGTCTAGGAAATACATCTTTAACAAAACAGCTGAGTACCAGGAAGAAATTG
ACATCTTAAAGGACATTAAGATCATACAAAACAAGGACGCTAAAATAGCAGTATCTATCTTCTACC
CAAATACTTTGAATGGTTTGGTCAAGAAATTGAATAACATCATCGAGTACAACAAGAACATATTCG
TTATTGTCTTGCATGTGGACAAGAACCATTTGACCCCAGATATCAAGAAAGAGATATTGGCTTTCT
ACCACAAGCATCAAGTGAATATTTTGTTGAATAACGATATCTCATACTACACATCAAACCGTTTAAT
CAAGACCGAGGCACATTTATCAAACATTAATAAGTTGTCACAGTTGAACTTGAATTGTGAATATAT
CATATTCGACAATCATGACTCTTTGTTCGTGAAGAATGATTCTTATGCCTATATGAAGAAGTACGAT
GTTGGTATGAATTTCTCAGCCTTAACTCATGATTGGATTGAAAAGATTAACGCACATCCACCATTCA
AGAAGTTGATTAAGACATACTTTAACGATAATGACTTGAAATCTATGAACGTTAAAGGAGCTAGTC
AAGGAATGTTTATGACATATGCATTGGCTCACGAATTGTTGACTATTATCAAAGAGGTTATCACTTC
TTGCCAATCTATCGATTCTGTACCAGAATACAACACTGAGGACATATGGTTTCAATTTGCATTGTTG
ATCTTGGAAAAGAAAACTGGTCATGTCTTTAACAAGACAAGTACCTTGACATACATGCCTTGGGA
GAGGAAGTTGCAATGGACCAATGAACAAATTGAATCAGCTAAACGTGGAGAAAACATTCCAGTG
AACAAGTTCATAATCAATTCAATCACATTGTAAAGGCCTAAAGGGGATATCCTCGAGGTTCCCTTT
AGTGAGGGTTAATTGCGAGCTTGGCGTAATCATGGTCATAGCTGTTTCCTGTGTGAAATTGTTATCC
GCTCACAATTCCACACAACATACGAGCCGGAAGCATAAAGTGTAAAGCCTGGGGTGCCTAATGA
GTGAGCTAACTCACATTAATTGCGTTGCGCTCACTGCCCGCTTTCCAGTCGGGAAACCTGTCGTGC
CAGCTGCATTAATGAATCGGCCAACGCGCGGGGAGAGGCGGTTTGCGTATTGGGCGCTCTTCCGC
TTCCTCGCTCACTGACTCGCTGCGCTCGGTCGTTCGGCTGCGGCGAGCGGTATCAGCTCACTCAAA
GGCGGTAATACGGTTATCCACAGAATCAGGGGATAACGCAGGAAAGAACATGTGAGCAAAAGGC
CAGCAAAAGGCCAGGAACCGTAAAAAGGCCGCGTTGCTGGCGTTTTTCCATAGGCTCCGCCCCC
TGACGAGCATCACAAAAATCGACGCTCAAGTCAGAGGTGGCGAAACCCGACAGGACTATAAAG
ATACCAGGCGTTTCCCCCTGGAAGCTCCCTCGTGCGCTCTCCTGTTCCGACCCTGCCGCTTACCGG
ATACCTGTCCGCCTTTCTCCCTTCGGGAAGCGTGGCGCTTTCTCATAGCTCACGCTGTAGGTATCTC
AGTTCGGTGTAGGTCGTTCGCTCCAAGCTGGGCTGTGTGCACGAACCCCCCGTTCAGCCCGACCG

CTGCGCCTTATCCGGTAACTATCGTCTTGAGTCCAACCCGGTAAGACACGACTTATCGCCACTGGC
AGCAGCCACTGGTAACAGGATTAGCAGAGCGAGGTATGTAGGCGGTGCTACAGAGTTCTTGAAGT
GGTGGCCTAACTACGGCTACACTAGAAGAACAGTATTTGGTATCTGCGCTCTGCTGAAGCCAGTTA
CCTTCGGAAAAAGAGTTGGTAGCTCTTGATCCGGCAAACAAACCACCGCTGGTAGCGGTGGTTTT
TTTGTTTGCAAGCAGCAGATTACGCGCAGAAAAAAAGGATCTCAAGAAGATCCTTTGATCTTTTCT
ACGGGGTCTGACGCTCAGTGGAACGAAAACTCACGTTAAGGGATTTTGGTCATGAGATTATCAAA
AAGGATCTTCACCTAGATCCTTTTAAATTAAAAATGAAGTTTTAAATCAATCTAAAGTATATATGAG
TAAACTTGGTCTGACAGTTACCAATGCTTAATCAGTGAGGCACCTATCTCAGCGATCTGTCTATTTC
GTTCATCCATAGTTGCCTGACTCCCCGTCGTGTAGATAACTACGATACGGGAGGGCTTACCATCTG
GCCCCAGTGCTGCAATGATACCGCGAGACCCACGCTCACCGGCTCCAGATTTATCAGCAATAAAC
CAGCCAGCCGGAAGGGCCGAGCGCAGAAGTGGTCCTGCAACTTTATCCGCCTCCATCCAGTCTAT
TAATTGTTGCCGGGAAGCTAGAGTAAGTAGTTCGCCAGTTAATAGTTTGCGCAACGTTGTTGCCAT
TGCTACAGGCATCGTGGTGTCACGCTCGTCGTTTGGTATGGCTTCATTCAGCTCCGGTTCCCAACG
ATCAAGGCGAGTTACATGATCCCCCATGTTGTGCAAAAAAGCGGTTAGCTCCTTCGGTCCTCCGAT
CGTTGTCAGAAGTAAGTTGGCCGCAGTGTTATCACTCATGGTTATGGCAGCACTGCATAATTCTCTT
ACTGTCATGCCATCCGTAAGATGCTTTTCTGTGACTGGTGAGTACTCAACCAAGTCATTCTGAGAA
TAGTGTATGCGGCGACCGAGTTGCTCTTGCCCGGCGTCAATACGGGATAATACCGCGCCACATAGC
AGAACTTTAAAAGTGCTCATCATTGGAAAACGTTCTTCGGGGCGAAAACTCTCAAGGATCTTACC
GCTGTTGAGATCCAGTTCGATGTAACCCACTCGTGCACCCAACTGATCTTCAGCATCTTTTACTTTC
ACCAGCGTTTCTGGGTGAGCAAAAACAGGAAGGCAAAATGCCGCAAAAAAGGGAATAAGGGCG
ACACGGAAATGTTGAATACTCATACTCTTCCTTTTTCAATATTATTGAAGCATTTATCAGGGTTATTG
TCTCATGAGCGGATACATATTTGAATGTATTTAGAAAAATAAACAAATAGGGGTTCCGCGCACATT
TCCCCGAAAAGTGCCAC

Sequence S04. Sequence of the 4612 PB plasmid pBSK-HASA1. The sequence of the coding Sequence of *hasA1* gene is highlighted in yellow.

CTGACGCGCCCTGTAGCGGCGCATTAAGCGCGGCGGGTGTGGTGGTTACGCGCAGCGTGACCGCT
ACACTTGCCAGCGCCCTAGCGCCCGCTCCTTTCGCTTTCTTCCCTTCCTTTCTCGCCACGTTCGCCG
GCTTTCCCCGTCAAGCTCTAAATCGGGGGCTCCCTTTAGGGTTCCGATTTAGTGCTTTACGGCACCT
CGACCCCAAAAAACTTGATTAGGGTGATGGTTCACGTAGTGGGCCATCGCCCTGATAGACGGTTTT
TCGCCCTTTGACGTTGGAGTCCACGTTCTTTAATAGTGGACTCTTGTTCCAAACTGGAACAACACT
CAACCCTATCTCGGTCTATTCTTTTGATTTATAAGGGATTTTGCCGATTTCGGCCTATTGGTTAAAAA
ATGAGCTGATTTAACAAAAATTTAACGCGAATTTTAACAAAATATTAACGCTTACAATTTGCCATTC
GCCATTCAGGCTGCGCAACTGTTGGGAAGGGCGATCGGTGCGGGCCTCTTCGCTATTACGCCAGC
TGGCGAAAGGGGGATGTGCTGCAAGGCGATTAAGTTGGGTAACGCCAGGGTTTTCCCAGTCACGA
CGTTGTAAAACGACGGCCAGTGAATTGTAATACGACTCACTATAGGGCGACCCTTTAAGCTTATGA
GGCAACAAGATGCTCCTAAGCCTACTCCTGCAGCTTGCAGATGTAGTGGATTAGCCAGAAGGGTT
TTAACTATCGCATTCGCTTTGTTAATATTGGGTTTAATGACCTGGGCATATGCCGCCGGTGTGCCTTT
GGCAAGTGATAGATATGGATTGTTGGCCTTCGGTTTATATGGAGCCTTCTTGTCAGCTCACTTGGTT
GCTCAGTCATTGTTCGCTTACTTGGAACACAGGAGAGTTGCTGCTGCAGCTAGAGGTCCATTGGAT
GCAGCTACAGCTCGTTCAGTGGCTTTGACTATCTCTGCTTATCAGGAAGACCCAGCCTACTTGAGA
CAATGCTTAGCATCTGCAAGAGCATTGTTGTATCCACGTGCAAGGTTACGTGTGTTGATGGTTGTA

GATGGTAATAGAGCCGAAGATTTGTACATGGTAGATATGTTCCGTGAAGTGTTTGCAGATGAAGAT
CCAGCCACATACGTCTGGGACGGTAACTATCATCAACCTTGGGAACCAGCTGCAGCAGGAGCAGT
AGGTGCAGGAGCATATAGGGAGGTGGAAGCAGAGGATCCAGGTAGATTAGCTGTTGAAGCATTA
GTAAGAACAAGGAGATGTGTATGCGTGGCCCAAAGGTGGGGTGGTAAGAGGGAGGTAATGTACA
CTGCCTTTAAAGCCTTAGGAGATTCAGTTGACTATGTGCAGGTATGCGATTCAGACACCAGATTGG
ACCCTATGGCATTGTTGGAGTTAGTAAGAGTATTGGATGAGGATCCTAGAGTTGGTGCAGTGGGTG
GTGATGTGCGTATCTTGAACCCTTTGGATTCATGGGTCTCTTTCTTGTCTTCATTGAGATACTGGGTG
GCCTTTAATGTAGAACGTGCATGCCAGTCATACTTCCATTGTGTCTCATGCATCTCTGGACCTTTGG
GTTTATACAGAAACAACTTATTGCAACAGTTCTTAGAGGCATGGTACAACCAAAAGTTCTTAGGA
ACCCATTGCACCTTCGGAGATGACAGACACTTGACTAACAGAATGTTATCTATGGGTTATGCTACT
AAGTATACTAGTAGATCAAGGTGCTACTCAGAAACTCCTAGTTCATTCTTGAGATGGTTATCACAA
CAGACCAGGTGGTCAAAGAGTTACTTTAGAGAATGGTTGTACAATGCCTTGTGGTGGCACAGGCA
TCATGCTTGGATGACATACGAAGCAGTCGTTAGTGGATTGTTCCCTTTCTTTGTCGCAGCTACTGTA
TTACGTTTATTCTATGCAGGTAGACCATGGGCTTTATTGTGGGTATTATTATGTGTTCAAGGTGTAGC
TTTAGCAAAGGCCGCTTTTGCAGCATGGTTGAGAGGTTGCTTGAGAATGGTTTTATTGTCATTGTAC
GCTCCATTATACATGTGTGGATTGTTGCCAGCTAAGTTCTTAGCATTGGTAACAATGAATCAATCAG
GTTGGGGTACTTCAGGAAGAAGAAAATTAGCTGCTAATTACGTTCCTTTGTTGCCATTGGCTTTATG
GGCATTGTTGTTATTGGGTGGATTAGTAAGATCTGTAGCACATGAAGCCAGAGCAGATTGGTCAGG
TCCAAGTAGAGCAGCCGAGGCTTATCATTTGGCTGCAGGTGCAGGAGCCTACGTAGGTTACTGGG
TGGCTATGTTAACATTATACTGGGTTGGTGTTAGAAGATTGTGTAGAAGAAGAACTGGAGGTTACA
GAGTTCAAGTGTAAAGGCCTAAAGGGGATATCCTCGAGGTTCCCTTTAGTGAGGGTTAATTGCGA
GCTTGGCGTAATCATGGTCATAGCTGTTTCCTGTGTGAAATTGTTATCCGCTCACAATTCCACACAA
CATACGAGCCGGAAGCATAAAGTGTAAAGCCTGGGGTGCCTAATGAGTGAGCTAACTCACATTAA
TTGCGTTGCGCTCACTGCCCGCTTTCCAGTCGGGAAACCTGTCGTGCCAGCTGCATTAATGAATCG
GCCAACGCGCGGGGAGAGGCGGTTTGCGTATTGGGCGCTCTTCCGCTTCCTCGCTCACTGACTCG
CTGCGCTCGGTCGTTCGGCTGCGGCGAGCGGTATCAGCTCACTCAAAGGCGGTAATACGGTTATCC
ACAGAATCAGGGGATAACGCAGGAAAGAACATGTGAGCAAAAGGCCAGCAAAAGGCCAGGAA
CCGTAAAAAGGCCGCGTTGCTGGCGTTTTTCCATAGGCTCCGCCCCCCTGACGAGCATCACAAAA
ATCGACGCTCAAGTCAGAGGTGGCGAAACCCGACAGGACTATAAAGATACCAGGCGTTTCCCCCT
GGAAGCTCCCTCGTGCGCTCTCCTGTTCCGACCCTGCCGCTTACCGGATACCTGTCCGCCTTTCTCC
CTTCGGGAAGCGTGGCGCTTTCTCATAGCTCACGCTGTAGGTATCTCAGTTCGGTGTAGGTCGTTC
GCTCCAAGCTGGGCTGTGTGCACGAACCCCCCGTTCAGCCCGACCGCTGCGCCTTATCCGGTAAC
TATCGTCTTGAGTCCAACCCGGTAAGACACGACTTATCGCCACTGGCAGCAGCCACTGGTAACAG
GATTAGCAGAGCGAGGTATGTAGGCGGTGCTACAGAGTTCTTGAAGTGGTGGCCTAACTACGGCT
ACACTAGAAGAACAGTATTTGGTATCTGCGCTCTGCTGAAGCCAGTTACCTTCGGAAAAAGAGTT
GGTAGCTCTTGATCCGGCAAACAAACCACCGCTGGTAGCGGTGGTTTTTTTGTTTGCAAGCAGCA
GATTACGCGCAGAAAAAAAGGATCTCAAGAAGATCCTTTGATCTTTTCTACGGGGTCTGACGCTC
AGTGGAACGAAAACTCACGTTAAGGGATTTTGGTCATGAGATTATCAAAAAGGATCTTCACCTAG
ATCCTTTTAAATTAAAAATGAAGTTTTAAATCAATCTAAAGTATATATGAGTAAACTTGGTCTGACA
GTTACCAATGCTTAATCAGTGAGGCACCTATCTCAGCGATCTGTCTATTTCGTTCATCCATAGTTGC
CTGACTCCCCGTCGTGTAGATAACTACGATACGGGAGGGCTTACCATCTGGCCCCAGTGCTGCAAT
GATACCGCGAGACCCACGCTCACCGGCTCCAGATTTATCAGCAATAAACCAGCCAGCCGGAAGG
GCCGAGCGCAGAAGTGGTCCTGCAACTTTATCCGCCTCCATCCAGTCTATTAATTGTTGCCGGGAA
GCTAGAGTAAGTAGTTCGCCAGTTAATAGTTTGCGCAACGTTGTTGCCATTGCTACAGGCATCGTG

GTGTCACGCTCGTCGTTTGGTATGGCTTCATTCAGCTCCGGTTCCCAACGATCAAGGCGAGTTACA
TGATCCCCCATGTTGTGCAAAAAAGCGGTTAGCTCCTTCGGTCCTCCGATCGTTGTCAGAAGTAAG
TTGGCCGCAGTGTTATCACTCATGGTTATGGCAGCACTGCATAATTCTCTTACTGTCATGCCATCCG
TAAGATGCTTTTCTGTGACTGGTGAGTACTCAACCAAGTCATTCTGAGAATAGTGTATGCGGCGAC
CGAGTTGCTCTTGCCCGGCGTCAATACGGGATAATACCGCGCCACATAGCAGAACTTTAAAAGTG
CTCATCATTGGAAAACGTTCTTCGGGGCGAAAACTCTCAAGGATCTTACCGCTGTTGAGATCCAGT
TCGATGTAACCCACTCGTGCACCCAACTGATCTTCAGCATCTTTTACTTTCACCAGCGTTTCTGGGT
GAGCAAAAACAGGAAGGCAAAATGCCGCAAAAAAGGGAATAAGGGCGACACGGAAATGTTGA
ATACTCATACTCTTCCTTTTTCAATATTATTGAAGCATTTATCAGGGTTATTGTCTCATGAGCGGATA
CATATTTGAATGTATTTAGAAAAATAAACAAATAGGGGTTCCGCGCACATTTCCCCGAAAAGTGCC
AC

Sequence S05. Sequence of the 4540 PB plasmid pBSK-HASA2. The sequence of the coding Sequence of *hasA2* gene is highlighted in yellow.

CTGACGCGCCCTGTAGCGGCGCATTAAGCGCGGCGGGTGTGGTGGTTACGCGCAGCGTGACCGCT
ACACTTGCCAGCGCCCTAGCGCCCGCTCCTTTCGCTTTCTTCCCTTCCTTTCTCGCCACGTTCGCCG
GCTTTCCCCGTCAAGCTCTAAATCGGGGGCTCCCTTTAGGGTTCCGATTTAGTGCTTTACGGCACCT
CGACCCCAAAAAACTTGATTAGGGTGATGGTTCACGTAGTGGGCCATCGCCCTGATAGACGGTTTT
TCGCCCTTTGACGTTGGAGTCCACGTTCTTTAATAGTGGACTCTTGTTCCAAACTGGAACAACACT
CAACCCTATCTCGGTCTATTCTTTTGATTTATAAGGGATTTTGCCGATTTCGGCCTATTGGTTAAAAA
ATGAGCTGATTTAACAAAAATTTAACGCGAATTTTAACAAAATATTAACGCTTACAATTTGCCATTC
GCCATTCAGGCTGCGCAACTGTTGGGAAGGGCGATCGGTGCGGGCCTCTTCGCTATTACGCCAGC
TGGCGAAAGGGGGATGTGCTGCAAGGCGATTAAGTTGGGTAACGCCAGGGTTTTCCCAGTCACGA
CGTTGTAAAACGACGGCCAGTGAATTGTAATACGACTCACTATAGGGCGACCCTTAAAACTCGAG
ATGCACTGTGAAAGATTCTTGTGTATCTTGAGGATCATAGGTACTACCTTATTCGGTGTGTCATTGTT
GTTAGGTATTACAGCAGCTTACATAGTAGGTTATCAGTTCATACAGACTGACAATTACTACTTTAGT
TTTGGATTGTACGGAGCATTCTTGGCTTCTCATTTGATTATCCAGAGTTTGTTCGCCTTCTTAGAACA
TAGAAAGATGAAGAAATCATTAGAAACTCCAATCAAGTTGAATAAGACTGTTGCCTTGTGTATTGC
CGCATACCAAGAAGATCCAGACTACTTGAGAAAGTGCTTGCAATCAGTCAAGAGGTTAACCTATC
CAGGTATCAAAGTTGTGATGGTGATTGACGGAAATTCTGAAGATGATTTGTATATGATGGACATCTT
TAGTGAAGTCATGGGTAGAGATAAGTCTGCAACTTACATATGGAAGAATAACTTTCACGAAAAGG
GTCCAGGTGAAACTGATGAATCTCACAAAGAATCATCTCAACATGTAACTCAATTGGTCTTATCAA
ACAAATCAATTTGCATCATGCAGAAATGGGGTGGTAAGAGAGAAGTTATGTACACTGCTTTCAGA
GCATTAGGTCGTTCTGTTGATTATGTACAAGTCTGTGACAGTGATACAATGTTGGACCCAGCAAGT
TCAGTTGAGATGGTGAAAGTTTTAGAAGAAGATCCAATGGTGGGTGGTGTCGGTGGTGACGTTCA
GATTTTGAATAAGTACGACTCTTGGATCTCATTCTTGTCATCAGTAAGATACTGGATGGCCTTCAAC
ATTGAAAGAGCTTGTCAATCTTACTTCGGATGCGTGCAGTGTATTTCTGGTCCATTGGGTATGTATA
GAAATTCATTGTTACATGAGTTCGTGGAGGATTGGTACAATCAAGAGTTCATGGGTAACCAATGTT
CTTTCGGTGATGATAGACATTTGACAAATAGGGTTTTGTCTTTAGGTTATGCAACCAAGTACACCGC
TAGGTCAAAGTGTTTAACTGAAACTCCAATAGAATACTTGAGATGGTTGAATCAACAAACCAGAT
GGAGTAAGTCTTACTTCAGAGAATGGTTGTACAATGCTATGTGGTTTCACAAGCATCATTTGTGGAT
GACTTATGAGGCCATTATCACTGGTTTCTTTCCATTCTTCTTGATTGCAACTGTTATCCAGTTGTTCT

ACCGTGGTAAGATATGGAATATCTTGTTATTCTTATTGACTGTCCAATTGGTAGGATTGATAAAGTC
ATCATTTGCTTCTTGTTTGAGGGGTAACATTGTTATGGTGTTCATGTCTTTGTACAGTGTTTTGTATAT
GTCAAGTTTGTTGCCAGCTAAGATGTTCGCCATTGCTACAATCAACAAAGCTGGTTGGGGAACATC
TGGAAGAAAGACTATTGTTGTGAACTTCATTGGATTGATACCTGTGAGTGTGTGGTTTACCATCTTA
TTGGGTGGAGTTATCTTCACAATCTACAAGGAATCAAAGAGACCTTTCAGTGAATCTAAACAGAC
CGTGTTGATAGTAGGAACTTTGTTGTACGCTTGTTATTGGGTAATGTTGTTAACCTTGTACGTGGTCT
TGATCAATAAGTGTGGTAGACGTAAGAAGGGTCAACAATATGATATGGTCTTGGATGTTTGAGAAT
TCAAATTAGGGGATATCCTCGAGGTTCCCTTTAGTGAGGGTTAATTGCGAGCTTGGCGTAATCATG
GTCATAGCTGTTTCCTGTGTGAAATTGTTATCCGCTCACAATTCCACACAACATACGAGCCGGAAG
CATAAAGTGTAAAGCCTGGGGTGCCTAATGAGTGAGCTAACTCACATTAATTGCGTTGCGCTCACT
GCCCGCTTTCCAGTCGGGAAACCTGTCGTGCCAGCTGCATTAATGAATCGGCCAACGCGCGGGGA
GAGGCGGTTTGCGTATTGGGCGCTCTTCCGCTTCCTCGCTCACTGACTCGCTGCGCTCGGTCGTTC
GGCTGCGGCGAGCGGTATCAGCTCACTCAAAGGCGGTAATACGGTTATCCACAGAATCAGGGGAT
AACGCAGGAAAGAACATGTGAGCAAAAGGCCAGCAAAAGGCCAGGAACCGTAAAAAGGCCGC
GTTGCTGGCGTTTTTCCATAGGCTCCGCCCCCCTGACGAGCATCACAAAAATCGACGCTCAAGTC
AGAGGTGGCGAAACCCGACAGGACTATAAAGATACCAGGCGTTTCCCCCTGGAAGCTCCCTCGT
GCGCTCTCCTGTTCCGACCCTGCCGCTTACCGGATACCTGTCCGCCTTTCTCCCTTCGGGAAGCGT
GGCGCTTTCTCATAGCTCACGCTGTAGGTATCTCAGTTCGGTGTAGGTCGTTCGCTCCAAGCTGGG
CTGTGTGCACGAACCCCCCGTTCAGCCCGACCGCTGCGCCTTATCCGGTAACTATCGTCTTGAGTC
CAACCCGGTAAGACACGACTTATCGCCACTGGCAGCAGCCACTGGTAACAGGATTAGCAGAGCG
AGGTATGTAGGCGGTGCTACAGAGTTCTTGAAGTGGTGGCCTAACTACGGCTACACTAGAAGAAC
AGTATTTGGTATCTGCGCTCTGCTGAAGCCAGTTACCTTCGGAAAAAGAGTTGGTAGCTCTTGATC
CGGCAAACAAACCACCGCTGGTAGCGGTGGTTTTTTTGTTTGCAAGCAGCAGATTACGCGCAGAA
AAAAAGGATCTCAAGAAGATCCTTTGATCTTTTCTACGGGGTCTGACGCTCAGTGGAACGAAAAC
TCACGTTAAGGGATTTTGGTCATGAGATTATCAAAAAGGATCTTCACCTAGATCCTTTTAAATTAAA
AATGAAGTTTTAAATCAATCTAAAGTATATATGAGTAAACTTGGTCTGACAGTTACCAATGCTTAAT
CAGTGAGGCACCTATCTCAGCGATCTGTCTATTTCGTTCATCCATAGTTGCCTGACTCCCCGTCGTG
TAGATAACTACGATACGGGAGGGCTTACCATCTGGCCCCAGTGCTGCAATGATACCGCGAGACCC
ACGCTCACCGGCTCCAGATTTATCAGCAATAAACCAGCCAGCCGGAAGGGCCGAGCGCAGAAGT
GGTCCTGCAACTTTATCCGCCTCCATCCAGTCTATTAATTGTTGCCGGGAAGCTAGAGTAAGTAGTT
CGCCAGTTAATAGTTTGCGCAACGTTGTTGCCATTGCTACAGGCATCGTGGTGTCACGCTCGTCGT
TTGGTATGGCTTCATTCAGCTCCGGTTCCCAACGATCAAGGCGAGTTACATGATCCCCCATGTTGTG
CAAAAAAGCGGTTAGCTCCTTCGGTCCTCCGATCGTTGTCAGAAGTAAGTTGGCCGCAGTGTTATC
ACTCATGGTTATGGCAGCACTGCATAATTCTCTTACTGTCATGCCATCCGTAAGATGCTTTTCTGTG
ACTGGTGAGTACTCAACCAAGTCATTCTGAGAATAGTGTATGCGGCGACCGAGTTGCTCTTGCCCG
GCGTCAATACGGGATAATACCGCGCCACATAGCAGAACTTTAAAAGTGCTCATCATTGGAAAACG
TTCTTCGGGGCGAAAACTCTCAAGGATCTTACCGCTGTTGAGATCCAGTTCGATGTAACCCACTCG
TGCACCCAACTGATCTTCAGCATCTTTTACTTTCACCAGCGTTTCTGGGTGAGCAAAAACAGGAAG
GCAAAATGCCGCAAAAAAGGGAATAAGGGCGACACGGAAATGTTGAATACTCATACTCTTCCTTT
TTCAATATTATTGAAGCATTTATCAGGGTTATTGTCTCATGAGCGGATACATATTTGAATGTATTTAG
AAAAATAAACAAATAGGGGTTCCGCGCACATTTCCCCGAAAAGTGCCAC

Sequence S06. Sequence of the 4540 PB plasmid pBSK-HASA3. The sequence of the coding Sequence of *hasA3* gene is highlighted in yellow.

CTGACGCGCCCTGTAGCGGCGCATTAAGCGCGGCGGGTGTGGTGGTTACGCGCAGCGTGACCGCT
ACACTTGCCAGCGCCCTAGCGCCCGCTCCTTTCGCTTTCTTCCCTTCCTTTCTCGCCACGTTCGCCG
GCTTTCCCCGTCAAGCTCTAAATCGGGGGCTCCCTTTAGGGTTCCGATTTAGTGCTTTACGGCACCT
CGACCCCAAAAAACTTGATTAGGGTGATGGTTCACGTAGTGGGCCATCGCCCTGATAGACGGTTTT
TCGCCCTTTGACGTTGGAGTCCACGTTCTTTAATAGTGGACTCTTGTTCCAAACTGGAACAACACT
CAACCCTATCTCGGTCTATTCTTTTGATTTATAAGGGATTTTGCCGATTTCGGCCTATTGGTTAAAAA
ATGAGCTGATTTAACAAAAATTTAACGCGAATTTTAACAAAATATTAACGCTTACAATTTGCCATTC
GCCATTCAGGCTGCGCAACTGTTGGGAAGGGCGATCGGTGCGGGCCTCTTCGCTATTACGCCAGC
TGGCGAAAGGGGGATGTGCTGCAAGGCGATTAAGTTGGGTAACGCCAGGGTTTTCCCAGTCACGA
CGTTGTAAAACGACGGCCAGTGAATTGTAATACGACTCACTATAGGGCGACCCAAAACTCGAGAT
GCCAGTACAGTTGACTACAGCCTTGAGGGTTGTCGGAACATCATTGTTCGCTTTGGCTGTTTTAGG
TGGTATCTTGGCTGCTTATGTCACTGGTTATCAATTCATTCATACTGAAAAGCATTACTTAAGTTTCG
GATTGTATGGAGCTATCTTAGGTTTGCACTTGTTGATACAAAGTTTGTTCGCTTTCTTGGAGCATAG
AAGAATGCGTCGTGCTGGTCAGGCTTTGAAATTGCCTAGTCCTCGTAGAGGTAGTGTTGCTTTGTG
TATTGCAGCTTATCAGGAGGACCCTGATTACTTGAGAAAATGTTTAAGATCTGCTCAGAGAATTTC
ATTCCCTGATTTGAAGGTTGTTATGGTAGTGGATGGAAATCGTCAAGAAGACGCATACATGTTAGA
TATCTTTCATGAAGTATTGGGTGGTACTGAGCAGGCAGGTTTCTTCGTTTGGAGGTCAAACTTCCA
CGAAGCTGGTGAAGGAGAGACCGAAGCCTCATTGCAAGAAGGTATGGATAGAGTCAGGGACGTG
GTCAGAGCATCTACCTTCAGTTGCATTATGCAAAAGTGGGGTGGTAAGAGGGAGGTGATGTACAC
TGCATTCAAGGCTTTAGGTGATTCAGTGGACTATATCCAAGTGTGTGACTCAGATACAGTTTTAGAT
CCAGCCTGCACTATCGAGATGTTGAGAGTTTTGGAAGAAGATCCACAGGTCGGAGGTGTGGGTGG
AGATGTGCAGATATTGAACAAATACGATAGTTGGATCTCTTTCTTGTCTAGTGTAAGATACTGGATG
GCTTTCAACGTCGAAAGAGCTTGTCAATCTTACTTTGGTTGCGTTCAATGTATTTCTGGTCCTTTGG
GAATGTACAGAAATTCTTTGTTGCAACAGTTCTTGGAGGACTGGTACCATCAGAAATTCTTAGGAT
CTAAGTGTAGTTTTGGAGATGATAGACATTTGACTAATAGAGTGTTGAGTTTAGGTTACAGAACCA
AGTACACCGCTAGATCAAAGTGCTTGACTGAAACTCCAACCAAATACTTGAGATGGTTGAATCAA
CAAACAAGGTGGTCAAAGAGTTACTTCAGAGAATGGTTGTATAACTCTTTATGGTTTCACAAGCAT
CACTTATGGATGACTTATGAATCTGTTGTCACCGGTTTCTTTCCATTCTTCTTAATCGCTACAGTGAT
TCAGTTGTTTTACAGAGGTAGAATTTGGAATATTTTATTGTTCTTGTTGACTGTCCAATTAGTAGGTA
TCATCAAAGCTACTTACGCTTGTTTCTTACGTGGTAACGCCGAAATGATCTTCATGTCATTGTATAGT
TTGTTATACATGTCATCATTGTTGCCAGCCAAGATTTTCGCTATCGCCACCATCAATAAGTCAGGTT
GGGGTACTAGTGGAAGGAAAACAATTGTTGTCAACTTCATTGGATTGATACCAGTGAGTATTTGGG
TAGCAGTATTGTTAGGTGGTTTGGCCTATACTGCTTATTGTCAAGACTTGTTCTCAGAAACTGAATT
AGCATTCTTGGTTTCTGGTGCAATCTTATACGGTTGCTATTGGGTTGCTTTGTTAATGTTATACTTGGC
AATCATTGCTAGAAGATGCGGAAAGAAACCAGAACAGTACTCTTTAGCATTTGCCGAAGTTTAAG
AATTCAAATTGGGGATATCCTCGAGGTTCCCTTTAGTGAGGGTTAATTGCGAGCTTGGCGTAATCAT
GGTCATAGCTGTTTCCTGTGTGAAATTGTTATCCGCTCACAATTCCACACAACATACGAGCCGGAA
GCATAAAGTGTAAAGCCTGGGGTGCCTAATGAGTGAGCTAACTCACATTAATTGCGTTGCGCTCAC
TGCCCGCTTTCCAGTCGGGAAACCTGTCGTGCCAGCTGCATTAATGAATCGGCCAACGCGCGGGG
AGAGGCGGTTTGCGTATTGGGCGCTCTTCCGCTTCCTCGCTCACTGACTCGCTGCGCTCGGTCGTT
CGGCTGCGGCGAGCGGTATCAGCTCACTCAAAGGCGGTAATACGGTTATCCACAGAATCAGGGGA
TAACGCAGGAAAGAACATGTGAGCAAAAGGCCAGCAAAAGGCCAGGAACCGTAAAAAGGCCG

CGTTGCTGGCGTTTTTCCATAGGCTCCGCCCCCCTGACGAGCATCACAAAAATCGACGCTCAAGTC
AGAGGTGGCGAAACCCGACAGGACTATAAAGATACCAGGCGTTTCCCCCTGGAAGCTCCCTCGT
GCGCTCTCCTGTTCCGACCCTGCCGCTTACCGGATACCTGTCCGCCTTTCTCCCTTCGGGAAGCGT
GGCGCTTTCTCATAGCTCACGCTGTAGGTATCTCAGTTCGGTGTAGGTCGTTCGCTCCAAGCTGGG
CTGTGTGCACGAACCCCCCGTTCAGCCCGACCGCTGCGCCTTATCCGGTAACTATCGTCTTGAGTC
CAACCCGGTAAGACACGACTTATCGCCACTGGCAGCAGCCACTGGTAACAGGATTAGCAGAGCG
AGGTATGTAGGCGGTGCTACAGAGTTCTTGAAGTGGTGGCCTAACTACGGCTACACTAGAAGAAC
AGTATTTGGTATCTGCGCTCTGCTGAAGCCAGTTACCTTCGGAAAAAGAGTTGGTAGCTCTTGATC
CGGCAAACAAACCACCGCTGGTAGCGGTGGTTTTTTTGTTTGCAAGCAGCAGATTACGCGCAGAA
AAAAAGGATCTCAAGAAGATCCTTTGATCTTTTCTACGGGGTCTGACGCTCAGTGGAACGAAAAC
TCACGTTAAGGGATTTTGGTCATGAGATTATCAAAAAGGATCTTCACCTAGATCCTTTTAAATTAAA
AATGAAGTTTTAAATCAATCTAAAGTATATATGAGTAAACTTGGTCTGACAGTTACCAATGCTTAAT
CAGTGAGGCACCTATCTCAGCGATCTGTCTATTTCGTTCATCCATAGTTGCCTGACTCCCCGTCGTG
TAGATAACTACGATACGGGAGGGCTTACCATCTGGCCCCAGTGCTGCAATGATACCGCGAGACCC
ACGCTCACCGGCTCCAGATTTATCAGCAATAAACCAGCCAGCCGGAAGGGCCGAGCGCAGAAGT
GGTCCTGCAACTTTATCCGCCTCCATCCAGTCTATTAATTGTTGCCGGGAAGCTAGAGTAAGTAGTT
CGCCAGTTAATAGTTTGCGCAACGTTGTTGCCATTGCTACAGGCATCGTGGTGTCACGCTCGTCGT
TTGGTATGGCTTCATTCAGCTCCGGTTCCCAACGATCAAGGCGAGTTACATGATCCCCCATGTTGTG
CAAAAAAGCGGTTAGCTCCTTCGGTCCTCCGATCGTTGTCAGAAGTAAGTTGGCCGCAGTGTTATC
ACTCATGGTTATGGCAGCACTGCATAATTCTCTTACTGTCATGCCATCCGTAAGATGCTTTTCTGTG
ACTGGTGAGTACTCAACCAAGTCATTCTGAGAATAGTGTATGCGGCGACCGAGTTGCTCTTGCCCG
GCGTCAATACGGGATAATACCGCGCCACATAGCAGAACTTTAAAAGTGCTCATCATTGGAAAACG
TTCTTCGGGGCGAAAACTCTCAAGGATCTTACCGCTGTTGAGATCCAGTTCGATGTAACCCACTCG
TGCACCCAACTGATCTTCAGCATCTTTTACTTTCACCAGCGTTTCTGGGTGAGCAAAAACAGGAAG
GCAAAATGCCGCAAAAAAGGGAATAAGGGCGACACGGAAATGTTGAATACTCATACTCTTCCTTT
TTCAATATTATTGAAGCATTTATCAGGGTTATTGTCTCATGAGCGGATACATATTTGAATGTATTTAG
AAAAATAAACAAATAGGGGTTCCGCGCACATTTCCCCGAAAAGTGCCAC

Sequence S07. Sequence of the 11547 PB plasmid pkLAC2-B. The sequence of GPD Promoter and CYC1 terminator is highlighted in yellow and the coding Sequence of *hasB* gene is highlighted in gray.

AAGCTTGAAAAAAATGAAATTCTCTACTATATTAGCCGCATCTACTGCTTTAATTTCCGTTGTTATG
GCTGCTCCAGTTTCTACCGAAACTGACATCGACGATCTTCCAATATCGGTTCCAGAAGAAGCCTTG
ATTGGATTCATTGACTTAACCGGGGATGAAGTTTCCTTGTTGCCTGTTAATAACGGAACCCACACT
GGTATTCTATTCTTAAACACCACCATCGCTGAAGCTGCTTTCGCTGACAAGGATGATCTCGAGAAA
AGAGAGGCTGAAGCTAGAAGAGCTCATATGTCCATGGGCGGCCGCGATATCGTCGACGGATCCGA
ATTCCCTGCAGGTAATTAAATAAAGGCCTTGAATCGAGAATTTATACTTAGATAAGTATGTACTTAC
AGGTATATTTCTATGAGATACTGATGTATACATGCATGATAATATTTAAACGGTTATTAGTGCCGATT
GTCTTGTGCGATAATGACGTTCCTATCAAAGCAATACACTTACCACCTATTACATGGGCCAAGAAA
ATATTTTCGAACTTGTTTAGAATATTAGCACAGAGTATATGATGTTATCCGTTAGATTATGCATGATT
CATTCCTACAACTTTTTCGTAGCATAAGGATTAATTACTTGGATGCCAATAAAAAAAAAAAAACATC
GAGAAAATTTCAGCATGCTCAGAAACAATTGCAGTGTATCAAAGTAAAAAAAAGATTTTCACTAC
ATGTTCCTTTTGAAGAAAGAAAATCATGGAACATTAGATTTACAAAAATTTAACCACCGCTGATTA

ACGATTAGACCGTTAAGCGCACAACAGGTTATTAGTACAGAGAAAGCATTCTGTGGTGTTGCCCC
GGACTTTCTTTTGCGACATAGGTAAATCGAATACCATCATACTATCTTTTCCAATGACTCCCTAAAG
AAAGACTCTTCTTCGATGTTGTATACGTTGGAGCATAGGGCAAGAATTGTGGCTTGAGATCATCCT
TTTGTTGTTTCCGGGTGTACAATATGGACTTCCTCTTTTCTGGCAACCAAACCCATACATCGGGATT
CCTATAATACCTTCGTTGGTCTCCCTAACATGTAGGTGGCGGAGGGGAGATATACAATAGAACAGA
TACCAGACAAGACATAATGGGCTAAACAAGACTACACCAATTACACTGCCTCATTGATGGTGGTA
CATAACGAACTAATACTGTAGCCCTAGACTTGATAGCCATCATCATATCGAAGTTTCACTACCCTTT
TTCCATTTGCCATCTATTGAAGTAATAATAGGCGCATGCAACTTCTTTTCTTTTTTTTTCTTTTCTCTC
TCCCCCGTTGTTGTCTCACCATATCCGCAATGACAAAAAAATGATGGAAGACACTAAAGGAAAAA
ATTAACGACAAAGACAGCACCAACAGATGTCGTTGTTCCAGAGCTGATGAGGGGTATCTCGAAGC
ACACGAAACTTTTTCCTTCCTTCATTCACGCACACTACTCTCTAATGAGCAACGGTATACGGCCTTC
CTTCCAGTTACTTGAATTTGAAATAAAAAAAAGTTTGCTGTCTTGCTATCAAGTATAAATAGACCTG
CAATTATTAATCTTTTGTTTCCTCGTCATTGTTCTCGTTCCCTTTCTTCCTTGTTTCTTTTTCTGCACAA
TATTTCAAGCTATACCAAGCATACAATCAAGCAATTCCAGATCTGCCACCATGCCTCAATCCTGGG
AAGAACTGGCCGCTGATAAGCGCGCCCGCCTCGCAAAAACCATCCCTGATGAATGGAAAGTCCA
GACGCTGCCTGCGGAAGACAGCGTTATTGATTTCCCAAAGAAATCGGGCATCCTTTCAGAGGCCG
AACTGAAGATCACAGAGGCTTCCGCTGCGGATCTTGTGTCCAAGCTGGCGGCCGGAGAGTTGACC
TCGGTGGAAGTTACGCTAGCATTCTGTAAACGGGCAGCAATCGCCCAGCAGTTAACAAACTGCGC
CCACGAGTTCTTCCCTGACGCCGCTCTCGCGCAGGCAAGGGAACTCGATGAATACTACGCAAAGC
ACAAGAGACCCGTTGGTCCACTTCATGGCCTCCCCATCTCTCTCAAAGACCAGCTTCGAGTCAAG
GGCTACGAAACATCAATGGGCTACATCTCATGGCTAAACAAGTACGACGAAGGGGACTCGGTTCT
GACAACCATGCTCCGCAAAGCCGGTGCCGTCTTCTACGTCAAGACCTCTGTCCCGCAGACCCTGA
TGGTCTGCGAGACAGTCAACAACATCATCGGGCGCACCGTCAACCCACGCAACAAGAACTGGTC
GTGCGGCGGCAGTTCTGGTGGTGAGGGTGCGATCGTTGGGATTCGTGGTGGCGTCATCGGTGTAG
GAACGGACATCGGTGGCTCGATTCGAGTGCCGGCCGCGTTCAACTTCCTGTACGGTCTAAGGCCG
AGTCATGGGCGGCTGCCGTATGCAAAGATGGCGAACAGCATGGAGGGTCAGGAGACGGTGCACA
GCGTTGTCGGGCCGATTACGCACTCTGTTGAGGACCTCCGCCTCTTCACCAAATCCGTCCTCGGTC
AGGAGCCTTGGAAATACGACTCCAAGGTCATCCCCATGCCCTGGCGCCAGTCCGAGTCGGACATT
ATTGCCTCCAAGATCAAGAACGGCGGGCTCAATATCGGCTACTACAACTTCGACGGCAATGTCCTT
CCACACCCTCCTATCCTGCGCGGCGTGGAAACTACCGTCGCCGCACTCGCCAAAGCCGGTCACAC
CGTGACCCCGTGGACGCCATACAAGCACGATTTCGGCCACGATCTCATCTCCCATATCTACGCGGC
TGACGGCAGCGCCGACGTAATGCGCGACATCAGTGCATCCGGCGAGCCGGCGATTCCAAATATCA
AAGACCTACTGAACCCGAACATCAAAGCTGTTAACATGAACGAGCTCTGGGACACGCATCTCCA
GAAGTGGAATTACCAGATGGAGTACCTTGAGAAATGGCGGGAGGCTGAAGAAAAGGCCGGGAA
GGAACTGGACGCCATCATCGCGCCGATTACGCCTACCGCTGCGGTACGGCATGACCAGTTCCGGT
ACTATGGGTATGCCTCTGTGATCAACCTGCTGGATTTCACGAGCGTGGTTGTTCCGGTTACCTTTGC
GGATAAGAACATCGATAAGAAGAATGAGAGTTTCAAGGCGGTTAGTGAGCTTGATGCCCTCGTGC
AGGAAGAGTATGATCCGGAGGCGTATCATGGGGCACCGGTTGCAGTGCAGGTTATCGGACGGAG
ACTCAGTGAAGAGAGGACGTTGGCGATTGCAGAGGAAGTGGGGAAGTTGCTGGGAAATGTGGTG
ACTCCATAGCCCGGGGGGGGCTCGATCCCCTCGCGAGTTGGTTCAGCTGCTGCCTGAGGGTACCG
GCCGCAAATTAAAGCCTTCGAGCGTCCCAAAACCTTCTCAAGCAAGGTTTTCAGTATAATGTTACA
TGCGTACACGCGTCTGTACAGAAAAAAAAGAAAAATTTGAAATATAAATAACGTTCTTAATACTA
ACATAACTATAAAAAAATAAATAGGGACCTAGACTTCAGGTTGTCTAACTCCTTCCTTTTCGGTTAG
AGCGGATGTGGGGGGAGGGCGTGAATGTAAGCGTGACATAACTAATTACATGACTCGAGGTCGAC

TTACACACGTTGCTTCTTGTGTGGCAAGTCCTGTAAACCGAACTTAGGGATATCAGCAGTTGGAGT
GAATGGTATTCTTTTTGAAGCTACCTTCTTTCCGATGGTTTCCACCTGAAATCCAATGTTTTGCAATT
CTCCATGCAATTCATCTAAAACACGTCTACCATCGAATATGAAAGCAGGCTTTAACATCATCCTATG
GATTCTATTGAAATCTAATTCCTTGAACATATCCCATTCAGTACAAATGACCATAGCGTGTGCATTC
TCACAGGCTTCGTACAAATCTGTACTTATGTGGACCAATTGAGAAACCCTGTCGTCAGCTGCAACA
CCAGGTTGACTCAAGTCAGTGATGATCTGCTCACGTGGGACCTTTGGATCGTAGATATGTAACTTA
GCACCTTCATCCATCAAATACTTAGAGATATAGATTGAACTACTCTCTCTAGTATCACCTGTATCCTT
CTTGAAAGCGAAACCTAACAATGCGATTTTCTTATCGGTCACGGTGTTAAACAAACAATCGATTAT
CCTAGTTGTAAAACGTCTCCTTTGATAATCATTCATATCAATCACTTGTTGCCAGTACTTGGCCACTT
CGTGCAAGTTTAACACCTCACACAAGTAAACCAAGTTCAAAACGTCCTTCTGAAAACATGAACCT
CCAAATCCCACTGAAGCCTTCAAAAACTTGTTACCAATTCTTTGATCCATACCAATAGCTCTGGCA
ACCTCTTCAACGTCAGCTCCTGTGGCTTCACATAAGGCACTAATTGAGTTGATTGAAGAAATTCTT
TGAGCTAAGAATGCGTTGGCTGCTAACTTACTCAACTCAGAAGACCAGGTGTTTGTGGTTATGATT
TTCTCAGATGGTACCCAGTGTTCGTATACGTCACACAAAGCTCTAACAGCTTTCTGACCTTCAGGG
GTTTCGTCACCACCTATCAAAACTCTATCAGGGTTCTTCAAATCCTTAATGGCTGTACCCTCTGCCA
AAAACTCTGGGTTACTCAATACCTGCAAGTTCAAATCTGGTTTAGTATTTGCATCGAAGATACGTCT
TATTGATTCAGCAGCTCTAACTGGCACAGTAGATTTCTCTGTAACAATCTTGTATCCGTTACTATTCT
GTACTATTCTTCTAGCGCAAGCCTCAATGTATTTCAAGTCGGCTGCCCTTCCCTTACCCATACCGTA
AGTTTTTGTTGGAGTGTTGACTGAGATGAACACCAAATCAGCTTCTTGAATTGCACCATCAATGTC
AGTTGAGTAGAACAAATTCTTTCCCCTGCATGACTCTACGACTTCCTTCAAACCTGGTTCGTAGAT
AGGCAAAGTGTCACTATTCCAAGCATTGATCCTGGCTTGGTTCACATCCACAACAGTGACCTTAAT
GTCAGGGCACATCTGTGCAATGACAGAACAGGTTGGACCACCGACGTAACCGGCACCAATACAA
CAAATCTTCTTGATCTGAAACATTTTTGGATCCACTAGTTCTAGATCCGTCGAAACTAAGTTCTTGG
TGTTTTAAAACTAAAAAAAAGACTAACTATAAAAGTAGAATTTAAGAAGTTTAAGAAATAGATTTA
CAGAATTACAATCAATACCTACCGTCTTTATATACTTATTAGTCAAGTAGGGGAATAATTTCAGGGA
ACTGGTTTAAACCTTTTTTTTCAGCTTTTTCCAAATCAGAGAGAGCAGAAGGTAATAGAAGGTGTA
AGAAAATGAGATAGATACATGCGTGGGTCAATTGCCTTGTGTCATCATTTACTCCAGGCAGGTTGC
ATCACTCCATTGAGGTTGTGCCCGTTTTTTGCCTGTTTGTGCCCTGTTCTCTGTAGTTGCGCTAAGA
GAATGGACCTATGAACTGATGGTTGGTGAAGAAAACAATATTTTGGTGCTGGGATTCTTTTTTTTC
TGGATGCCAGCTTAAAAAGCGGGCTCCATTATATTTAGTGGATGCCAGGAATAAACCTGTTCACCC
AAGCACCATCAGTGTTATATATTCTGTGTAACCCGCCCCCTATTTTGGCATGTACGGGTTACAGCAG
AATTAAAAGGCTAATTTTTTGACTAAATAAAGTTAGGAAAATCACTACTATTAATTATTTACGTATTC
TTTGAAATGGCAGTATTGATAATGATAAACTCGAGCCTGAGGCTGGACGACCTCGCGGAGTTCTAC
CGGCAGTGCAAATCCGTCGGCATCCAGGAAACCAGCAGCGGCTATCCGCGCATCCATGCCCCCG
AACTGCAGGAGTGGGGAGGCACGATGGCCGCTTTGGTCGATCTAGATTACGTGGAAGAAAGGTA
GTAAAAGTAGTAGTATAAGTAGTAAAAAGAGGTAAAAAGAGAAAACCGGCTACATACTAGAGAA
GCACGTACACAAAAACTCATAGGCACTTCATCATACGACAGTTTCTTGATGCATTATAATAGTGTAT
TAGATATTTTCAGAAATATGCATAGAACCTCCTCTTGCCTTTACTTTTTATACATAGAACATTGGCAG
ATTTACTTACACTACTTTGTTTCTACGCCATTTCTTTTGTTTTCAACACTTAGACAAGTTGTTGAGAA
CCGGACTACTAAAAAGCAATGTTCCCACTGAAAATCATGTACCTGCAGCATAATAACCCCCTAATT
CTGCATCGATCCAGTATGTTTTTTTTTCTCTACTCATTTTTACCTGAAGATAGAGCTTCTAAAACAAA
AAAAATCAGTGATTACATGCATATTGTGTGTTCTAGTAACCAAAGGAAAGGAACAGATAGATAAA
ATTCCGAGACTGTCAAATTAGGTTTTTTTCTTTTTTTTTGGCGGGAGTCAGTGGGCCGAAATATGTT
CTTGGCCTAGAACTTAATCTGGTTTGATCATGCCAATACTTGCCTGAGTGCCCGACTTTTTGCCCAC

CCTCTTGCCTTCTGTCATCCTTCAAAACCCACCTGTTTTCCAGCCGTATCTTCGCTCGCATCTACAC
ATACTGTGCCATATCTTGTGTGTAGCCGGACGTGACTATGACCAAAAACAAACAAGGAGAACTGT
TCGCCGATTTGTAACACTCCTGCATCCATCCAAGTGGGTATGCGCTATGCAATGTTAAGCTAGGTC
AGGTCAGACCAGGTCCAAGGACAGCAACTTGACTGTATGCAACCTTTACCATCTTTGCACAGAAC
ATACTTGTAGCTAGCTAGTTACACTTATGGACCGAAAAGGCACCCCACCATGTCTGTCCGGCTTTA
GAGTACGGCCGCAGACCGCTGATTTGCCTTGCCAAGCAGTAGTCACAATGCATCGCATGAGCACA
CGGGCACGGGCACGGGCACAGGAACCATTGGCAAAAATACCAGATACACTATACCGACGTATATC
AAGCCCAAGTTTAAAATTCCTAAATTTCCGCGGCTACTTTTCAATTCCCTATAGTGAGTCGTATTAA
ATTCGTAATCATGTCATAGCTGTTTCCTGTGTGAAATTGTTATCCGCTCACAATTCCACACAACATA
CGAGCCGGAAGCATAAAGTGTAAAGCCTGGGGTGCCTAATGAGTGAGCTAACTCACATTAATTGC
GTTGCGCTCACTGCCCGCTTTCCAGTCGGGAAACCTGTCGTGCCAGCTGCATTAATGAATCGGCCA
ACGCGCGGGGAGAGGCGGTTTGCGTATTGGGCGCTCTTCCGCTTCCTCGCTCACTGACTCGCTGCG
CTCGGTCGTTCGGCTGCGGCGAGCGGTATCAGCTCACTCAAAGGCGGTAATACGGTTATCCACAG
AATCAGGGGATAACGCAGGAAAGAACATGTGAGCAAAAGGCCAGCAAAAGGCCAGGAACCGTA
AAAAGGCCGCGTTGCTGGCGTTTTTCCATAGGCTCCGCCCCCCTGACGAGCATCACAAAAATCGA
CGCTCAAGTCAGAGGTGGCGAAACCCGACAGGACTATAAAGATACCAGGCGTTTCCCCCTGGAA
GCTCCCTCGTGCGCTCTCCTGTTCCGACCCTGCCGCTTACCGGATACCTGTCCGCCTTTCTCCCTTC
GGGAAGCGTGGCGCTTTCTCATAGCTCACGCTGTAGGTATCTCAGTTCGGTGTAGGTCGTTCGCTC
CAAGCTGGGCTGTGTGCACGAACCCCCCGTTCAGCCCGACCGCTGCGCCTTATCCGGTAACTATC
GTCTTGAGTCCAACCCGGTAAGACACGACTTATCGCCACTGGCAGCAGCCACTGGTAACAGGATT
AGCAGAGCGAGGTATGTAGGCGGTGCTACAGAGTTCTTGAAGTGGTGGCCTAACTACGGCTACAC
TAGAAGAACAGTATTTGGTATCTGCGCTCTGCTGAAGCCAGTTACCTTCGGAAAAAGAGTTGGTA
GCTCTTGATCCGGCAAACAAACCACCGCTGGTAGCGGTGGTTTTTTTGTTTGCAAGCAGCAGATTA
CGCGCAGAAAAAAAGGATCTCAAGAAGATCCTTTGATCTTTTCTACGGGGTCTGACGCTCAGTGG
AACGAAAACTCACGTTAAGGGATTTTGGTCATGAGATTATCAAAAAGGATCTTCACCTAGATCCTT
TTAAATTAAAAATGAAGTTTTAAATCAATCTAAAGTATATATGAGTAAACTTGGTCTGACAGTTACC
AATGCTTAATCAGTGAGGCACCTATCTCAGCGATCTGTCTATTTCGTTCATCCATAGTTGCCTGACT
CCCCGTCGTGTAGATAACTACGATACGGGAGGGCTTACCATCTGGCCCCAGTGCTGCAATGATACC
GCGAGACCCACGCTCACCGGCTCCAGATTTATCAGCAATAAACCAGCCAGCCGGAAGGGCCGAG
CGCAGAAGTGGTCCTGCAACTTTATCCGCCTCCATCCAGTCTATTAATTGTTGCCGGGAAGCTAGA
GTAAGTAGTTCGCCAGTTAATAGTTTGCGCAACGTTGTTGCCATTGCTACAGGCATCGTGGTGTCA
CGCTCGTCGTTTGGTATGGCTTCATTCAGCTCCGGTTCCCAACGATCAAGGCGAGTTACATGATCC
CCCATGTTGTGCAAAAAAGCGGTTAGCTCCTTCGGTCCTCCGATCGTTGTCAGAAGTAAGTTGGCC
GCAGTGTTATCACTCATGGTTATGGCAGCACTGCATAATTCTCTTACTGTCATGCCATCCGTAAGAT
GCTTTTCTGTGACTGGTGAGTACTCAACCAAGTCATTCTGAGAATAGTGTATGCGGCGACCGAGTT
GCTCTTGCCCGGCGTCAATACGGGATAATACCGCGCCACATAGCAGAACTTTAAAAGTGCTCATC
ATTGGAAAACGTTCTTCGGGGCGAAAACTCTCAAGGATCTTACCGCTGTTGAGATCCAGTTCGATG
TAACCCACTCGTGCACCCAACTGATCTTCAGCATCTTTTACTTTCACCAGCGTTTCTGGGTGAGCA
AAAACAGGAAGGCAAAATGCCGCAAAAAAGGGAATAAGGGCGACACGGAAATGTTGAATACTC
ATACTCTTCCTTTTTCAATATTATTGAAGCATTTATCAGGGTTATTGTCTCATGAGCGGATACATATTT
GAATGTATTTAGAAAAATAAACAAATAGGGGTTCCGCGCACATTTCCCCGAAAAGTGCCACCTGA
CGCGCCCTGTAGCGGCGCATTAAGCGCGGCGGGTGTGGTGGTTACGCGCAGCGTGACCGCTACAC
TTGCCAGCGCCCTAGCGCCCGCTCCTTTCGCTTTCTTCCCTTCCTTTCTCGCCACGTTCGCCGGCTTT
CCCCGTCAAGCTCTAAATCGGGGGCTCCCTTTAGGGTTCCGATTTAGTGCTTTACGGCACCTCGAC

CCCAAAAAACTTGATTAGGGTGATGGTTCACGTAGTGGGCCATCGCCCTGATAGACGGTTTTTCGC
CCTTTGACGTTGGAGTCCACGTTCTTTAATAGTGGACTCTTGTTCCAAACTGGAACAACACTCAAC
CCTATCTCGGTCTATTCTTTTGATTTATAAGGGATTTTGCCGATTTCGGCCTATTGGTTAAAAAATGA
GCTGATTTAACAAAAATTTAACGCGAATTTTAACAAAATATTAACGCTTACAATTTCCATTCGCCAT
TCAGGCTGCGCAACTGTTGGGAAGGGCGATCGGTGCGGGCCTCTTCGCTATTACGCCAGCTGGCG
AAAGGGGGATGTGCTGCAAGGCGATTAAGTTGGGTAACGCCAGGGTTTTCCCAGTCACGACGTTG
TAAAACGACGGCCAGTGCCAAGCTCCCGCGGGGATCGACTCATAAAATAGTAACCTTCTAATGCG
TATCTATTGACTACCAACCATTAGTGTGGTTGCAGAAGGCGGAATTTTCCCTTCTTCGAATTTAGCT
TGCTTTTTCATTTTTTATTTTCCATTTTTCAGTTTTTGTTTGTGTCGAATTTAGCCAGTTGCTTCTCCAA
GATGAAAAAAACCCCTGCGCAGTTTCTGTGCTGCAAGATCCTAATCGACTTTTCCACCCCCCACA
AAAGTAAATGTTCTTTTGTTACATTCGCGTGGGTAGCTAGCTCCCCGAATCTTCAAAGGACTTAGG
GACTGCACTACATCAGAGTGTGTTCACCTGGTTTGCTGCCTGGTTTGAAAGAAAAGAGCAGGGAA
CTCGCGGGTTCCCGGCGAATAATCATGCGATAGTCCTTTGGCCTTCCAAGTCGCATGTAGAGTAGA
CAACAGACAGGGAGGGCAGGAAGGATCTTTCACTGAGATCCTGTATCTTGTTGGGTAAGTCGGAT
GAAAGGGGAATCGTATGAGATTGGAGAGGATGCGGAAGAGGTAACGCCTTTTGTTAACTTGTTTA
ATTATTATGGGGCAGGCGAGAGGGGGAGGAATGTATGTGTGTGAGGCGGGCGAGACGGAGCCAT
CCAGGCCAGGTAGAAATAGAGAAAGCCGAATGTTAGACAATATGGCAGCGTAGTAGAGTAGGTA
GGTAGGCAAGTACTGCTAGCAAAGAGGAGAAGGGTAAGCTCACTCTTCGCATTCCACACCGTTAG
TGTGTCAGTTTGAACAAAAAAACAATCATCATACCAATTGATGGACTGTGGACTGGCTTTTGGAA
CGGCTTTTCGGACTGCGATTATTCGTGAGGAATCAAGGTAGGAATTTGGTCATATTTACGGACAAC
AGTGGGTGATTCCCATATCGAGTAGGAAAACGAGATCATGGTATCCTCAGATATGTTGCGGAAATC
TGTTCACCGCAAAGTTCAGGGTGCTCTGGTGGGTTTCGGTTGGTCTTTGCTTTGCTTCTCCCTTGTC
TTGCATGTTAATAATAGCCTAGCCTGTGAGCCGAAACTTAGGGTAGGCTTAGTGTTGGAACGTACA
TATCTATCACGTTGACTTGGTTTAACCAGGCGACCTGGTAGCCAGCCATACCCACACACGTTTTTG
TATCTTCAGTATAGTTGTGAAAAGTGTAGCGGAAATTTGTGGTCCGAGCAACAGCGTCTTTTTCTA
GTAGTGCGGTCGGTTACTTGGTTGACATTGGTATTTGGACTTTGTTGCTACACCATTCACTACTTGA
AGTCGAGTGTGAAGGGTATGATTTCTAGTGGTGAACACCTTTAGTTACGTAATGTTTTCATTGCTGT
TTTACTTGAGATTTCGATTGAGAAAAAGGTATTTAATAGCTCGAATCAATGTGAGAACAGAGAGA
AGATGTTCTTCCCTAACTCGAAAGGTATATGAGGCTTGTGTTTCTTAGGAGAATTATTATTCTTTTGT
TATGTTGCGCTTGTAGTTGGAAAAGGTGAAGAGACAAAAGCTGGAATTGTGAGCGGATAACAAG
CTCAACACTTGAAATTTAGGAAAGAGCAGAATTTGGCAAAAAAAATAAAAAAAAAAATAAACAC
ACATACTCATCGAG

Sequence S08. Sequence of the 14140 PB plasmid pkLAC2-BP. The coding sequence of *hasAP* gene is highlighted in green, the GPD Promoter and CYC1 terminator is highlighted in yellow and the Coding Sequence of *hasB* gene is highlighted in grey.

AAGCTTATGAATACCTTATCTCAAGCCATCAAGGCATACAATTCAAATGACTATCAATTGGCTTTGA
AATTGTTCGAAAAGTCAGCAGAAATCTACGGACGTAAGATAGTAGAGTTTCAGATTACTAAGTGC
AAGGAGAAATTGTCTGCCCATCCAAGTGTCAATTCAGCTCATCCTTCAGTCAACTCAGCACATTTG
TCAGTAAACAAAGAGGAAAAGGTTAATGTGTGCGATTCTCCATTGGATATTGCTACCCAATTGTTG
TTGTCAAATGTCAAGAAATTAGTGTTATCTGACAGTGAGAAGAACACATTGAAGAATAAGTGGAA
GTTGTTAACTGAAAAGAAAAGTGAGAATGCTGAAGTTCGTGCTGTAGCATTGGTACCAAAGGATT

TCCCAAAGGATTTGGTGTTGGCACCATTACCTGACCATGTGAATGACTTTACTTGGTACAAGAAGA
GAAAGAAACGTTTAGGTATCAAACCAGAACATCAACACGTGGGTTTGTCAATAATTGTCACCACT
TTCAATCGTCCTGCAATCTTAAGTATAACTTTGGCATGCTTAGTTAATCAAAAGACTCACTATCCAT
TCGAGGTGATTGTCACAGATGATGGATCACAAGAAGATTTGTCTCCAATCATAAGACAATATGAA
AACAAATTGGATATCCGTTATGTCAGACAAAAGGACAATGGTTTCCAAGCTAGTGCTGCTAGGAA
TATGGGTTTGAGATTAGCAAAGTATGATTTCATTGGTTTGTTGGATTGCGATATGGCACCTAACCCA
TTATGGGTGCATTCATATGTCGCTGAATTGTTAGAAGATGATGATTTGACAATCATTGGACCAAGAA
AGTACATTGATACACAACATATCGACCCAAAGGACTTCTTAAACAATGCATCTTTGTTGGAATCAT
TGCCAGAAGTTAAGACCAATAACTCAGTGGCCGCAAAAGGTGAAGGTACCGTTTCATTGGATTGG
AGGTTGGAGCAATTCGAAAAGACTGAAAACTTAAGATTGTCAGACTCTCCTTTTAGATTCTTCGCA
GCTGGTAATGTTGCTTTCGCCAAGAAGTGGTTGAACAAATCTGGATTCTTTGATGAAGAGTTCAAC
CATTGGGGTGGTGAAGATGTTGAGTTTGGATATAGATTGTTTAGGTATGGTTCATTCTTCAAGACTA
TTGACGGTATCATGGCCTACCATCAAGAGCCACCTGGTAAGGAAAACGAAACAGATAGGGAAGC
TGGAAAGAACATCACATTGGATATTATGAGGGAGAAGGTACCATATATTTACAGGAAGTTGTTGCC
TATCGAAGATTCACACATCAATAGAGTCCCTTTGGTTTCTATCTATATCCCAGCTTACAACTGTGCC
AATTATATTCAACGTTGTGTTGATTCTGCCTTGAACCAGACAGTTGTAGATTTGGAAGTCTGTATTT
GCAATGATGGTTCTACAGATAATACTTTGGAAGTTATCAACAAGTTGTACGGTAACAATCCAAGAG
TCAGAATCATGAGTAAACCAAATGGTGGTATTGCTAGTGCTTCTAATGCAGCAGTGAGTTTTGCCA
AAGGATATTACATAGGTCAATTAGATTCAGATGACTATTTGGAGCCAGATGCCGTAGAGTTATGTTT
GAAAGAGTTCTTGAAAGACAAAACTTTGGCTTGTGTATATACAACAAACAGAAATGTCAATCCTG
ATGGTTCTTTGATAGCAAATGGTTACAACTGGCCAGAGTTTAGTAGGGAGAAGTTGACTACTGCAA
TGATTGCTCATCACTTCCGTATGTTCACTATCAGGGCATGGCATTTGACCGATGGTTTTAATGAGAA
GATTGAGAATGCTGTGGACTACGATATGTTCTTGAAGTTGAGTGAAGTTGGTAAGTTCAAGCACTT
AAACAAAATCTGCTATAACAGGGTATTGCATGGTGATAATACAAGTATTAAGAAGTTGGGTATCCA
AAAGAAGAACCATTTCGTGGTCGTCAACCAGAGTTTGAACAGGCAAGGAATCACTTACTACAATT
ACGACGAGTTCGATGACTTAGATGAGTCTAGGAAATACATCTTTAACAAAACAGCTGAGTACCAG
GAAGAAATTGACATCTTAAAGGACATTAAGATCATACAAAACAAGGACGCTAAAATAGCAGTATC
TATCTTCTACCCAAATACTTTGAATGGTTTGGTCAAGAAATTGAATAACATCATCGAGTACAACAA
GAACATATTCGTTATTGTCTTGCATGTGGACAAGAACCATTTGACCCCAGATATCAAGAAAGAGAT
ATTGGCTTTCTACCACAAGCATCAAGTGAATATTTTGTTGAATAACGATATCTCATACTACACATCA
AACCGTTTAATCAAGACCGAGGCACATTTATCAAACATTAATAAGTTGTCACAGTTGAACTTGAAT
TGTGAATATATCATATTCGACAATCATGACTCTTTGTTCGTGAAGAATGATTCTTATGCCTATATGAA
GAAGTACGATGTTGGTATGAATTTCTCAGCCTTAACTCATGATTGGATTGAAAAGATTAACGCACA
TCCACCATTCAAGAAGTTGATTAAGACATACTTTAACGATAATGACTTGAAATCTATGAACGTTAA
AGGAGCTAGTCAAGGAATGTTTATGACATATGCATTGGCTCACGAATTGTTGACTATTATCAAAGA
GGTTATCACTTCTTGCCAATCTATCGATTCTGTACCAGAATACAACACTGAGGACATATGGTTTCAA
TTTGCATTGTTGATCTTGGAAAAGAAAACTGGTCATGTCTTTAACAAGACAAGTACCTTGACATAC
ATGCCTTGGGAGAGGAAGTTGCAATGGACCAATGAACAAATTGAATCAGCTAAACGTGGAGAAA
ACATTCCAGTGAACAAGTTCATAATCAATTCAATCACATTGTAAAGGCCTTGAATCGAGAATTTAT
ACTTAGATAAGTATGTACTTACAGGTATATTTCTATGAGATACTGATGTATACATGCATGATAATATTT
AAACGGTTATTAGTGCCGATTGTCTTGTGCGATAATGACGTTCCTATCAAAGCAATACACTTACCAC
CTATTACATGGGCCAAGAAAATATTTTCGAACTTGTTTAGAATATTAGCACAGAGTATATGATGTTA
TCCGTTAGATTATGCATGATTCATTCCTACAACTTTTTCGTAGCATAAGGATTAATTACTTGGATGCC
AATAAAAAAAAAAAACATCGAGAAAATTTCAGCATGCTCAGAAACAATTGCAGTGTATCAAAGT

AAAAAAAAGATTTTCACTACATGTTCCTTTTGAAGAAAGAAAATCATGGAACATTAGATTTACAA
AAATTTAACCACCGCTGATTAACGATTAGACCGTTAAGCGCACAACAGGTTATTAGTACAGAGAA
AGCATTCTGTGGTGTTGCCCCGGACTTTCTTTTGCGACATAGGTAAATCGAATACCATCATACTATC
TTTTCCAATGACTCCCTAAAGAAAGACTCTTCTTCGATGTTGTATACGTTGGAGCATAGGGCAAGA
ATTGTGGCTTGAGATCATCCTTTTGTTGTTTCCGGGTGTACAATATGGACTTCCTCTTTTCTGGCAAC
CAAACCCATACATCGGGATTCCTATAATACCTTCGTTGGTCTCCCTAACATGTAGGTGGCGGAGGG
GAGATATACAATAGAACAGATACCAGACAAGACATAATGGGCTAAACAAGACTACACCAATTAC
ACTGCCTCATTGATGGTGGTACATAACGAACTAATACTGTAGCCCTAGACTTGATAGCCATCATCAT
ATCGAAGTTTCACTACCCTTTTTCCATTTGCCATCTATTGAAGTAATAATAGGCGCATGCAACTTCTT
TTCTTTTTTTTTCTTTTCTCTCTCCCCCGTTGTTGTCTCACCATATCCGCAATGACAAAAAAATGATG
GAAGACACTAAAGGAAAAAATTAACGACAAAGACAGCACCAACAGATGTCGTTGTTCCAGAGCT
GATGAGGGGTATCTCGAAGCACACGAAACTTTTTCCTTCCTTCATTCACGCACACTACTCTCTAAT
GAGCAACGGTATACGGCCTTCCTTCCAGTTACTTGAATTTGAAATAAAAAAAAGTTTGCTGTCTTG
CTATCAAGTATAAATAGACCTGCAATTATTAATCTTTTGTTTCCTCGTCATTGTTCTCGTTCCCTTTCT
TCCTTGTTTCTTTTTCTGCACAATATTTCAAGCTATACCAAGCATACAATCAAGCAATTCCAGATCT
GCCACCATGCCTCAATCCTGGGAAGAACTGGCCGCTGATAAGCGCGCCCGCCTCGCAAAAACCA
TCCCTGATGAATGGAAAGTCCAGACGCTGCCTGCGGAAGACAGCGTTATTGATTTCCCAAAGAAA
TCGGGCATCCTTTCAGAGGCCGAACTGAAGATCACAGAGGCTTCCGCTGCGGATCTTGTGTCCAA
GCTGGCGGCCGGAGAGTTGACCTCGGTGGAAGTTACGCTAGCATTCTGTAAACGGGCAGCAATCG
CCCAGCAGTTAACAAACTGCGCCCACGAGTTCTTCCCTGACGCCGCTCTCGCGCAGGCAAGGGA
ACTCGATGAATACTACGCAAAGCACAAGAGACCCGTTGGTCCACTTCATGGCCTCCCCATCTCTCT
CAAAGACCAGCTTCGAGTCAAGGGCTACGAAACATCAATGGGCTACATCTCATGGCTAAACAAGT
ACGACGAAGGGGACTCGGTTCTGACAACCATGCTCCGCAAAGCCGGTGCCGTCTTCTACGTCAAG
ACCTCTGTCCCGCAGACCCTGATGGTCTGCGAGACAGTCAACAACATCATCGGGCGCACCGTCAA
CCCACGCAACAAGAACTGGTCGTGCGGCGGCAGTTCTGGTGGTGAGGGTGCGATCGTTGGGATTC
GTGGTGGCGTCATCGGTGTAGGAACGGACATCGGTGGCTCGATTCGAGTGCCGGCCGCGTTCAAC
TTCCTGTACGGTCTAAGGCCGAGTCATGGGCGGCTGCCGTATGCAAAGATGGCGAACAGCATGGA
GGGTCAGGAGACGGTGCACAGCGTTGTCGGGCCGATTACGCACTCTGTTGAGGACCTCCGCCTCT
TCACCAAATCCGTCCTCGGTCAGGAGCCTTGGAAATACGACTCCAAGGTCATCCCCATGCCCTGG
CGCCAGTCCGAGTCGGACATTATTGCCTCCAAGATCAAGAACGGCGGGCTCAATATCGGCTACTA
CAACTTCGACGGCAATGTCCTTCCACACCCTCCTATCCTGCGCGGCGTGGAAACTACCGTCGCCG
CACTCGCCAAAGCCGGTCACACCGTGACCCCGTGGACGCCATACAAGCACGATTTCGGCCACGA
TCTCATCTCCCATATCTACGCGGCTGACGGCAGCGCCGACGTAATGCGCGACATCAGTGCATCCGG
CGAGCCGGCGATTCCAAATATCAAAGACCTACTGAACCCGAACATCAAAGCTGTTAACATGAACG
AGCTCTGGGACACGCATCTCCAGAAGTGGAATTACCAGATGGAGTACCTTGAGAAATGGCGGGA
GGCTGAAGAAAAGGCCGGGAAGGAACTGGACGCCATCATCGCGCCGATTACGCCTACCGCTGCG
GTACGGCATGACCAGTTCCGGTACTATGGGTATGCCTCTGTGATCAACCTGCTGGATTTCACGAGC
GTGGTTGTTCCGGTTACCTTTGCGGATAAGAACATCGATAAGAAGAATGAGAGTTTCAAGGCGGTT
AGTGAGCTTGATGCCCTCGTGCAGGAAGAGTATGATCCGGAGGCGTATCATGGGGCACCGGTTGC
AGTGCAGGTTATCGGACGGAGACTCAGTGAAGAGAGGACGTTGGCGATTGCAGAGGAAGTGGGG
AAGTTGCTGGGAAATGTGGTGACTCCATAGCCCGGGGGGGGCTCGATCCCCTCGCGAGTTGGTTC
AGCTGCTGCCTGAGGGTACCGGCCGCAAATTAAAGCCTTCGAGCGTCCCAAAACCTTCTCAAGCA
AGGTTTTCAGTATAATGTTACATGCGTACACGCGTCTGTACAGAAAAAAAAGAAAAATTTGAAAT
ATAAATAACGTTCTTAATACTAACATAACTATAAAAAAATAAATAGGGACCTAGACTTCAGGTTGT

CTAACTCCTTCCTTTTCGGTTAGAGCGGATGTGGGGGAGGGCGTGAATGTAAGCGTGACATAACT
AATTACATGACTCGAGGTCGACTTACACACGTTGCTTCTTGTGTGGCAAGTCCTGTAAACCGAACT
TAGGGATATCAGCAGTTGGAGTGAATGGTATTCTTTTTGAAGCTACCTTCTTTCCGATGGTTTCCAC
CTGAAATCCAATGTTTTGCAATTCTCCATGCAATTCATCTAAAACACGTCTACCATCGAATATGAAA
GCAGGCTTTAACATCATCCTATGGATTCTATTGAAATCTAATTCCTTGAACATATCCCATTCAGTACA
AATGACCATAGCGTGTGCATTCTCACAGGCTTCGTACAAATCTGTACTTATGTGGACCAATTGAGA
AACCCTGTCGTCAGCTGCAACACCAGGTTGACTCAAGTCAGTGATGATCTGCTCACGTGGGACCT
TTGGATCGTAGATATGTAACTTAGCACCTTCATCCATCAAATACTTAGAGATATAGATTGAACTACT
CTCTCTAGTATCACCTGTATCCTTCTTGAAAGCGAAACCTAACAATGCGATTTTCTTATCGGTCACG
GTGTTAAACAAACAATCGATTATCCTAGTTGTAAAACGTCTCCTTTGATAATCATTCATATCAATCA
CTTGTTGCCAGTACTTGGCCACTTCGTGCAAGTTTAACACCTCACACAAGTAAACCAAGTTCAAA
ACGTCCTTCTGAAAACATGAACCTCCAAATCCCACTGAAGCCTTCAAAAACTTGTTACCAATTCTT
TGATCCATACCAATAGCTCTGGCAACCTCTTCAACGTCAGCTCCTGTGGCTTCACATAAGGCACTA
ATTGAGTTGATTGAAGAAATTCTTTGAGCTAAGAATGCGTTGGCTGCTAACTTACTCAACTCAGAA
GACCAGGTGTTTGTGGTTATGATTTTCTCAGATGGTACCCAGTGTTCGTATACGTCACACAAAGCTC
TAACAGCTTTCTGACCTTCAGGGGTTTCGTCACCACCTATCAAAACTCTATCAGGGTTCTTCAAATC
CTTAATGGCTGTACCCTCTGCCAAAAACTCTGGGTTACTCAATACCTGCAAGTTCAAATCTGGTTTA
GTATTTGCATCGAAGATACGTCTTATTGATTCAGCAGCTCTAACTGGCACAGTAGATTTCTCTGTAA
CAATCTTGTATCCGTTACTATTCTGTACTATTCTTCTAGCGCAAGCCTCAATGTATTTCAAGTCGGCT
GCCCTTCCCTTACCCATACCGTAAGTTTTTGTTGGAGTGTTGACTGAGATGAACACCAAATCAGCT
TCTTGAATTGCACCATCAATGTCAGTTGAGTAGAACAAATTCTTTCCCCTGCATGACTCTACGACTT
CCTTCAAACCTGGTTCGTAGATAGGCAAAGTGTCACTATTCCAAGCATTGATCCTGGCTTGGTTCA
CATCCACAACAGTGACCTTAATGTCAGGGCACATCTGTGCAATGACAGAACAGGTTGGACCACCG
ACGTAACCGGCACCAATACAACAAATCTTCTTGATCTGAAACATTTTTGGATCCACTAGTTCTAGA
TCCGTCGAAACTAAGTTCTTGGTGTTTTAAAACTAAAAAAAAGACTAACTATAAAAGTAGAATTTA
AGAAGTTTAAGAAATAGATTTACAGAATTACAATCAATACCTACCGTCTTTATATACTTATTAGTCA
AGTAGGGGAATAATTTCAGGGAACTGGTTTAAACCTTTTTTTTCAGCTTTTTCCAAATCAGAGAGA
GCAGAAGGTAATAGAAGGTGTAAGAAAATGAGATAGATACATGCGTGGGTCAATTGCCTTGTGTC
ATCATTTACTCCAGGCAGGTTGCATCACTCCATTGAGGTTGTGCCCGTTTTTTGCCTGTTTGTGCCCT
GTTCTCTGTAGTTGCGCTAAGAGAATGGACCTATGAACTGATGGTTGGTGAAGAAAACAATATTTT
GGTGCTGGGATTCTTTTTTTTTCTGGATGCCAGCTTAAAAAGCGGGCTCCATTATATTTAGTGGATG
CCAGGAATAAACCTGTTCACCCAAGCACCATCAGTGTTATATATTCTGTGTAACCCGCCCCCTATTT
TGGCATGTACGGGTTACAGCAGAATTAAAAGGCTAATTTTTTGACTAAATAAAGTTAGGAAAATCA
CTACTATTAATTATTTACGTATTCTTTGAAATGGCAGTATTGATAATGATAAACTCGAGCCTGAGGCT
GGACGACCTCGCGGAGTTCTACCGGCAGTGCAAATCCGTCGGCATCCAGGAAACCAGCAGCGGC
TATCCGCGCATCCATGCCCCCGAACTGCAGGAGTGGGGAGGCACGATGGCCGCTTTGGTCGATCT
AGATTACGTGGAAGAAAGGTAGTAAAAGTAGTAGTATAAGTAGTAAAAAGAGGTAAAAAGAGAA
AACCGGCTACATACTAGAGAAGCACGTACACAAAAACTCATAGGCACTTCATCATACGACAGTTT
CTTGATGCATTATAATAGTGTATTAGATATTTTCAGAAATATGCATAGAACCTCCTCTTGCCTTTACTT
TTTATACATAGAACATTGGCAGATTTACTTACACTACTTTGTTTCTACGCCATTTCTTTTGTTTTCAAC
ACTTAGACAAGTTGTTGAGAACCGGACTACTAAAAAGCAATGTTCCCACTGAAAATCATGTACCT
GCAGCATAATAACCCCCTAATTCTGCATCGATCCAGTATGTTTTTTTTTCTCTACTCATTTTTACCTG
AAGATAGAGCTTCTAAAACAAAAAAAATCAGTGATTACATGCATATTGTGTGTTCTAGTAACCAAA
GGAAAGGAACAGATAGATAAAATTCCGAGACTGTCAAATTAGGTTTTTTTCTTTTTTTTTGGCGGG

AGTCAGTGGGCCGAAATATGTTCTTGGCCTAGAACTTAATCTGGTTTGATCATGCCAATACTTGCCT
GAGTGCCCGACTTTTTGCCCACCCTCTTGCCTTCTGTCATCCTTCAAAACCCACCTGTTTTCCAGCC
GTATCTTCGCTCGCATCTACACATACTGTGCCATATCTTGTGTGTAGCCGGACGTGACTATGACCAA
AAACAAACAAGGAGAACTGTTCGCCGATTTGTAACACTCCTGCATCCATCCAAGTGGGTATGCGC
TATGCAATGTTAAGCTAGGTCAGGTCAGACCAGGTCCAAGGACAGCAACTTGACTGTATGCAACC
TTTACCATCTTTGCACAGAACATACTTGTAGCTAGCTAGTTACACTTATGGACCGAAAAGGCACCC
CACCATGTCTGTCCGGCTTTAGAGTACGGCCGCAGACCGCTGATTTGCCTTGCCAAGCAGTAGTCA
CAATGCATCGCATGAGCACACGGGCACGGGCACGGGCACAGGAACCATTGGCAAAAATACCAG
ATACACTATACCGACGTATATCAAGCCCAAGTTTAAAATTCCTAAATTTCCGCGGCTACTTTTCAAT
TCCCTATAGTGAGTCGTATTAAATTCGTAATCATGTCATAGCTGTTTCCTGTGTGAAATTGTTATCCG
CTCACAATTCCACACAACATACGAGCCGGAAGCATAAAGTGTAAAGCCTGGGGTGCCTAATGAGT
GAGCTAACTCACATTAATTGCGTTGCGCTCACTGCCCGCTTTCCAGTCGGGAAACCTGTCGTGCCA
GCTGCATTAATGAATCGGCCAACGCGCGGGGAGAGGCGGTTTGCGTATTGGGCGCTCTTCCGCTTC
CTCGCTCACTGACTCGCTGCGCTCGGTCGTTCGGCTGCGGCGAGCGGTATCAGCTCACTCAAAGG
CGGTAATACGGTTATCCACAGAATCAGGGGATAACGCAGGAAAGAACATGTGAGCAAAAGGCCA
GCAAAAGGCCAGGAACCGTAAAAAGGCCGCGTTGCTGGCGTTTTTCCATAGGCTCCGCCCCCCTG
ACGAGCATCACAAAAATCGACGCTCAAGTCAGAGGTGGCGAAACCCGACAGGACTATAAAGATA
CCAGGCGTTTCCCCCTGGAAGCTCCCTCGTGCGCTCTCCTGTTCCGACCCTGCCGCTTACCGGATA
CCTGTCCGCCTTTCTCCCTTCGGGAAGCGTGGCGCTTTCTCATAGCTCACGCTGTAGGTATCTCAGT
TCGGTGTAGGTCGTTCGCTCCAAGCTGGGCTGTGTGCACGAACCCCCCGTTCAGCCCGACCGCTG
CGCCTTATCCGGTAACTATCGTCTTGAGTCCAACCCGGTAAGACACGACTTATCGCCACTGGCAGC
AGCCACTGGTAACAGGATTAGCAGAGCGAGGTATGTAGGCGGTGCTACAGAGTTCTTGAAGTGGT
GGCCTAACTACGGCTACACTAGAAGAACAGTATTTGGTATCTGCGCTCTGCTGAAGCCAGTTACCT
TCGGAAAAAGAGTTGGTAGCTCTTGATCCGGCAAACAAACCACCGCTGGTAGCGGTGGTTTTTTT
GTTTGCAAGCAGCAGATTACGCGCAGAAAAAAAGGATCTCAAGAAGATCCTTTGATCTTTTCTAC
GGGGTCTGACGCTCAGTGGAACGAAAACTCACGTTAAGGGATTTTGGTCATGAGATTATCAAAAA
GGATCTTCACCTAGATCCTTTTAAATTAAAAATGAAGTTTTAAATCAATCTAAAGTATATATGAGTA
AACTTGGTCTGACAGTTACCAATGCTTAATCAGTGAGGCACCTATCTCAGCGATCTGTCTATTTCGT
TCATCCATAGTTGCCTGACTCCCCGTCGTGTAGATAACTACGATACGGGAGGGCTTACCATCTGGC
CCCAGTGCTGCAATGATACCGCGAGACCCACGCTCACCGGCTCCAGATTTATCAGCAATAAACCA
GCCAGCCGGAAGGGCCGAGCGCAGAAGTGGTCCTGCAACTTTATCCGCCTCCATCCAGTCTATTA
ATTGTTGCCGGGAAGCTAGAGTAAGTAGTTCGCCAGTTAATAGTTTGCGCAACGTTGTTGCCATTG
CTACAGGCATCGTGGTGTCACGCTCGTCGTTTGGTATGGCTTCATTCAGCTCCGGTTCCCAACGATC
AAGGCGAGTTACATGATCCCCCATGTTGTGCAAAAAAGCGGTTAGCTCCTTCGGTCCTCCGATCGT
TGTCAGAAGTAAGTTGGCCGCAGTGTTATCACTCATGGTTATGGCAGCACTGCATAATTCTCTTACT
GTCATGCCATCCGTAAGATGCTTTTCTGTGACTGGTGAGTACTCAACCAAGTCATTCTGAGAATAG
TGTATGCGGCGACCGAGTTGCTCTTGCCCGGCGTCAATACGGGATAATACCGCGCCACATAGCAG
AACTTTAAAAGTGCTCATCATTGGAAAACGTTCTTCGGGGCGAAAACTCTCAAGGATCTTACCGCT
GTTGAGATCCAGTTCGATGTAACCCACTCGTGCACCCAACTGATCTTCAGCATCTTTTACTTTCACC
AGCGTTTCTGGGTGAGCAAAAACAGGAAGGCAAAATGCCGCAAAAAAGGGAATAAGGGCGACA
CGGAAATGTTGAATACTCATACTCTTCCTTTTTCAATATTATTGAAGCATTTATCAGGGTTATTGTCT
CATGAGCGGATACATATTTGAATGTATTTAGAAAAATAAACAAATAGGGGTTCCGCGCACATTTCC
CCGAAAAGTGCCACCTGACGCGCCCTGTAGCGGCGCATTAAGCGCGGCGGGTGTGGTGGTTACG
CGCAGCGTGACCGCTACACTTGCCAGCGCCCTAGCGCCCGCTCCTTTCGCTTTCTTCCCTTCCTTTC

TCGCCACGTTCGCCGGCTTTCCCCGTCAAGCTCTAAATCGGGGGCTCCCTTTAGGGTTCCGATTTA
GTGCTTTACGGCACCTCGACCCCAAAAAACTTGATTAGGGTGATGGTTCACGTAGTGGGCCATCG
CCCTGATAGACGGTTTTTCGCCCTTTGACGTTGGAGTCCACGTTCTTTAATAGTGGACTCTTGTTCC
AAACTGGAACAACACTCAACCCTATCTCGGTCTATTCTTTTGATTTATAAGGGATTTTGCCGATTTC
GGCCTATTGGTTAAAAAATGAGCTGATTTAACAAAAATTTAACGCGAATTTTAACAAAATATTAAC
GCTTACAATTTCCATTCGCCATTCAGGCTGCGCAACTGTTGGGAAGGGCGATCGGTGCGGGCCTCT
TCGCTATTACGCCAGCTGGCGAAAGGGGGATGTGCTGCAAGGCGATTAAGTTGGGTAACGCCAGG
GTTTTCCCAGTCACGACGTTGTAAAACGACGGCCAGTGCCAAGCTCCCGCGGGGATCGACTCATA
AAATAGTAACCTTCTAATGCGTATCTATTGACTACCAACCATTAGTGTGGTTGCAGAAGGCGGAAT
TTTCCCTTCTTCGAATTTAGCTTGCTTTTTCATTTTTTATTTCCATTTTTCAGTTTTTGTTTGTGTCGA
ATTTAGCCAGTTGCTTCTCCAAGATGAAAAAAACCCCTGCGCAGTTTCTGTGCTGCAAGATCCTAA
TCGACTTTTCCACCCCCCACAAAAGTAAATGTTCTTTTGTTACATTCGCGTGGGTAGCTAGCTCCCC
GAATCTTCAAAGGACTTAGGGACTGCACTACATCAGAGTGTGTTCACCTGGTTTGCTGCCTGGTTT
GAAAGAAAAGAGCAGGGAACTCGCGGGTTCCCGGCGAATAATCATGCGATAGTCCTTTGGCCTTC
CAAGTCGCATGTAGAGTAGACAACAGACAGGGAGGGCAGGAAGGATCTTTCACTGAGATCCTGT
ATCTTGTTGGGTAAGTCGGATGAAAGGGGAATCGTATGAGATTGGAGAGGATGCGGAAGAGGTAA
CGCCTTTTGTTAACTTGTTTAATTATTATGGGGCAGGCGAGAGGGGGAGGAATGTATGTGTGTGAG
GCGGGCGAGACGGAGCCATCCAGGCCAGGTAGAAATAGAGAAAGCCGAATGTTAGACAATATGG
CAGCGTAGTAGAGTAGGTAGGTAGGCAAGTACTGCTAGCAAAGAGGAGAAGGGTAAGCTCACTC
TTCGCATTCCACACCGTTAGTGTGTCAGTTTGAACAAAAAAACAATCATCATACCAATTGATGGAC
TGTGGACTGGCTTTTGGAACGGCTTTTCGGACTGCGATTATTCGTGAGGAATCAAGGTAGGAATTT
GGTCATATTTACGGACAACAGTGGGTGATTCCCATATCGAGTAGGAAAACGAGATCATGGTATCCT
CAGATATGTTGCGGAAATCTGTTCACCGCAAAGTTCAGGGTGCTCTGGTGGGTTTCGGTTGGTCTT
TGCTTTGCTTCTCCCTTGTCTTGCATGTTAATAATAGCCTAGCCTGTGAGCCGAAACTTAGGGTAGG
CTTAGTGTTGGAACGTACATATCTATCACGTTGACTTGGTTTAACCAGGCGACCTGGTAGCCAGCC
ATACCCACACACGTTTTTTGTATCTTCAGTATAGTTGTGAAAAGTGTAGCGGAAATTTGTGGTCCGA
GCAACAGCGTCTTTTTCTAGTAGTGCGGTCGGTTACTTGGTTGACATTGGTATTTGGACTTTGTTGC
TACACCATTCACTACTTGAAGTCGAGTGTGAAGGGTATGATTTCTAGTGGTGAACACCTTTAGTTA
CGTAATGTTTTCATTGCTGTTTTACTTGAGATTTCGATTGAGAAAAAGGTATTTAATAGCTCGAATC
AATGTGAGAACAGAGAGAAGATGTTCTTCCCTAACTCGAAAGGTATATGAGGCTTGTGTTTCTTAG
GAGAATTATTATTCTTTTGTTATGTTGCGCTTGTAGTTGGAAAAGGTGAAGAGACAAAAGCTGGAA
TTGTGAGCGGATAACAAGCTCAACACTTGAAATTTAGGAAAGAGCAGAATTTGGCAAAAAAAAT
AAAAAAAAAATAAACACACATACTCATCGAG

Sequence S09. Sequence of the 12937 PB plasmid pkLAC2-B1. The coding sequence of *hasA1* gene is highlighted in green and the coding sequence of *hasB* gene is highlighted in yellow.

AAGCTTATGAGGCAACAAGATGCTCCTAAGCCTACTCCTGCAGCTTGCAGATGTAGTGGATTAGCC
AGAAGGGTTTTAACTATCGCATTCGCTTTGTTAATATTGGGTTTAATGACCTGGGCATATGCCGCCG
GTGTGCCTTTGGCAAGTGATAGATATGGATTGTTGGCCTTCGGTTTATATGGAGCCTTCTTGTCAGC
TCACTTGGTTGCTCAGTCATTGTTCGCTTACTTGGAACACAGGAGAGTTGCTGCTGCAGCTAGAGG
TCCATTGGATGCAGCTACAGCTCGTTCAGTGGCTTTGACTATCTCTGCTTATCAGGAAGACCCAGC
CTACTTGAGACAATGCTTAGCATCTGCAAGAGCATTGTTGTATCCACGTGCAAGGTTACGTGTGTT
GATGGTTGTAGATGGTAATAGAGCCGAAGATTTGTACATGGTAGATATGTTCCGTGAAGTGTTTGC
AGATGAAGATCCAGCCACATACGTCTGGGACGGTAACTATCATCAACCTTGGGAACCAGCTGCAG

CAGGAGCAGTAGGTGCAGGAGCATATAGGGAGGTGGAAGCAGAGGATCCAGGTAGATTAGCTGT
TGAAGCATTAGTAAGAACAAGGAGATGTGTATGCGTGGCCCAAAGGTGGGGTGGTAAGAGGGAG
GTAATGTACACTGCCTTTAAAGCCTTAGGAGATTCAGTTGACTATGTGCAGGTATGCGATTCAGAC
ACCAGATTGGACCCTATGGCATTGTTGGAGTTAGTAAGAGTATTGGATGAGGATCCTAGAGTTGGT
GCAGTGGGTGGTGATGTGCGTATCTTGAACCCTTTGGATTCATGGGTCTCTTTCTTGTCTTCATTGA
GATACTGGGTGGCCTTTAATGTAGAACGTGCATGCCAGTCATACTTCCATTGTGTCTCATGCATCTC
TGGACCTTTGGGTTTATACAGAAACAACTTATTGCAACAGTTCTTAGAGGCATGGTACAACCAAA
AGTTCTTAGGAACCCATTGCACCTTCGGAGATGACAGACACTTGACTAACAGAATGTTATCTATGG
GTTATGCTACTAAGTATACTAGTAGATCAAGGTGCTACTCAGAAACTCCTAGTTCATTCTTGAGATG
GTTATCACAACAGACCAGGTGGTCAAAGAGTTACTTTAGAGAATGGTTGTACAATGCCTTGTGGTG
GCACAGGCATCATGCTTGGATGACATACGAAGCAGTCGTTAGTGGATTGTTCCCTTTCTTTGTCGC
AGCTACTGTATTACGTTTATTCTATGCAGGTAGACCATGGGCTTTATTGTGGGTATTATTATGTGTTCA
AGGTGTAGCTTTAGCAAAGGCCGCTTTTGCAGCATGGTTGAGAGGTTGCTTGAGAATGGTTTTATT
GTCATTGTACGCTCCATTATACATGTGTGGATTGTTGCCAGCTAAGTTCTTAGCATTGGTAACAATG
AATCAATCAGGTTGGGGTACTTCAGGAAGAAGAAAATTAGCTGCTAATTACGTTCCTTTGTTGCCA
TTGGCTTTATGGGCATTGTTGTTATTGGGTGGATTAGTAAGATCTGTAGCACATGAAGCCAGAGCA
GATTGGTCAGGTCCAAGTAGAGCAGCCGAGGCTTATCATTTGGCTGCAGGTGCAGGAGCCTACGT
AGGTTACTGGGTGGCTATGTTAACATTATACTGGGTTGGTGTTAGAAGATTGTGTAGAAGAAGAAC
TGGAGGTTACAGAGTTCAAGTGTAAAGGCCTTGAATCGAGAATTTATACTTAGATAAGTATGTACT
TACAGGTATATTTCTATGAGATACTGATGTATACATGCATGATAATATTTAAACGGTTATTAGTGCCG
ATTGTCTTGTGCGATAATGACGTTCCTATCAAAGCAATACACTTACCACCTATTACATGGGCCAAG
AAAATATTTTCGAACTTGTTTAGAATATTAGCACAGAGTATATGATGTTATCCGTTAGATTATGCATG
ATTCATTCCTACAACTTTTTCGTAGCATAAGGATTAATTACTTGGATGCCAATAAAAAAAAAAAAAC
ATCGAGAAAATTTCAGCATGCTCAGAAACAATTGCAGTGTATCAAAGTAAAAAAAAGATTTTCAC
TACATGTTCCTTTTGAAGAAAGAAAATCATGGAACATTAGATTTACAAAAATTTAACCACCGCTGA
TTAACGATTAGACCGTTAAGCGCACAACAGGTTATTAGTACAGAGAAAGCATTCTGTGGTGTTGCC
CCGGACTTTCTTTTGCGACATAGGTAAATCGAATACCATCATACTATCTTTTCCAATGACTCCCTAA
AGAAAGACTCTTCTTCGATGTTGTATACGTTGGAGCATAGGGCAAGAATTGTGGCTTGAGATCATC
CTTTTGTTGTTTCCGGGTGTACAATATGGACTTCCTCTTTTCTGGCAACCAAACCCATACATCGGGA
TTCCTATAATACCTTCGTTGGTCTCCCTAACATGTAGGTGGCGGAGGGGAGATATACAATAGAACA
GATACCAGACAAGACATAATGGGCTAAACAAGACTACACCAATTACACTGCCTCATTGATGGTGG
TACATAACGAACTAATACTGTAGCCCTAGACTTGATAGCCATCATCATATCGAAGTTTCACTACCCT
TTTTCCATTTGCCATCTATTGAAGTAATAATAGGCGCATGCAACTTCTTTTCTTTTTTTTTCTTTTCTCT
CTCCCCCGTTGTTGTCTCACCATATCCGCAATGACAAAAAAATGATGGAAGACACTAAAGGAAAA
AATTAACGACAAAGACAGCACCAACAGATGTCGTTGTTCCAGAGCTGATGAGGGGTATCTCGAA
GCACACGAAACTTTTTCCTTCCTTCATTCACGCACACTACTCTCTAATGAGCAACGGTATACGGCC
TTCCTTCCAGTTACTTGAATTTGAAATAAAAAAAAGTTTGCTGTCTTGCTATCAAGTATAAATAGAC
CTGCAATTATTAATCTTTTGTTTCCTCGTCATTGTTCTCGTTCCCTTTCTTCCTTGTTTCTTTTTCTGCA
CAATATTTCAAGCTATACCAAGCATACAATCAAGCAATTCCAGATCTGCCACCATGCCTCAATCCT
GGGAAGAACTGGCCGCTGATAAGCGCGCCCGCCTCGCAAAAACCATCCCTGATGAATGGAAAGT
CCAGACGCTGCCTGCGGAAGACAGCGTTATTGATTTCCCAAAGAAATCGGGCATCCTTTCAGAGG
CCGAACTGAAGATCACAGAGGCTTCCGCTGCGGATCTTGTGTCCAAGCTGGCGGCCGGAGAGTTG
ACCTCGGTGGAAGTTACGCTAGCATTCTGTAAACGGGCAGCAATCGCCCAGCAGTTAACAAACTG
CGCCCACGAGTTCTTCCCTGACGCCGCTCTCGCGCAGGCAAGGGAACTCGATGAATACTACGCAA

AGCACAAGAGACCCGTTGGTCCACTTCATGGCCTCCCCATCTCTCTCAAAGACCAGCTTCGAGTC
AAGGGCTACGAAACATCAATGGGCTACATCTCATGGCTAAACAAGTACGACGAAGGGGACTCGG
TTCTGACAACCATGCTCCGCAAAGCCGGTGCCGTCTTCTACGTCAAGACCTCTGTCCCGCAGACC
CTGATGGTCTGCGAGACAGTCAACAACATCATCGGGCGCACCGTCAACCCACGCAACAAGAACT
GGTCGTGCGGCGGCAGTTCTGGTGGTGAGGGTGCGATCGTTGGGATTCGTGGTGGCGTCATCGGT
GTAGGAACGGACATCGGTGGCTCGATTCGAGTGCCGGCCGCGTTCAACTTCCTGTACGGTCTAAG
GCCGAGTCATGGGCGGCTGCCGTATGCAAAGATGGCGAACAGCATGGAGGGTCAGGAGACGGTG
CACAGCGTTGTCGGGCCGATTACGCACTCTGTTGAGGACCTCCGCCTCTTCACCAAATCCGTCCTC
GGTCAGGAGCCTTGGAAATACGACTCCAAGGTCATCCCCATGCCCTGGCGCCAGTCCGAGTCGGA
CATTATTGCCTCCAAGATCAAGAACGGCGGGCTCAATATCGGCTACTACAACTTCGACGGCAATGT
CCTTCCACACCCTCCTATCCTGCGCGGCGTGGAAACTACCGTCGCCGCACTCGCCAAAGCCGGTC
ACACCGTGACCCCGTGGACGCCATACAAGCACGATTTCGGCCACGATCTCATCTCCCATATCTACG
CGGCTGACGGCAGCGCCGACGTAATGCGCGACATCAGTGCATCCGGCGAGCCGGCGATTCCAAA
TATCAAAGACCTACTGAACCCGAACATCAAAGCTGTTAACATGAACGAGCTCTGGGACACGCATC
TCCAGAAGTGGAATTACCAGATGGAGTACCTTGAGAAATGGCGGGAGGCTGAAGAAAAGGCCGG
GAAGGAACTGGACGCCATCATCGCGCCGATTACGCCTACCGCTGCGGTACGGCATGACCAGTTCC
GGTACTATGGGTATGCCTCTGTGATCAACCTGCTGGATTTCACGAGCGTGGTTGTTCCGGTTACCTT
TGCGGATAAGAACATCGATAAGAAGAATGAGAGTTTCAAGGCGGTTAGTGAGCTTGATGCCCTCG
TGCAGGAAGAGTATGATCCGGAGGCGTATCATGGGGCACCGGTTGCAGTGCAGGTTATCGGACGG
AGACTCAGTGAAGAGAGGACGTTGGCGATTGCAGAGGAAGTGGGGAAGTTGCTGGGAAATGTG
GTGACTCCATAGCCCGGGGGGGGCTCGATCCCCTCGCGAGTTGGTTCAGCTGCTGCCTGAGGGTA
CCGGCCGCAAATTAAAGCCTTCGAGCGTCCCAAAACCTTCTCAAGCAAGGTTTTCAGTATAATGTT
ACATGCGTACACGCGTCTGTACAGAAAAAAAAGAAAAATTTGAAATATAAATAACGTTCTTAATA
CTAACATAACTATAAAAAAATAAATAGGGACCTAGACTTCAGGTTGTCTAACTCCTTCCTTTTCGGT
TAGAGCGGATGTGGGGGGAGGGCGTGAATGTAAGCGTGACATAACTAATTACATGACTCGAGGTC
GACTTACACACGTTGCTTCTTGTGTGGCAAGTCCTGTAAACCGAACTTAGGGATATCAGCAGTTGG
AGTGAATGGTATTCTTTTTGAAGCTACCTTCTTTCCGATGGTTTCCACCTGAAATCCAATGTTTTGCA
ATTCTCCATGCAATTCATCTAAAACACGTCTACCATCGAATATGAAAGCAGGCTTTAACATCATCCT
ATGGATTCTATTGAAATCTAATTCCTTGAACATATCCCATTCAGTACAAATGACCATAGCGTGTGCA
TTCTCACAGGCTTCGTACAAATCTGTACTTATGTGGACCAATTGAGAAACCCTGTCGTCAGCTGCA
ACACCAGGTTGACTCAAGTCAGTGATGATCTGCTCACGTGGGACCTTTGGATCGTAGATATGTAAC
TTAGCACCTTCATCCATCAAATACTTAGAGATATAGATTGAACTACTCTCTCTAGTATCACCTGTATC
CTTCTTGAAAGCGAAACCTAACAATGCGATTTTCTTATCGGTCACGGTGTTAAACAAACAATCGAT
TATCCTAGTTGTAAAACGTCTCCTTTGATAATCATTCATATCAATCACTTGTTGCCAGTACTTGGCCA
CTTCGTGCAAGTTTAACACCTCACACAAGTAAACCAAGTTCAAAACGTCCTTCTGAAAACATGAA
CCTCCAAATCCCACTGAAGCCTTCAAAAACTTGTTACCAATTCTTTGATCCATACCAATAGCTCTG
GCAACCTCTTCAACGTCAGCTCCTGTGGCTTCACATAAGGCACTAATTGAGTTGATTGAAGAAATT
CTTTGAGCTAAGAATGCGTTGGCTGCTAACTTACTCAACTCAGAAGACCAGGTGTTTGTGGTTATG
ATTTTCTCAGATGGTACCCAGTGTTCGTATACGTCACACAAAGCTCTAACAGCTTTCTGACCTTCAG
GGGTTTCGTCACCACCTATCAAAACTCTATCAGGGTTCTTCAAATCCTTAATGGCTGTACCCTCTGC
CAAAAACTCTGGGTTACTCAATACCTGCAAGTTCAAATCTGGTTTAGTATTTGCATCGAAGATACG
TCTTATTGATTCAGCAGCTCTAACTGGCACAGTAGATTTCTCTGTAACAATCTTGTATCCGTTACTAT
TCTGTACTATTCTTCTAGCGCAAGCCTCAATGTATTTCAAGTCGGCTGCCCTTCCCTTACCCATACC
GTAAGTTTTTGTTGGAGTGTTGACTGAGATGAACACCAAATCAGCTTCTTGAATTGCACCATCAAT

GTCAGTTGAGTAGAACAAATTCTTTCCCCTGCATGACTCTACGACTTCCTTCAAACCTGGTTCGTA
GATAGGCAAAGTGTCACTATTCCAAGCATTGATCCTGGCTTGGTTCACATCCACAACAGTGACCTT
AATGTCAGGGCACATCTGTGCAATGACAGAACAGGTTGGACCACCGACGTAACCGGCACCAATA
CAACAAATCTTCTTGATCTGAAACATTTTTGGATCCACTAGTTCTAGATCCGTCGAAACTAAGTTCT
TGGTGTTTTAAAACTAAAAAAAAGACTAACTATAAAAGTAGAATTTAAGAAGTTTAAGAAATAGA
TTTACAGAATTACAATCAATACCTACCGTCTTTATATACTTATTAGTCAAGTAGGGGAATAATTTCAG
GGAACTGGTTTAAACCTTTTTTTTCAGCTTTTTCCAAATCAGAGAGAGCAGAAGGTAATAGAAGGT
GTAAGAAAATGAGATAGATACATGCGTGGGTCAATTGCCTTGTGTCATCATTTACTCCAGGCAGGT
TGCATCACTCCATTGAGGTTGTGCCCGTTTTTTGCCTGTTTGTGCCCTGTTCTCTGTAGTTGCGCTAA
GAGAATGGACCTATGAACTGATGGTTGGTGAAGAAAACAATATTTTGGTGCTGGGATTCTTTTTTTT
TCTGGATGCCAGCTTAAAAAGCGGGCTCCATTATATTTAGTGGATGCCAGGAATAAACCTGTTCAC
CCAAGCACCATCAGTGTTATATATTCTGTGTAACCCGCCCCCTATTTTGGCATGTACGGGTTACAGC
AGAATTAAAAGGCTAATTTTTTGACTAAATAAAGTTAGGAAAATCACTACTATTAATTATTTACGTA
TTCTTTGAAATGGCAGTATTGATAATGATAAACTCGAGCCTGAGGCTGGACGACCTCGCGGAGTTC
TACCGGCAGTGCAAATCCGTCGGCATCCAGGAAACCAGCAGCGGCTATCCGCGCATCCATGCCCC
CGAACTGCAGGAGTGGGGAGGCACGATGGCCGCTTTGGTCGATCTAGATTACGTGGAAGAAAGG
TAGTAAAAGTAGTAGTATAAGTAGTAAAAAGAGGTAAAAAGAGAAAACCGGCTACATACTAGAG
AAGCACGTACACAAAAACTCATAGGCACTTCATCATACGACAGTTTCTTGATGCATTATAATAGTG
TATTAGATATTTTCAGAAATATGCATAGAACCTCCTCTTGCCTTTACTTTTTATACATAGAACATTGG
CAGATTTACTTACACTACTTTGTTTCTACGCCATTTCTTTTGTTTTCAACACTTAGACAAGTTGTTGA
GAACCGGACTACTAAAAAGCAATGTTCCCACTGAAAATCATGTACCTGCAGCATAATAACCCCCT
AATTCTGCATCGATCCAGTATGTTTTTTTTTCTCTACTCATTTTTACCTGAAGATAGAGCTTCTAAAA
CAAAAAAAATCAGTGATTACATGCATATTGTGTGTTCTAGTAACCAAAGGAAAGGAACAGATAGA
TAAAATTCCGAGACTGTCAAATTAGGTTTTTTTCTTTTTTTTTGGCGGGAGTCAGTGGGCCGAAATA
TGTTCTTGGCCTAGAACTTAATCTGGTTTGATCATGCCAATACTTGCCTGAGTGCCCGACTTTTTGC
CCACCCTCTTGCCTTCTGTCATCCTTCAAAACCCACCTGTTTTCCAGCCGTATCTTCGCTCGCATCT
ACACATACTGTGCCATATCTTGTGTGTAGCCGGACGTGACTATGACCAAAAACAAACAAGGAGAA
CTGTTCGCCGATTTGTAACACTCCTGCATCCATCCAAGTGGGTATGCGCTATGCAATGTTAAGCTAG
GTCAGGTCAGACCAGGTCCAAGGACAGCAACTTGACTGTATGCAACCTTTACCATCTTTGCACAG
AACATACTTGTAGCTAGCTAGTTACACTTATGGACCGAAAAGGCACCCCACCATGTCTGTCCGGCT
TTAGAGTACGGCCGCAGACCGCTGATTTGCCTTGCCAAGCAGTAGTCACAATGCATCGCATGAGC
ACACGGGCACGGGCACGGGCACAGGAACCATTGGCAAAAATACCAGATACACTATACCGACGTA
TATCAAGCCCAAGTTTAAAATTCCTAAATTTCCGCGGCTACTTTTCAATTCCCTATAGTGAGTCGTA
TTAAATTCGTAATCATGTCATAGCTGTTTCCTGTGTGAAATTGTTATCCGCTCACAATTCCACACAA
CATACGAGCCGGAAGCATAAAGTGTAAAGCCTGGGGTGCCTAATGAGTGAGCTAACTCACATTAA
TTGCGTTGCGCTCACTGCCCGCTTTCCAGTCGGGAAACCTGTCGTGCCAGCTGCATTAATGAATCG
GCCAACGCGCGGGGAGAGGCGGTTTGCGTATTGGGCGCTCTTCCGCTTCCTCGCTCACTGACTCG
CTGCGCTCGGTCGTTCGGCTGCGGCGAGCGGTATCAGCTCACTCAAAGGCGGTAATACGGTTATCC
ACAGAATCAGGGGATAACGCAGGAAAGAACATGTGAGCAAAAGGCCAGCAAAAGGCCAGGAA
CCGTAAAAAGGCCGCGTTGCTGGCGTTTTTCCATAGGCTCCGCCCCCCTGACGAGCATCACAAAA
ATCGACGCTCAAGTCAGAGGTGGCGAAACCCGACAGGACTATAAAGATACCAGGCGTTTCCCCCT
GGAAGCTCCCTCGTGCGCTCTCCTGTTCCGACCCTGCCGCTTACCGGATACCTGTCCGCCTTTCTCC
CTTCGGGAAGCGTGGCGCTTTCTCATAGCTCACGCTGTAGGTATCTCAGTTCGGTGTAGGTCGTTC
GCTCCAAGCTGGGCTGTGTGCACGAACCCCCCGTTCAGCCCGACCGCTGCGCCTTATCCGGTAAC

TATCGTCTTGAGTCCAACCCGGTAAGACACGACTTATCGCCACTGGCAGCAGCCACTGGTAACAG
GATTAGCAGAGCGAGGTATGTAGGCGGTGCTACAGAGTTCTTGAAGTGGTGGCCTAACTACGGCT
ACACTAGAAGAACAGTATTTGGTATCTGCGCTCTGCTGAAGCCAGTTACCTTCGGAAAAAGAGTT
GGTAGCTCTTGATCCGGCAAACAAACCACCGCTGGTAGCGGTGGTTTTTTTGTTTGCAAGCAGCA
GATTACGCGCAGAAAAAAAGGATCTCAAGAAGATCCTTTGATCTTTTCTACGGGGTCTGACGCTC
AGTGGAACGAAAACTCACGTTAAGGGATTTTGGTCATGAGATTATCAAAAAGGATCTTCACCTAG
ATCCTTTTAAATTAAAAATGAAGTTTTAAATCAATCTAAAGTATATATGAGTAAACTTGGTCTGACA
GTTACCAATGCTTAATCAGTGAGGCACCTATCTCAGCGATCTGTCTATTTCGTTCATCCATAGTTGC
CTGACTCCCCGTCGTGTAGATAACTACGATACGGGAGGGCTTACCATCTGGCCCCAGTGCTGCAAT
GATACCGCGAGACCCACGCTCACCGGCTCCAGATTTATCAGCAATAAACCAGCCAGCCGGAAGG
GCCGAGCGCAGAAGTGGTCCTGCAACTTTATCCGCCTCCATCCAGTCTATTAATTGTTGCCGGGAA
GCTAGAGTAAGTAGTTCGCCAGTTAATAGTTTGCGCAACGTTGTTGCCATTGCTACAGGCATCGTG
GTGTCACGCTCGTCGTTTGGTATGGCTTCATTCAGCTCCGGTTCCCAACGATCAAGGCGAGTTACA
TGATCCCCCATGTTGTGCAAAAAAGCGGTTAGCTCCTTCGGTCCTCCGATCGTTGTCAGAAGTAAG
TTGGCCGCAGTGTTATCACTCATGGTTATGGCAGCACTGCATAATTCTCTTACTGTCATGCCATCCG
TAAGATGCTTTTCTGTGACTGGTGAGTACTCAACCAAGTCATTCTGAGAATAGTGTATGCGGCGAC
CGAGTTGCTCTTGCCCGGCGTCAATACGGGATAATACCGCGCCACATAGCAGAACTTTAAAAGTG
CTCATCATTGGAAAACGTTCTTCGGGGCGAAAACTCTCAAGGATCTTACCGCTGTTGAGATCCAGT
TCGATGTAACCCACTCGTGCACCCAACTGATCTTCAGCATCTTTTACTTTCACCAGCGTTTCTGGGT
GAGCAAAAACAGGAAGGCAAAATGCCGCAAAAAAGGGAATAAGGGCGACACGGAAATGTTGA
ATACTCATACTCTTCCTTTTTCAATATTATTGAAGCATTTATCAGGGTTATTGTCTCATGAGCGGATA
CATATTTGAATGTATTTAGAAAAATAAACAAATAGGGGTTCCGCGCACATTTCCCCGAAAAGTGCC
ACCTGACGCGCCCTGTAGCGGCGCATTAAGCGCGGCGGGTGTGGTGGTTACGCGCAGCGTGACCG
CTACACTTGCCAGCGCCCTAGCGCCCGCTCCTTTCGCTTTCTTCCCTTCCTTTCTCGCCACGTTCGC
CGGCTTTCCCCGTCAAGCTCTAAATCGGGGGCTCCCTTTAGGGTTCCGATTTAGTGCTTTACGGCAC
CTCGACCCCAAAAAACTTGATTAGGGTGATGGTTCACGTAGTGGGCCATCGCCCTGATAGACGGT
TTTTCGCCCTTTGACGTTGGAGTCCACGTTCTTTAATAGTGGACTCTTGTTCCAAACTGGAACAAC
ACTCAACCCTATCTCGGTCTATTCTTTTGATTTATAAGGGATTTTGCCGATTTCGGCCTATTGGTTAA
AAAATGAGCTGATTTAACAAAAATTTAACGCGAATTTTAACAAAATATTAACGCTTACAATTTCCA
TTCGCCATTCAGGCTGCGCAACTGTTGGGAAGGGCGATCGGTGCGGGCCTCTTCGCTATTACGCCA
GCTGGCGAAAGGGGGATGTGCTGCAAGGCGATTAAGTTGGGTAACGCCAGGGTTTTCCCAGTCAC
GACGTTGTAAAACGACGGCCAGTGCCAAGCTCCCGCGGGGATCGACTCATAAAATAGTAACCTTC
TAATGCGTATCTATTGACTACCAACCATTAGTGTGGTTGCAGAAGGCGGAATTTTCCCTTCTTCGAA
TTTAGCTTGCTTTTTCATTTTTTATTTTCCATTTTTCAGTTTTTGTTTGTGTCGAATTTAGCCAGTTGCT
TCTCCAAGATGAAAAAAACCCCTGCGCAGTTTCTGTGCTGCAAGATCCTAATCGACTTTTCCACCC
CCCACAAAAGTAAATGTTCTTTTGTTACATTCGCGTGGGTAGCTAGCTCCCCGAATCTTCAAAGGA
CTTAGGGACTGCACTACATCAGAGTGTGTTCACCTGGTTTGCTGCCTGGTTTGAAAGAAAAGAGC
AGGGAACTCGCGGGTTCCCGGCGAATAATCATGCGATAGTCCTTTGGCCTTCCAAGTCGCATGTAG
AGTAGACAACAGACAGGGAGGGCAGGAAGGATCTTTCACTGAGATCCTGTATCTTGTTGGGTAAG
TCGGATGAAAGGGGAATCGTATGAGATTGGAGAGGATGCGGAAGAGGTAACGCCTTTTGTTAACT
TGTTTAATTATTATGGGGCAGGCGAGAGGGGGAGGAATGTATGTGTGTGAGGCGGGCGAGACGGA
GCCATCCAGGCCAGGTAGAAATAGAGAAAGCCGAATGTTAGACAATATGGCAGCGTAGTAGAGT
AGGTAGGTAGGCAAGTACTGCTAGCAAAGAGGAGAAGGGTAAGCTCACTCTTCGCATTCCACAC
CGTTAGTGTGTCAGTTTGAACAAAAAAACAATCATCATACCAATTGATGGACTGTGGACTGGCTTT

TGGAACGGCTTTTCGGACTGCGATTATTCGTGAGGAATCAAGGTAGGAATTTGGTCATATTTACGG
ACAACAGTGGGTGATTCCCATATCGAGTAGGAAAACGAGATCATGGTATCCTCAGATATGTTGCGG
AAATCTGTTCACCGCAAAGTTCAGGGTGCTCTGGTGGGTTTCGGTTGGTCTTTGCTTTGCTTCTCCC
TTGTCTTGCATGTTAATAATAGCCTAGCCTGTGAGCCGAAACTTAGGGTAGGCTTAGTGTTGGAAC
GTACATATCTATCACGTTGACTTGGTTTAACCAGGCGACCTGGTAGCCAGCCATACCCACACACGT
TTTTTGTATCTTCAGTATAGTTGTGAAAAGTGTAGCGGAAATTTGTGGTCCGAGCAACAGCGTCTTT
TTCTAGTAGTGCGGTCGGTTACTTGGTTGACATTGGTATTTGGACTTTGTTGCTACACCATTCACTAC
TTGAAGTCGAGTGTGAAGGGTATGATTTCTAGTGGTGAACACCTTTAGTTACGTAATGTTTTCATTG
CTGTTTTACTTGAGATTTCGATTGAGAAAAAGGTATTTAATAGCTCGAATCAATGTGAGAACAGAG
AGAAGATGTTCTTCCCTAACTCGAAAGGTATATGAGGCTTGTGTTTCTTAGGAGAATTATTATTCTTT
TGTTATGTTGCGCTTGTAGTTGGAAAAGGTGAAGAGACAAAAGCTGGAATTGTGAGCGGATAACA
AGCTCAACACTTGAAATTTAGGAAAGAGCAGAATTTGGCAAAAAAAATAAAAAAAAAAATAAAC
ACACATACTCATCGAG

Sequence S10. Sequence of the 12863 PB plasmid pkLAC2-B2. The coding sequence of *hasA2* gene is highlighted in green and the coding Sequence of *hasB* gene is highlighted in yellow.

AAGCTTAAAAATGCACTGTGAAAGATTCTTGTGTATCTTGAGGATCATAGGTACTACCTTATTCGGT
GTGTCATTGTTGTTAGGTATTACAGCAGCTTACATAGTAGGTTATCAGTTCATACAGACTGACAATT
ACTACTTTAGTTTTGGATTGTACGGAGCATTCTTGGCTTCTCATTTGATTATCCAGAGTTTGTTCGCC
TTCTTAGAACATAGAAAGATGAAGAAATCATTAGAAACTCCAATCAAGTTGAATAAGACTGTTGC
CTTGTGTATTGCCGCATACCAAGAAGATCCAGACTACTTGAGAAAGTGCTTGCAATCAGTCAAGA
GGTTAACCTATCCAGGTATCAAAGTTGTGATGGTGATTGACGGAAATTCTGAAGATGATTTGTATAT
GATGGACATCTTTAGTGAAGTCATGGGTAGAGATAAGTCTGCAACTTACATATGGAAGAATAACTT
TCACGAAAAGGGTCCAGGTGAAACTGATGAATCTCACAAAGAATCATCTCAACATGTAACTCAAT
TGGTCTTATCAAACAAATCAATTTGCATCATGCAGAAATGGGGTGGTAAGAGAGAAGTTATGTAC
ACTGCTTTCAGAGCATTAGGTCGTTCTGTTGATTATGTACAAGTCTGTGACAGTGATACAATGTTGG
ACCCAGCAAGTTCAGTTGAGATGGTGAAAGTTTTAGAAGAAGATCCAATGGTGGGTGGTGTCGGT
GGTGACGTTCAGATTTTGAATAAGTACGACTCTTGGATCTCATTCTTGTCATCAGTAAGATACTGGA
TGGCCTTCAACATTGAAAGAGCTTGTCAATCTTACTTCGGATGCGTGCAGTGTATTTCTGGTCCATT
GGGTATGTATAGAAATTCATTGTTACATGAGTTCGTGGAGGATTGGTACAATCAAGAGTTCATGGG
TAACCAATGTTCTTTCGGTGATGATAGACATTTGACAAATAGGGTTTTGTCTTTAGGTTATGCAACC
AAGTACACCGCTAGGTCAAAGTGTTTAACTGAAACTCCAATAGAATACTTGAGATGGTTGAATCA
ACAAACCAGATGGAGTAAGTCTTACTTCAGAGAATGGTTGTACAATGCTATGTGGTTTCACAAGC
ATCATTTGTGGATGACTTATGAGGCCATTATCACTGGTTTCTTTCCATTCTTCTTGATTGCAACTGTT
ATCCAGTTGTTCTACCGTGGTAAGATATGGAATATCTTGTTATTCTTATTGACTGTCCAATTGGTAGG
ATTGATAAAGTCATCATTTGCTTCTTGTTTGAGGGGTAACATTGTTATGGTGTTCATGTCTTTGTACA
GTGTTTTGTATATGTCAAGTTTGTTGCCAGCTAAGATGTTCGCCATTGCTACAATCAACAAAGCTGG
TTGGGGAACATCTGGAAGAAAGACTATTGTTGTGAACTTCATTGGATTGATACCTGTGAGTGTGTG
GTTTACCATCTTATTGGGTGGAGTTATCTTCACAATCTACAAGGAATCAAAGAGACCTTTCAGTGA
ATCTAAACAGACCGTGTTGATAGTAGGAACTTTGTTGTACGCTTGTTATTGGGTAATGTTGTTAACC
TTGTACGTGGTCTTGATCAATAAGTGTGGTAGACGTAAGAAGGGTCAACAATATGATATGGTCTTG
GATGTTTGAAGGCCTTGAATCGAGAATTTATACTTAGATAAGTATGTACTTACAGGTATATTTCTATG

AGATACTGATGTATACATGCATGATAATATTTAAACGGTTATTAGTGCCGATTGTCTTGTGCGATAAT
GACGTTCCTATCAAAGCAATACACTTACCACCTATTACATGGGCCAAGAAAATATTTTCGAACTTG
TTTAGAATATTAGCACAGAGTATATGATGTTATCCGTTAGATTATGCATGATTCATTCCTACAACTTT
TTCGTAGCATAAGGATTAATTACTTGGATGCCAATAAAAAAAAAAAACATCGAGAAAATTTCAGC
ATGCTCAGAAACAATTGCAGTGTATCAAAGTAAAAAAAAGATTTTCACTACATGTTCCTTTTGAAG
AAAGAAAATCATGGAACATTAGATTTACAAAAATTTAACCACCGCTGATTAACGATTAGACCGTTA
AGCGCACAACAGGTTATTAGTACAGAGAAAGCATTCTGTGGTGTTGCCCCGGACTTTCTTTTGCGA
CATAGGTAAATCGAATACCATCATACTATCTTTTCCAATGACTCCCTAAAGAAAGACTCTTCTTCGA
TGTTGTATACGTTGGAGCATAGGGCAAGAATTGTGGCTTGAGATCATCCTTTTGTTGTTTCCGGGTG
TACAATATGGACTTCCTCTTTTCTGGCAACCAAACCCATACATCGGGATTCCTATAATACCTTCGTT
GGTCTCCCTAACATGTAGGTGGCGGAGGGGAGATATACAATAGAACAGATACCAGACAAGACATA
ATGGGCTAAACAAGACTACACCAATTACACTGCCTCATTGATGGTGGTACATAACGAACTAATACT
GTAGCCCTAGACTTGATAGCCATCATCATATCGAAGTTTCACTACCCTTTTTCCATTTGCCATCTATT
GAAGTAATAATAGGCGCATGCAACTTCTTTTCTTTTTTTTTCTTTTCTCTCTCCCCCGTTGTTGTCTCA
CCATATCCGCAATGACAAAAAAATGATGGAAGACACTAAAGGAAAAAATTAACGACAAAGACA
GCACCAACAGATGTCGTTGTTCCAGAGCTGATGAGGGGTATCTCGAAGCACACGAAACTTTTTCC
TTCCTTCATTCACGCACACTACTCTCTAATGAGCAACGGTATACGGCCTTCCTTCCAGTTACTTGAA
TTTGAAATAAAAAAAAAGTTTGCTGTCTTGCTATCAAGTATAAATAGACCTGCAATTATTAATCTTTT
GTTTCCTCGTCATTGTTCTCGTTCCCTTTCTTCCTTGTTTCTTTTTCTGCACAATATTTCAAGCTATAC
CAAGCATACAATCAAGCAATTCCAGATCTGCCACCATGCCTCAATCCTGGGAAGAACTGGCCGCT
GATAAGCGCGCCCGCCTCGCAAAAACCATCCCTGATGAATGGAAAGTCCAGACGCTGCCTGCGG
AAGACAGCGTTATTGATTTCCCAAAGAAATCGGGCATCCTTTCAGAGGCCGAACTGAAGATCACA
GAGGCTTCCGCTGCGGATCTTGTGTCCAAGCTGGCGGCCGGAGAGTTGACCTCGGTGGAAGTTAC
GCTAGCATTCTGTAAACGGGCAGCAATCGCCCAGCAGTTAACAAACTGCGCCCACGAGTTCTTCC
CTGACGCCGCTCTCGCGCAGGCAAGGGAACTCGATGAATACTACGCAAAGCACAAGAGACCCGT
TGGTCCACTTCATGGCCTCCCCATCTCTCTCAAAGACCAGCTTCGAGTCAAGGGCTACGAAACAT
CAATGGGCTACATCTCATGGCTAAACAAGTACGACGAAGGGGACTCGGTTCTGACAACCATGCTC
CGCAAAGCCGGTGCCGTCTTCTACGTCAAGACCTCTGTCCCGCAGACCCTGATGGTCTGCGAGAC
AGTCAACAACATCATCGGGCGCACCGTCAACCCACGCAACAAGAACTGGTCGTGCGGCGGCAGT
TCTGGTGGTGAGGGTGCGATCGTTGGGATTCGTGGTGGCGTCATCGGTGTAGGAACGGACATCGG
TGGCTCGATTCGAGTGCCGGCCGCGTTCAACTTCCTGTACGGTCTAAGGCCGAGTCATGGGCGGCT
GCCGTATGCAAAGATGGCGAACAGCATGGAGGGTCAGGAGACGGTGCACAGCGTTGTCGGGCCG
ATTACGCACTCTGTTGAGGACCTCCGCCTCTTCACCAAATCCGTCCTCGGTCAGGAGCCTTGGAAA
TACGACTCCAAGGTCATCCCCATGCCCTGGCGCCAGTCCGAGTCGGACATTATTGCCTCCAAGATC
AAGAACGGCGGGCTCAATATCGGCTACTACAACTTCGACGGCAATGTCCTTCCACACCCTCCTATC
CTGCGCGGCGTGGAAACTACCGTCGCCGCACTCGCCAAAGCCGGTCACACCGTGACCCCGTGGA
CGCCATACAAGCACGATTTCGGCCACGATCTCATCTCCCATATCTACGCGGCTGACGGCAGCGCCG
ACGTAATGCGCGACATCAGTGCATCCGGCGAGCCGGCGATTCCAAATATCAAAGACCTACTGAAC
CCGAACATCAAAGCTGTTAACATGAACGAGCTCTGGGACACGCATCTCCAGAAGTGGAATTACCA
GATGGAGTACCTTGAGAAATGGCGGGAGGCTGAAGAAAAGGCCGGGAAGGAACTGGACGCCAT
CATCGCGCCGATTACGCCTACCGCTGCGGTACGGCATGACCAGTTCCGGTACTATGGGTATGCCTC
TGTGATCAACCTGCTGGATTTCACGAGCGTGGTTGTTCCGGTTACCTTTGCGGATAAGAACATCGA
TAAGAAGAATGAGAGTTTCAAGGCGGTTAGTGAGCTTGATGCCCTCGTGCAGGAAGAGTATGATC
CGGAGGCGTATCATGGGGCACCGGTTGCAGTGCAGGTTATCGGACGGAGACTCAGTGAAGAGAG

GACGTTGGCGATTGCAGAGGAAGTGGGGAAGTTGCTGGGAAATGTGGTGACTCCATAGCCCGGG
GGGGGCTCGATCCCCTCGCGAGTTGGTTCAGCTGCTGCCTGAGGGTACCGGCCGCAAATTAAAGC
CTTCGAGCGTCCCAAAACCTTCTCAAGCAAGGTTTTCAGTATAATGTTACATGCGTACACGCGTCT
GTACAGAAAAAAAAGAAAAATTTGAAATATAAATAACGTTCTTAATACTAACATAACTATAAAAA
AATAAATAGGGACCTAGACTTCAGGTTGTCTAACTCCTTCCTTTTCGGTTAGAGCGGATGTGGGGG
GAGGGCGTGAATGTAAGCGTGACATAACTAATTACATGACTCGAGGTCGACTTACACACGTTGCTT
CTTGTGTGGCAAGTCCTGTAAACCGAACTTAGGGATATCAGCAGTTGGAGTGAATGGTATTCTTTT
TGAAGCTACCTTCTTTCCGATGGTTTCCACCTGAAATCCAATGTTTTGCAATTCTCCATGCAATTCA
TCTAAAACACGTCTACCATCGAATATGAAAGCAGGCTTTAACATCATCCTATGGATTCTATTGAAAT
CTAATTCCTTGAACATATCCCATTCAGTACAAATGACCATAGCGTGTGCATTCTCACAGGCTTCGTA
CAAATCTGTACTTATGTGGACCAATTGAGAAACCCTGTCGTCAGCTGCAACACCAGGTTGACTCA
AGTCAGTGATGATCTGCTCACGTGGGACCTTTGGATCGTAGATATGTAACTTAGCACCTTCATCCAT
CAAATACTTAGAGATATAGATTGAACTACTCTCTCTAGTATCACCTGTATCCTTCTTGAAAGCGAAA
CCTAACAATGCGATTTTCTTATCGGTCACGGTGTTAAACAAACAATCGATTATCCTAGTTGTAAAAC
GTCTCCTTTGATAATCATTCATATCAATCACTTGTTGCCAGTACTTGGCCACTTCGTGCAAGTTTAAC
ACCTCACACAAGTAAACCAAGTTCAAAACGTCCTTCTGAAAACATGAACCTCCAAATCCCACTG
AAGCCTTCAAAAACTTGTTACCAATTCTTTGATCCATACCAATAGCTCTGGCAACCTCTTCAACGT
CAGCTCCTGTGGCTTCACATAAGGCACTAATTGAGTTGATTGAAGAAATTCTTTGAGCTAAGAATG
CGTTGGCTGCTAACTTACTCAACTCAGAAGACCAGGTGTTTGTGGTTATGATTTTCTCAGATGGTAC
CCAGTGTTCGTATACGTCACACAAAGCTCTAACAGCTTTCTGACCTTCAGGGGTTTCGTCACCACC
TATCAAAACTCTATCAGGGTTCTTCAAATCCTTAATGGCTGTACCCTCTGCCAAAAACTCTGGGTTA
CTCAATACCTGCAAGTTCAAATCTGGTTTAGTATTTGCATCGAAGATACGTCTTATTGATTCAGCAG
CTCTAACTGGCACAGTAGATTTCTCTGTAACAATCTTGTATCCGTTACTATTCTGTACTATTCTTCTA
GCGCAAGCCTCAATGTATTTCAAGTCGGCTGCCCTTCCCTTACCCATACCGTAAGTTTTTGTTGGAG
TGTTGACTGAGATGAACACCAAATCAGCTTCTTGAATTGCACCATCAATGTCAGTTGAGTAGAAC
AAATTCTTTCCCCTGCATGACTCTACGACTTCCTTCAAACCTGGTTCGTAGATAGGCAAAGTGTCA
CTATTCCAAGCATTGATCCTGGCTTGGTTCACATCCACAACAGTGACCTTAATGTCAGGGCACATC
TGTGCAATGACAGAACAGGTTGGACCACCGACGTAACCGGCACCAATACAACAAATCTTCTTGAT
CTGAAACATTTTTGGATCCACTAGTTCTAGATCCGTCGAAACTAAGTTCTTGGTGTTTTAAAACTAA
AAAAAAGACTAACTATAAAAGTAGAATTTAAGAAGTTTAAGAAATAGATTTACAGAATTACAATC
AATACCTACCGTCTTTATATACTTATTAGTCAAGTAGGGGAATAATTTCAGGGAACTGGTTTAAACC
TTTTTTTTCAGCTTTTTCCAAATCAGAGAGAGCAGAAGGTAATAGAAGGTGTAAGAAAATGAGAT
AGATACATGCGTGGGTCAATTGCCTTGTGTCATCATTTACTCCAGGCAGGTTGCATCACTCCATTGA
GGTTGTGCCCGTTTTTTGCCTGTTTGTGCCCTGTTCTCTGTAGTTGCGCTAAGAGAATGGACCTATG
AACTGATGGTTGGTGAAGAAAACAATATTTTGGTGCTGGGATTCTTTTTTTTTCTGGATGCCAGCTT
AAAAAGCGGGCTCCATTATATTTAGTGGATGCCAGGAATAAACCTGTTCACCCAAGCACCATCAG
TGTTATATATTCTGTGTAACCCGCCCCCTATTTTGGCATGTACGGGTTACAGCAGAATTAAAAGGCT
AATTTTTTGACTAAATAAAGTTAGGAAAATCACTACTATTAATTATTTACGTATTCTTTGAAATGGCA
GTATTGATAATGATAAACTCGAGCCTGAGGCTGGACGACCTCGCGGAGTTCTACCGGCAGTGCAA
ATCCGTCGGCATCCAGGAAACCAGCAGCGGCTATCCGCGCATCCATGCCCCCGAACTGCAGGAGT
GGGGAGGCACGATGGCCGCTTTGGTCGATCTAGATTACGTGGAAGAAAGGTAGTAAAAGTAGTAG
TATAAGTAGTAAAAAGAGGTAAAAAGAGAAAACCGGCTACATACTAGAGAAGCACGTACACAAA
AACTCATAGGCACTTCATCATACGACAGTTTCTTGATGCATTATAATAGTGTATTAGATATTTTCAGA
AATATGCATAGAACCTCCTCTTGCCTTTACTTTTTATACATAGAACATTGGCAGATTTACTTACACTA

CTTTGTTTCTACGCCATTTCTTTTGTTTTCAACACTTAGACAAGTTGTTGAGAACCGGACTACTAAA
AAGCAATGTTCCCACTGAAAATCATGTACCTGCAGCATAATAACCCCCTAATTCTGCATCGATCCA
GTATGTTTTTTTTTCTCTACTCATTTTTACCTGAAGATAGAGCTTCTAAAACAAAAAAAATCAGTGA
TTACATGCATATTGTGTGTTCTAGTAACCAAAGGAAAGGAACAGATAGATAAAATTCCGAGACTGT
CAAATTAGGTTTTTTTCTTTTTTTTTGGCGGGAGTCAGTGGGCCGAAATATGTTCTTGGCCTAGAAC
TTAATCTGGTTTGATCATGCCAATACTTGCCTGAGTGCCCGACTTTTTGCCCACCCTCTTGCCTTCTG
TCATCCTTCAAAACCCACCTGTTTTCCAGCCGTATCTTCGCTCGCATCTACACATACTGTGCCATAT
CTTGTGTGTAGCCGGACGTGACTATGACCAAAAACAAACAAGGAGAACTGTTCGCCGATTTGTAA
CACTCCTGCATCCATCCAAGTGGGTATGCGCTATGCAATGTTAAGCTAGGTCAGGTCAGACCAGGT
CCAAGGACAGCAACTTGACTGTATGCAACCTTTACCATCTTTGCACAGAACATACTTGTAGCTAGC
TAGTTACACTTATGGACCGAAAAGGCACCCCACCATGTCTGTCCGGCTTTAGAGTACGGCCGCAG
ACCGCTGATTTGCCTTGCCAAGCAGTAGTCACAATGCATCGCATGAGCACACGGGCACGGGCACG
GGCACAGGAACCATTGGCAAAAATACCAGATACACTATACCGACGTATATCAAGCCCAAGTTTAA
AATTCCTAAATTTCCGCGGCTACTTTTCAATTCCCTATAGTGAGTCGTATTAAATTCGTAATCATGTC
ATAGCTGTTTCCTGTGTGAAATTGTTATCCGCTCACAATTCCACACAACATACGAGCCGGAAGCAT
AAAGTGTAAAGCCTGGGGTGCCTAATGAGTGAGCTAACTCACATTAATTGCGTTGCGCTCACTGCC
CGCTTTCCAGTCGGGAAACCTGTCGTGCCAGCTGCATTAATGAATCGGCCAACGCGCGGGGAGAG
GCGGTTTGCGTATTGGGCGCTCTTCCGCTTCCTCGCTCACTGACTCGCTGCGCTCGGTCGTTCGGCT
GCGGCGAGCGGTATCAGCTCACTCAAAGGCGGTAATACGGTTATCCACAGAATCAGGGGATAACG
CAGGAAAGAACATGTGAGCAAAAGGCCAGCAAAAGGCCAGGAACCGTAAAAAGGCCGCGTTGC
TGGCGTTTTTCCATAGGCTCCGCCCCCCTGACGAGCATCACAAAAATCGACGCTCAAGTCAGAGG
TGGCGAAACCCGACAGGACTATAAAGATACCAGGCGTTTCCCCCTGGAAGCTCCCTCGTGCGCTC
TCCTGTTCCGACCCTGCCGCTTACCGGATACCTGTCCGCCTTTCTCCCTTCGGGAAGCGTGGCGCTT
TCTCATAGCTCACGCTGTAGGTATCTCAGTTCGGTGTAGGTCGTTCGCTCCAAGCTGGGCTGTGTGC
ACGAACCCCCCGTTCAGCCCGACCGCTGCGCCTTATCCGGTAACTATCGTCTTGAGTCCAACCCGG
TAAGACACGACTTATCGCCACTGGCAGCAGCCACTGGTAACAGGATTAGCAGAGCGAGGTATGTA
GGCGGTGCTACAGAGTTCTTGAAGTGGTGGCCTAACTACGGCTACACTAGAAGAACAGTATTTGG
TATCTGCGCTCTGCTGAAGCCAGTTACCTTCGGAAAAAGAGTTGGTAGCTCTTGATCCGGCAAAC
AAACCACCGCTGGTAGCGGTGGTTTTTTTGTTTGCAAGCAGCAGATTACGCGCAGAAAAAAAGGA
TCTCAAGAAGATCCTTTGATCTTTTCTACGGGGTCTGACGCTCAGTGGAACGAAAACTCACGTTAA
GGGATTTTGGTCATGAGATTATCAAAAAGGATCTTCACCTAGATCCTTTTAAATTAAAAATGAAGTT
TTAAATCAATCTAAAGTATATATGAGTAAACTTGGTCTGACAGTTACCAATGCTTAATCAGTGAGGC
ACCTATCTCAGCGATCTGTCTATTTCGTTCATCCATAGTTGCCTGACTCCCCGTCGTGTAGATAACTA
CGATACGGGAGGGCTTACCATCTGGCCCCAGTGCTGCAATGATACCGCGAGACCCACGCTCACCG
GCTCCAGATTTATCAGCAATAAACCAGCCAGCCGGAAGGGCCGAGCGCAGAAGTGGTCCTGCAA
CTTTATCCGCCTCCATCCAGTCTATTAATTGTTGCCGGGAAGCTAGAGTAAGTAGTTCGCCAGTTAA
TAGTTTGCGCAACGTTGTTGCCATTGCTACAGGCATCGTGGTGTCACGCTCGTCGTTTGGTATGGCT
TCATTCAGCTCCGGTTCCCAACGATCAAGGCGAGTTACATGATCCCCCATGTTGTGCAAAAAAGC
GGTTAGCTCCTTCGGTCCTCCGATCGTTGTCAGAAGTAAGTTGGCCGCAGTGTTATCACTCATGGTT
ATGGCAGCACTGCATAATTCTCTTACTGTCATGCCATCCGTAAGATGCTTTTCTGTGACTGGTGAGT
ACTCAACCAAGTCATTCTGAGAATAGTGTATGCGGCGACCGAGTTGCTCTTGCCCGGCGTCAATAC
GGGATAATACCGCGCCACATAGCAGAACTTTAAAAGTGCTCATCATTGGAAAACGTTCTTCGGGG
CGAAAACTCTCAAGGATCTTACCGCTGTTGAGATCCAGTTCGATGTAACCCACTCGTGCACCCAA
CTGATCTTCAGCATCTTTTACTTTCACCAGCGTTTCTGGGTGAGCAAAAACAGGAAGGCAAAATG

CCGCAAAAAAGGGAATAAGGGCGACACGGAAATGTTGAATACTCATACTCTTCCTTTTTCAATATT
ATTGAAGCATTTATCAGGGTTATTGTCTCATGAGCGGATACATATTTGAATGTATTTAGAAAAATAA
ACAAATAGGGGTTCCGCGCACATTTCCCCGAAAAGTGCCACCTGACGCGCCCTGTAGCGGCGCAT
TAAGCGCGGCGGGTGTGGTGGTTACGCGCAGCGTGACCGCTACACTTGCCAGCGCCCTAGCGCCC
GCTCCTTTCGCTTTCTTCCCTTCCTTTCTCGCCACGTTCGCCGGCTTTCCCCGTCAAGCTCTAAATCG
GGGGCTCCCTTTAGGGTTCCGATTTAGTGCTTTACGGCACCTCGACCCCAAAAAACTTGATTAGGG
TGATGGTTCACGTAGTGGGCCATCGCCCTGATAGACGGTTTTTCGCCCTTTGACGTTGGAGTCCAC
GTTCTTTAATAGTGGACTCTTGTTCCAAACTGGAACAACACTCAACCCTATCTCGGTCTATTCTTTT
GATTTATAAGGGATTTTGCCGATTTCGGCCTATTGGTTAAAAAATGAGCTGATTTAACAAAAATTTA
ACGCGAATTTTAACAAAATATTAACGCTTACAATTTCCATTCGCCATTCAGGCTGCGCAACTGTTG
GGAAGGGCGATCGGTGCGGGCCTCTTCGCTATTACGCCAGCTGGCGAAAGGGGGATGTGCTGCA
AGGCGATTAAGTTGGGTAACGCCAGGGTTTTCCCAGTCACGACGTTGTAAAACGACGGCCAGTGC
CAAGCTCCCGCGGGGATCGACTCATAAAATAGTAACCTTCTAATGCGTATCTATTGACTACCAACC
ATTAGTGTGGTTGCAGAAGGCGGAATTTTCCCTTCTTCGAATTTAGCTTGCTTTTTCATTTTTTATTTT
CCATTTTTCAGTTTTTGTTTGTGTCGAATTTAGCCAGTTGCTTCTCCAAGATGAAAAAAACCCCTGC
GCAGTTTCTGTGCTGCAAGATCCTAATCGACTTTTCCACCCCCCACAAAAGTAAATGTTCTTTTGTT
ACATTCGCGTGGGTAGCTAGCTCCCCGAATCTTCAAAGGACTTAGGGACTGCACTACATCAGAGT
GTGTTCACCTGGTTTGCTGCCTGGTTTGAAAGAAAAGAGCAGGGAACTCGCGGGTTCCCGGCGAA
TAATCATGCGATAGTCCTTTGGCCTTCCAAGTCGCATGTAGAGTAGACAACAGACAGGGAGGGCA
GGAAGGATCTTTCACTGAGATCCTGTATCTTGTTGGGTAAGTCGGATGAAAGGGGAATCGTATGAG
ATTGGAGAGGATGCGGAAGAGGTAACGCCTTTTGTTAACTTGTTTAATTATTATGGGGCAGGCGAG
AGGGGGAGGAATGTATGTGTGTGAGGCGGGCGAGACGGAGCCATCCAGGCCAGGTAGAAATAGA
GAAAGCCGAATGTTAGACAATATGGCAGCGTAGTAGAGTAGGTAGGTAGGCAAGTACTGCTAGCA
AAGAGGAGAAGGGTAAGCTCACTCTTCGCATTCCACACCGTTAGTGTGTCAGTTTGAACAAAAAA
ACAATCATCATACCAATTGATGGACTGTGGACTGGCTTTTGGAACGGCTTTTCGGACTGCGATTATT
CGTGAGGAATCAAGGTAGGAATTTGGTCATATTTACGGACAACAGTGGGTGATTCCCATATCGAGT
AGGAAAACGAGATCATGGTATCCTCAGATATGTTGCGGAAATCTGTTCACCGCAAAGTTCAGGGT
GCTCTGGTGGGTTTCGGTTGGTCTTTGCTTTGCTTCTCCCTTGTCTTGCATGTTAATAATAGCCTAGC
CTGTGAGCCGAAACTTAGGGTAGGCTTAGTGTTGGAACGTACATATCTATCACGTTGACTTGGTTTA
ACCAGGCGACCTGGTAGCCAGCCATACCCACACACGTTTTTTGTATCTTCAGTATAGTTGTGAAAA
GTGTAGCGGAAATTTGTGGTCCGAGCAACAGCGTCTTTTTCTAGTAGTGCGGTCGGTTACTTGGTT
GACATTGGTATTTGGACTTTGTTGCTACACCATTCACTACTTGAAGTCGAGTGTGAAGGGTATGATT
TCTAGTGGTGAACACCTTTAGTTACGTAATGTTTTCATTGCTGTTTTACTTGAGATTTCGATTGAGAA
AAAGGTATTTAATAGCTCGAATCAATGTGAGAACAGAGAGAAGATGTTCTTCCCTAACTCGAAAG
GTATATGAGGCTTGTGTTTCTTAGGAGAATTATTATTCTTTTGTTATGTTGCGCTTGTAGTTGGAAAA
GGTGAAGAGACAAAAGCTGGAATTGTGAGCGGATAACAAGCTCAACACTTGAAATTTAGGAAAG
AGCAGAATTTGGCAAAAAAAATAAAAAAAAAAATAAACACACATACTCATCGAG

Sequence S11. Sequence of the 12866 PB plasmid pkLAC2-B3. The coding Sequence of *hasA3* gene is highlighted in green and the coding Sequence of *hasB* gene is highlighted in yellow.

AAGCTTAAAAATGCCAGTACAGTTGACTACAGCCTTGAGGGTTGTCGGAACATCATTGTTCGCTTT
GGCTGTTTTAGGTGGTATCTTGGCTGCTTATGTCACTGGTTATCAATTCATTCATACTGAAAAGCATT

ACTTAAGTTTCGGATTGTATGGAGCTATCTTAGGTTTGCACTTGTTGATACAAAGTTTGTTCGCTTTC
TTGGAGCATAGAAGAATGCGTCGTGCTGGTCAGGCTTTGAAATTGCCTAGTCCTCGTAGAGGTAGT
GTTGCTTTGTGTATTGCAGCTTATCAGGAGGACCCTGATTACTTGAGAAAATGTTTAAGATCTGCTC
AGAGAATTTCATTCCCTGATTTGAAGGTTGTTATGGTAGTGGATGGAAATCGTCAAGAAGACGCAT
ACATGTTAGATATCTTTCATGAAGTATTGGGTGGTACTGAGCAGGCAGGTTTCTTCGTTTGGAGGTC
AAACTTCCACGAAGCTGGTGAAGGAGAGACCGAAGCCTCATTGCAAGAAGGTATGGATAGAGTC
AGGGACGTGGTCAGAGCATCTACCTTCAGTTGCATTATGCAAAAGTGGGGTGGTAAGAGGGAGGT
GATGTACACTGCATTCAAGGCTTTAGGTGATTCAGTGGACTATATCCAAGTGTGTGACTCAGATAC
AGTTTTAGATCCAGCCTGCACTATCGAGATGTTGAGAGTTTTGGAAGAAGATCCACAGGTCGGAG
GTGTGGGTGGAGATGTGCAGATATTGAACAAATACGATAGTTGGATCTCTTTCTTGTCTAGTGTAAG
ATACTGGATGGCTTTCAACGTCGAAAGAGCTTGTCAATCTTACTTTGGTTGCGTTCAATGTATTTCT
GGTCCTTTGGGAATGTACAGAAATTCTTTGTTGCAACAGTTCTTGGAGGACTGGTACCATCAGAAA
TTCTTAGGATCTAAGTGTAGTTTTGGAGATGATAGACATTTGACTAATAGAGTGTTGAGTTTAGGTT
ACAGAACCAAGTACACCGCTAGATCAAAGTGCTTGACTGAAACTCCAACCAAATACTTGAGATG
GTTGAATCAACAAACAAGGTGGTCAAAGAGTTACTTCAGAGAATGGTTGTATAACTCTTTATGGTT
TCACAAGCATCACTTATGGATGACTTATGAATCTGTTGTCACCGGTTTCTTTCCATTCTTCTTAATCG
CTACAGTGATTCAGTTGTTTTACAGAGGTAGAATTTGGAATATTTTATTGTTCTTGTTGACTGTCCAA
TTAGTAGGTATCATCAAAGCTACTTACGCTTGTTTCTTACGTGGTAACGCCGAAATGATCTTCATGT
CATTGTATAGTTTGTTATACATGTCATCATTGTTGCCAGCCAAGATTTTCGCTATCGCCACCATCAAT
AAGTCAGGTTGGGGTACTAGTGGAAGGAAAACAATTGTTGTCAACTTCATTGGATTGATACCAGT
GAGTATTTGGGTAGCAGTATTGTTAGGTGGTTTGGCCTATACTGCTTATTGTCAAGACTTGTTCTCAG
AAACTGAATTAGCATTCTTGGTTTCTGGTGCAATCTTATACGGTTGCTATTGGGTTGCTTTGTTAATG
TTATACTTGGCAATCATTGCTAGAAGATGCGGAAAGAAACCAGAACAGTACTCTTTAGCATTTGCC
GAAGTTTAAAGGCCTTGAATCGAGAATTTATACTTAGATAAGTATGTACTTACAGGTATATTTCTATG
AGATACTGATGTATACATGCATGATAATATTTAAACGGTTATTAGTGCCGATTGTCTTGTGCGATAAT
GACGTTCCTATCAAAGCAATACACTTACCACCTATTACATGGGCCAAGAAAATATTTTCGAACTTG
TTTAGAATATTAGCACAGAGTATATGATGTTATCCGTTAGATTATGCATGATTCATTCCTACAACTTT
TTCGTAGCATAAGGATTAATTACTTGGATGCCAATAAAAAAAAAAAAACATCGAGAAAATTTCAGC
ATGCTCAGAAACAATTGCAGTGTATCAAAGTAAAAAAAAGATTTTCACTACATGTTCCTTTTGAAG
AAAGAAAATCATGGAACATTAGATTTACAAAAATTTAACCACCGCTGATTAACGATTAGACCGTTA
AGCGCACAACAGGTTATTAGTACAGAGAAAGCATTCTGTGGTGTTGCCCCGGACTTTCTTTTGCGA
CATAGGTAAATCGAATACCATCATACTATCTTTTCCAATGACTCCCTAAAGAAAGACTCTTCTTCGA
TGTTGTATACGTTGGAGCATAGGGCAAGAATTGTGGCTTGAGATCATCCTTTTGTTGTTTCCGGGTG
TACAATATGGACTTCCTCTTTTCTGGCAACCAAACCCATACATCGGGATTCCTATAATACCTTCGTT
GGTCTCCCTAACATGTAGGTGGCGGAGGGGAGATATACAATAGAACAGATACCAGACAAGACATA
ATGGGCTAAACAAGACTACACCAATTACACTGCCTCATTGATGGTGGTACATAACGAACTAATACT
GTAGCCCTAGACTTGATAGCCATCATCATATCGAAGTTTCACTACCCTTTTTCCATTTGCCATCTATT
GAAGTAATAATAGGCGCATGCAACTTCTTTTCTTTTTTTTTCTTTTCTCTCTCCCCCGTTGTTGTCTCA
CCATATCCGCAATGACAAAAAAAATGATGGAAGACACTAAAGGAAAAAATTAACGACAAAGACA
GCACCAACAGATGTCGTTGTTCCAGAGCTGATGAGGGGTATCTCGAAGCACACGAAACTTTTTCC
TTCCTTCATTCACGCACACTACTCTCTAATGAGCAACGGTATACGGCCTTCCTTCCAGTTACTTGAA
TTTGAAATAAAAAAAAGTTTGCTGTCTTGCTATCAAGTATAAATAGACCTGCAATTATTAATCTTTT
GTTTCCTCGTCATTGTTCTCGTTCCCTTTCTTCCTTGTTTCTTTTTCTGCACAATATTTCAAGCTATAC
CAAGCATACAATCAAGCAATTCCAGATCTGCCACCATGCCTCAATCCTGGGAAGAACTGGCCGCT

GATAAGCGCGCCCGCCTCGCAAAAACCATCCCTGATGAATGGAAAGTCCAGACGCTGCCTGCGG
AAGACAGCGTTATTGATTTCCCAAAGAAATCGGGCATCCTTTCAGAGGCCGAACTGAAGATCACA
GAGGCTTCCGCTGCGGATCTTGTGTCCAAGCTGGCGGCCGGAGAGTTGACCTCGGTGGAAGTTAC
GCTAGCATTCTGTAAACGGGCAGCAATCGCCCAGCAGTTAACAAACTGCGCCCACGAGTTCTTCC
CTGACGCCGCTCTCGCGCAGGCAAGGGAACTCGATGAATACTACGCAAAGCACAAGAGACCCGT
TGGTCCACTTCATGGCCTCCCCATCTCTCTCAAAGACCAGCTTCGAGTCAAGGGCTACGAAACAT
CAATGGGCTACATCTCATGGCTAAACAAGTACGACGAAGGGGACTCGGTTCTGACAACCATGCTC
CGCAAAGCCGGTGCCGTCTTCTACGTCAAGACCTCTGTCCCGCAGACCCTGATGGTCTGCGAGAC
AGTCAACAACATCATCGGGCGCACCGTCAACCCACGCAACAAGAACTGGTCGTGCGGCGGCAGT
TCTGGTGGTGAGGGTGCGATCGTTGGGATTCGTGGTGGCGTCATCGGTGTAGGAACGGACATCGG
TGGCTCGATTCGAGTGCCGGCCGCGTTCAACTTCCTGTACGGTCTAAGGCCGAGTCATGGGCGGCT
GCCGTATGCAAAGATGGCGAACAGCATGGAGGGTCAGGAGACGGTGCACAGCGTTGTCGGGCCG
ATTACGCACTCTGTTGAGGACCTCCGCCTCTTCACCAAATCCGTCCTCGGTCAGGAGCCTTGGAAA
TACGACTCCAAGGTCATCCCCATGCCCTGGCGCCAGTCCGAGTCGGACATTATTGCCTCCAAGATC
AAGAACGGCGGGCTCAATATCGGCTACTACAACTTCGACGGCAATGTCCTTCCACACCCTCCTATC
CTGCGCGGCGTGGAAACTACCGTCGCCGCACTCGCCAAAGCCGGTCACACCGTGACCCCGTGGA
CGCCATACAAGCACGATTTCGGCCACGATCTCATCTCCCATATCTACGCGGCTGACGGCAGCGCCG
ACGTAATGCGCGACATCAGTGCATCCGGCGAGCCGGCGATTCCAAATATCAAAGACCTACTGAAC
CCGAACATCAAAGCTGTTAACATGAACGAGCTCTGGGACACGCATCTCCAGAAGTGGAATTACCA
GATGGAGTACCTTGAGAAATGGCGGGAGGCTGAAGAAAAGGCCGGGAAGGAACTGGACGCCAT
CATCGCGCCGATTACGCCTACCGCTGCGGTACGGCATGACCAGTTCCGGTACTATGGGTATGCCTC
TGTGATCAACCTGCTGGATTTCACGAGCGTGGTTGTTCCGGTTACCTTTGCGGATAAGAACATCGA
TAAGAAGAATGAGAGTTTCAAGGCGGTTAGTGAGCTTGATGCCCTCGTGCAGGAAGAGTATGATC
CGGAGGCGTATCATGGGGCACCGGTTGCAGTGCAGGTTATCGGACGGAGACTCAGTGAAGAGAG
GACGTTGGCGATTGCAGAGGAAGTGGGGAAGTTGCTGGGAAATGTGGTGACTCCATAGCCCGGG
GGGGGCTCGATCCCCTCGCGAGTTGGTTCAGCTGCTGCCTGAGGGTACCGGCCGCAAATTAAAGC
CTTCGAGCGTCCCAAAACCTTCTCAAGCAAGGTTTTCAGTATAATGTTACATGCGTACACGCGTCT
GTACAGAAAAAAAAGAAAAATTTGAAATATAAATAACGTTCTTAATACTAACATAACTATAAAAA
AATAAATAGGGACCTAGACTTCAGGTTGTCTAACTCCTTCCTTTTCGGTTAGAGCGGATGTGGGGG
GAGGGCGTGAATGTAAGCGTGACATAACTAATTACATGACTCGAGGTCGACTTACACACGTTGCTT
CTTGTGTGGCAAGTCCTGTAAACCGAACTTAGGGATATCAGCAGTTGGAGTGAATGGTATTCTTTT
TGAAGCTACCTTCTTTCCGATGGTTTCCACCTGAAATCCAATGTTTTGCAATTCTCCATGCAATTCA
TCTAAAACACGTCTACCATCGAATATGAAAGCAGGCTTTAACATCATCCTATGGATTCTATTGAAAT
CTAATTCCTTGAACATATCCCATTCAGTACAAATGACCATAGCGTGTGCATTCTCACAGGCTTCGTA
CAAATCTGTACTTATGTGGACCAATTGAGAAACCCTGTCGTCAGCTGCAACACCAGGTTGACTCA
AGTCAGTGATGATCTGCTCACGTGGGACCTTTGGATCGTAGATATGTAACTTAGCACCTTCATCCAT
CAAATACTTAGAGATATAGATTGAACTACTCTCTCTAGTATCACCTGTATCCTTCTTGAAAGCGAAA
CCTAACAATGCGATTTTCTTATCGGTCACGGTGTTAAACAAACAATCGATTATCCTAGTTGTAAAAC
GTCTCCTTTGATAATCATTCATATCAATCACTTGTTGCCAGTACTTGGCCACTTCGTGCAAGTTTAAC
ACCTCACACAAGTAAACCAAGTTCAAAACGTCCTTCTGAAAACATGAACCTCCAAATCCCACTG
AAGCCTTCAAAAACTTGTTACCAATTCTTTGATCCATACCAATAGCTCTGGCAACCTCTTCAACGT
CAGCTCCTGTGGCTTCACATAAGGCACTAATTGAGTTGATTGAAGAAATTCTTTGAGCTAAGAATG
CGTTGGCTGCTAACTTACTCAACTCAGAAGACCAGGTGTTTGTGGTTATGATTTTCTCAGATGGTAC
CCAGTGTTCGTATACGTCACACAAAGCTCTAACAGCTTTCTGACCTTCAGGGGTTTCGTCACCACC

TATCAAAACTCTATCAGGGTTCTTCAAATCCTTAATGGCTGTACCCTCTGCCAAAAACTCTGGGTTA
CTCAATACCTGCAAGTTCAAATCTGGTTTAGTATTTGCATCGAAGATACGTCTTATTGATTCAGCAG
CTCTAACTGGCACAGTAGATTTCTCTGTAACAATCTTGTATCCGTTACTATTCTGTACTATTCTTCTA
GCGCAAGCCTCAATGTATTTCAAGTCGGCTGCCCTTCCCTTACCCATACCGTAAGTTTTTGTTGGAG
TGTTGACTGAGATGAACACCAAATCAGCTTCTTGAATTGCACCATCAATGTCAGTTGAGTAGAAC
AAATTCTTTCCCCTGCATGACTCTACGACTTCCTTCAAACCTGGTTCGTAGATAGGCAAAGTGTCA
CTATTCCAAGCATTGATCCTGGCTTGGTTCACATCCACAACAGTGACCTTAATGTCAGGGCACATC
TGTGCAATGACAGAACAGGTTGGACCACCGACGTAACCGGCACCAATACAACAAATCTTCTTGAT
CTGAAACATTTTTGGATCCACTAGTTCTAGATCCGTCGAAACTAAGTTCTTGGTGTTTTAAAACTAA
AAAAAAGACTAACTATAAAAGTAGAATTTAAGAAGTTTAAGAAATAGATTTACAGAATTACAATC
AATACCTACCGTCTTTATATACTTATTAGTCAAGTAGGGGAATAATTTCAGGGAACTGGTTTAAACC
TTTTTTTTCAGCTTTTTCCAAATCAGAGAGAGCAGAAGGTAATAGAAGGTGTAAGAAAATGAGAT
AGATACATGCGTGGGTCAATTGCCTTGTGTCATCATTTACTCCAGGCAGGTTGCATCACTCCATTGA
GGTTGTGCCCGTTTTTTGCCTGTTTGTGCCCTGTTCTCTGTAGTTGCGCTAAGAGAATGGACCTATG
AACTGATGGTTGGTGAAGAAAACAATATTTTGGTGCTGGGATTCTTTTTTTTTCTGGATGCCAGCTT
AAAAAGCGGGCTCCATTATATTTAGTGGATGCCAGGAATAAACCTGTTCACCCAAGCACCATCAG
TGTTATATATTCTGTGTAACCCGCCCCCTATTTTGGCATGTACGGGTTACAGCAGAATTAAAAGGCT
AATTTTTTGACTAAATAAAGTTAGGAAAATCACTACTATTAATTATTTACGTATTCTTTGAAATGGCA
GTATTGATAATGATAAACTCGAGCCTGAGGCTGGACGACCTCGCGGAGTTCTACCGGCAGTGCAA
ATCCGTCGGCATCCAGGAAACCAGCAGCGGCTATCCGCGCATCCATGCCCCCGAACTGCAGGAGT
GGGGAGGCACGATGGCCGCTTTGGTCGATCTAGATTACGTGGAAGAAAGGTAGTAAAAGTAGTAG
TATAAGTAGTAAAAAGAGGTAAAAAGAGAAAACCGGCTACATACTAGAGAAGCACGTACACAAA
AACTCATAGGCACTTCATCATACGACAGTTTCTTGATGCATTATAATAGTGTATTAGATATTTTCAGA
AATATGCATAGAACCTCCTCTTGCCTTTACTTTTTATACATAGAACATTGGCAGATTTACTTACACTA
CTTTGTTTCTACGCCATTTCTTTTGTTTTCAACACTTAGACAAGTTGTTGAGAACCGGACTACTAAA
AAGCAATGTTCCCACTGAAAATCATGTACCTGCAGCATAATAACCCCCTAATTCTGCATCGATCCA
GTATGTTTTTTTTTCTCTACTCATTTTTACCTGAAGATAGAGCTTCTAAAACAAAAAAAATCAGTGA
TTACATGCATATTGTGTGTTCTAGTAACCAAAGGAAAGGAACAGATAGATAAAATTCCGAGACTGT
CAAATTAGGTTTTTTTCTTTTTTTTTGGCGGGAGTCAGTGGGCCGAAATATGTTCTTGGCCTAGAAC
TTAATCTGGTTTGATCATGCCAATACTTGCCTGAGTGCCCGACTTTTTGCCCACCCTCTTGCCTTCTG
TCATCCTTCAAAACCCACCTGTTTTCCAGCCGTATCTTCGCTCGCATCTACACATACTGTGCCATAT
CTTGTGTGTAGCCGGACGTGACTATGACCAAAAACAAACAAGGAGAACTGTTCGCCGATTTGTAA
CACTCCTGCATCCATCCAAGTGGGTATGCGCTATGCAATGTTAAGCTAGGTCAGGTCAGACCAGGT
CCAAGGACAGCAACTTGACTGTATGCAACCTTTACCATCTTTGCACAGAACATACTTGTAGCTAGC
TAGTTACACTTATGGACCGAAAAGGCACCCCACCATGTCTGTCCGGCTTTAGAGTACGGCCGCAG
ACCGCTGATTTGCCTTGCCAAGCAGTAGTCACAATGCATCGCATGAGCACACGGGCACGGGCACG
GGCACAGGAACCATTGGCAAAAATACCAGATACACTATACCGACGTATATCAAGCCCAAGTTTAA
AATTCCTAAATTTCCGCGGCTACTTTTCAATTCCCTATAGTGAGTCGTATTAAATTCGTAATCATGTC
ATAGCTGTTTCCTGTGTGAAATTGTTATCCGCTCACAATTCCACACAACATACGAGCCGGAAGCAT
AAAGTGTAAAGCCTGGGGTGCCTAATGAGTGAGCTAACTCACATTAATTGCGTTGCGCTCACTGCC
CGCTTTCCAGTCGGGAAACCTGTCGTGCCAGCTGCATTAATGAATCGGCCAACGCGCGGGGAGAG
GCGGTTTGCGTATTGGGCGCTCTTCCGCTTCCTCGCTCACTGACTCGCTGCGCTCGGTCGTTCGGCT
GCGGCGAGCGGTATCAGCTCACTCAAAGGCGGTAATACGGTTATCCACAGAATCAGGGGATAACG
CAGGAAAGAACATGTGAGCAAAAGGCCAGCAAAAGGCCAGGAACCGTAAAAAGGCCGCGTTGC

TGGCGTTTTTCCATAGGCTCCGCCCCCCTGACGAGCATCACAAAAATCGACGCTCAAGTCAGAGG
TGGCGAAACCCGACAGGACTATAAAGATACCAGGCGTTTCCCCCTGGAAGCTCCCTCGTGCGCTC
TCCTGTTCCGACCCTGCCGCTTACCGGATACCTGTCCGCCTTTCTCCCTTCGGGAAGCGTGGCGCTT
TCTCATAGCTCACGCTGTAGGTATCTCAGTTCGGTGTAGGTCGTTCGCTCCAAGCTGGGCTGTGTGC
ACGAACCCCCCGTTCAGCCCGACCGCTGCGCCTTATCCGGTAACTATCGTCTTGAGTCCAACCCGG
TAAGACACGACTTATCGCCACTGGCAGCAGCCACTGGTAACAGGATTAGCAGAGCGAGGTATGTA
GGCGGTGCTACAGAGTTCTTGAAGTGGTGGCCTAACTACGGCTACACTAGAAGAACAGTATTTGG
TATCTGCGCTCTGCTGAAGCCAGTTACCTTCGGAAAAAGAGTTGGTAGCTCTTGATCCGGCAAAC
AAACCACCGCTGGTAGCGGTGGTTTTTTTGTTTGCAAGCAGCAGATTACGCGCAGAAAAAAAGGA
TCTCAAGAAGATCCTTTGATCTTTTCTACGGGGTCTGACGCTCAGTGGAACGAAAACTCACGTTAA
GGGATTTTGGTCATGAGATTATCAAAAAGGATCTTCACCTAGATCCTTTTAAATTAAAAATGAAGTT
TTAAATCAATCTAAAGTATATATGAGTAAACTTGGTCTGACAGTTACCAATGCTTAATCAGTGAGGC
ACCTATCTCAGCGATCTGTCTATTTCGTTCATCCATAGTTGCCTGACTCCCCGTCGTGTAGATAACTA
CGATACGGGAGGGCTTACCATCTGGCCCCAGTGCTGCAATGATACCGCGAGACCCACGCTCACCG
GCTCCAGATTTATCAGCAATAAACCAGCCAGCCGGAAGGGCCGAGCGCAGAAGTGGTCCTGCAA
CTTTATCCGCCTCCATCCAGTCTATTAATTGTTGCCGGGAAGCTAGAGTAAGTAGTTCGCCAGTTAA
TAGTTTGCGCAACGTTGTTGCCATTGCTACAGGCATCGTGGTGTCACGCTCGTCGTTTGGTATGGCT
TCATTCAGCTCCGGTTCCCAACGATCAAGGCGAGTTACATGATCCCCCATGTTGTGCAAAAAAGC
GGTTAGCTCCTTCGGTCCTCCGATCGTTGTCAGAAGTAAGTTGGCCGCAGTGTTATCACTCATGGTT
ATGGCAGCACTGCATAATTCTCTTACTGTCATGCCATCCGTAAGATGCTTTTCTGTGACTGGTGAGT
ACTCAACCAAGTCATTCTGAGAATAGTGTATGCGGCGACCGAGTTGCTCTTGCCCGGCGTCAATAC
GGGATAATACCGCGCCACATAGCAGAACTTTAAAAGTGCTCATCATTGGAAAACGTTCTTCGGGG
CGAAAACTCTCAAGGATCTTACCGCTGTTGAGATCCAGTTCGATGTAACCCACTCGTGCACCCAA
CTGATCTTCAGCATCTTTTACTTTCACCAGCGTTTCTGGGTGAGCAAAAACAGGAAGGCAAAATG
CCGCAAAAAAGGGAATAAGGGCGACACGGAAATGTTGAATACTCATACTCTTCCTTTTTCAATATT
ATTGAAGCATTTATCAGGGTTATTGTCTCATGAGCGGATACATATTTGAATGTATTTAGAAAAATAA
ACAAATAGGGGTTCCGCGCACATTTCCCCGAAAAGTGCCACCTGACGCGCCCTGTAGCGGCGCAT
TAAGCGCGGCGGGTGTGGTGGTTACGCGCAGCGTGACCGCTACACTTGCCAGCGCCCTAGCGCCC
GCTCCTTTCGCTTTCTTCCCTTCCTTTCTCGCCACGTTCGCCGGCTTTCCCCGTCAAGCTCTAAATCG
GGGGCTCCCTTTAGGGTTCCGATTTAGTGCTTTACGGCACCTCGACCCCAAAAAACTTGATTAGGG
TGATGGTTCACGTAGTGGGCCATCGCCCTGATAGACGGTTTTTCGCCCTTTGACGTTGGAGTCCAC
GTTCTTTAATAGTGGACTCTTGTTCCAAACTGGAACAACACTCAACCCTATCTCGGTCTATTCTTTT
GATTTATAAGGGATTTTGCCGATTTCGGCCTATTGGTTAAAAAATGAGCTGATTTAACAAAAATTTA
ACGCGAATTTTAACAAAATATTAACGCTTACAATTTCCATTCGCCATTCAGGCTGCGCAACTGTTG
GGAAGGGCGATCGGTGCGGGCCTCTTCGCTATTACGCCAGCTGGCGAAAGGGGGATGTGCTGCA
AGGCGATTAAGTTGGGTAACGCCAGGGTTTTCCCAGTCACGACGTTGTAAAACGACGGCCAGTGC
CAAGCTCCCGCGGGGATCGACTCATAAAATAGTAACCTTCTAATGCGTATCTATTGACTACCAACC
ATTAGTGTGGTTGCAGAAGGCGGAATTTTCCCTTCTTCGAATTTAGCTTGCTTTTTCATTTTTTATTTT
CCATTTTTCAGTTTTTGTTTGTGTCGAATTTAGCCAGTTGCTTCTCCAAGATGAAAAAAACCCCTGC
GCAGTTTCTGTGCTGCAAGATCCTAATCGACTTTTCCACCCCCCACAAAAGTAAATGTTCTTTTGTT
ACATTCGCGTGGGTAGCTAGCTCCCCGAATCTTCAAAGGACTTAGGGACTGCACTACATCAGAGT
GTGTTCACCTGGTTTGCTGCCTGGTTTGAAAGAAAAGAGCAGGGAACTCGCGGGTTCCCGGCGAA
TAATCATGCGATAGTCCTTTGGCCTTCCAAGTCGCATGTAGAGTAGACAACAGACAGGGAGGGCA
GGAAGGATCTTTCACTGAGATCCTGTATCTTGTTGGGTAAGTCGGATGAAAGGGGAATCGTATGAG

ATTGGAGAGGATGCGGAAGAGGTAACGCCTTTTGTTAACTTGTTTAATTATTATGGGGCAGGCGAG
AGGGGGAGGAATGTATGTGTGTGAGGCGGGCGAGACGGAGCCATCCAGGCCAGGTAGAAATAGA
GAAAGCCGAATGTTAGACAATATGGCAGCGTAGTAGAGTAGGTAGGTAGGCAAGTACTGCTAGCA
AAGAGGAGAAGGGTAAGCTCACTCTTCGCATTCCACACCGTTAGTGTGTCAGTTTGAACAAAAAA
ACAATCATCATACCAATTGATGGACTGTGGACTGGCTTTTGGAACGGCTTTTCGGACTGCGATTATT
CGTGAGGAATCAAGGTAGGAATTTGGTCATATTTACGGACAACAGTGGGTGATTCCCATATCGAGT
AGGAAAACGAGATCATGGTATCCTCAGATATGTTGCGGAAATCTGTTCACCGCAAAGTTCAGGGT
GCTCTGGTGGGTTTCGGTTGGTCTTTGCTTTGCTTCTCCCTTGTCTTGCATGTTAATAATAGCCTAGC
CTGTGAGCCGAAACTTAGGGTAGGCTTAGTGTTGGAACGTACATATCTATCACGTTGACTTGGTTTA
ACCAGGCGACCTGGTAGCCAGCCATACCCACACACGTTTTTTGTATCTTCAGTATAGTTGTGAAAA
GTGTAGCGGAAATTTGTGGTCCGAGCAACAGCGTCTTTTTCTAGTAGTGCGGTCGGTTACTTGGTT
GACATTGGTATTTGGACTTTGTTGCTACACCATTCACTACTTGAAGTCGAGTGTGAAGGGTATGATT
TCTAGTGGTGAACACCTTTAGTTACGTAATGTTTTCATTGCTGTTTTACTTGAGATTTCGATTGAGAA
AAAGGTATTTAATAGCTCGAATCAATGTGAGAACAGAGAGAAGATGTTCTTCCCTAACTCGAAAG
GTATATGAGGCTTGTGTTTCTTAGGAGAATTATTATTCTTTTGTTATGTTGCGCTTGTAGTTGGAAAA
GGTGAAGAGACAAAAGCTGGAATTGTGAGCGGATAACAAGCTCAACACTTGAAATTTAGGAAAG
AGCAGAATTTGGCAAAAAAAATAAAAAAAAAATAAACACACATACTCATCGAG

Table S1. Primers used in this study. The restriction enzyme sites are highlighted in blue

Primer Name	Sequence (5′-3′)	Enzyme Site	Fragment size
HASB-GPD-F	AAA*CCTNAGG*GTACCGGCCGCAAATTAAAG*	*Bsu36I*	2454
HASB-GPD-R	AAA*CCTNAGG*CTCGAGTTTATCATTATCAATACTGCC*	*Bsu36I*	
HASA2-F	CCC*AAGCTT*AAAAATGCACTGTGAAAGATTC	*HindIII*	1681
HASA2-R	AAA*AGGCCT*TCAAACATCCAAGACCATAT	*StuI*	
HASA3-F	CCC*AAGCTT*AAAAATGCCAGTACAGTTGAC	*HindIII*	1684
HASA3-R	AAA*AGGCCT*TTAAACTTCGGCAAATGCTAAA	*StuI*	
HASB-F	ATGTTTCAGATCAAGAAGATTTGTTGTATTGG	--	1485
HASB-R	TTACACACGTTGCTTCTTGTGTG	--	
HASA1/P-F	AATTGTGAGCGGATAACAAG	--	1755 for hasA1
HASA1/P-R	TAAGTATAAATTCTCGATT	--	2948 for hasAP
Rt-PCR HASAP F	TCCATTCGAGGTGATTGTCACAG	--	129
Rt-PCR HASAP R	GCAGCACTAGCTTGGAAACC	--	
Rt-PCR HASA1 F	TTTTGCAGCATGGTTGAGAGG	--	135
Rt-PCR HASA1 R	CCTGAAGTACCCCAACCTGA	--	
Rt-PCR HASA2 F	GCAGAAATGGGGTGGTAAGAGA	--	158

Rt-PCR HASA2 R	CACCCACCATTGGATCTTCTTCTAAA	--	
Rt-PCR HASA3 F	GTTTGGAGGTCAAACTTCCACG	--	133
Rt-PCR HASA3 R	TCTTACCACCCCACTTTTGCATA	--	
Rt-PCR HASB F	GATGTGAACCAAGCCAGGATC	--	142
Rt-PCR HASB R	CAGCTTCTTGAATTGCACCATCAAT	--	
Rt-PCR ACTINKL F	TCTCCTTGCCTCACGCTATC	--	124
Rt-PCR ACTINKL R	CGGACGATTTCTCTTTCAGCG	--	

* N means any base

Figure S12. RNA integrity of the strains used in this study analyzed in 1% agarose gel. In wells A to C RNA from strain GG799; In wells D to F RNA from BAP strain; In wells G to I RNA from BA1 strain; In wells J to L RNA from BA2 strain and in wells M to O RNA from BA3 strain.

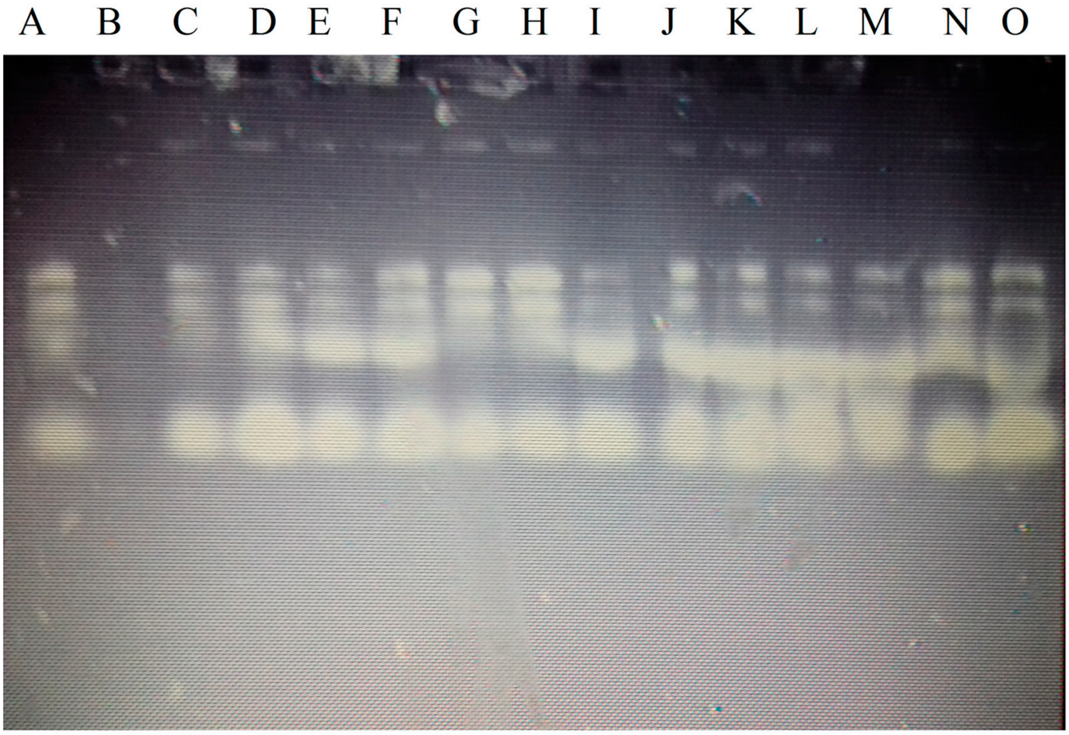

Figure S13. The RNA integrity of the strains used in this study after DNAseI treatment analysed in 1% agarose gel. In wells A to C RNA from strain GG799; In wells D to F RNA from BAP strain; In wells G to I RNA from BA1 strain; In wells J to L RNA from BA2 strain and in wells M to O RNA from BA3 strain.

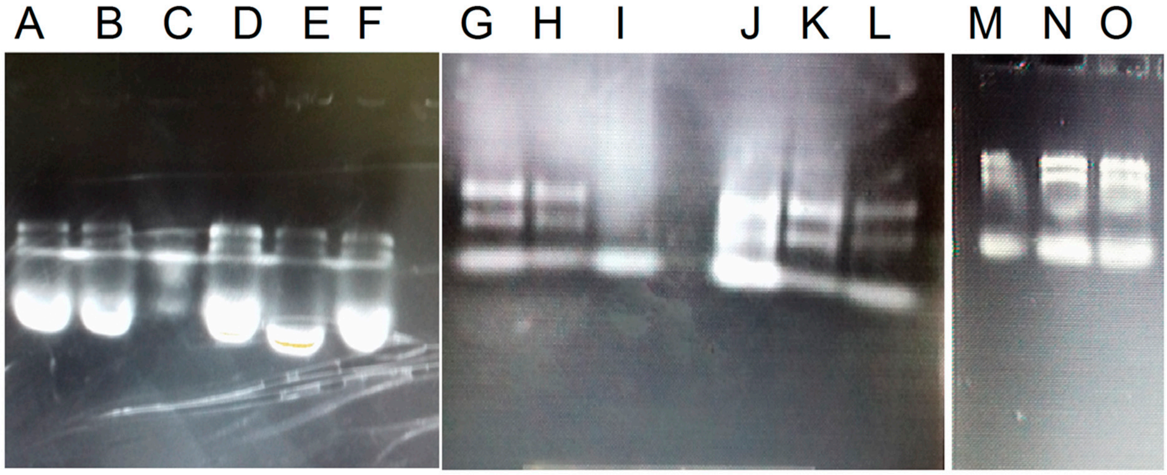

Figure S14. Melt Curve obtained during amplification of hasAP transcipts in RT-qPCR.

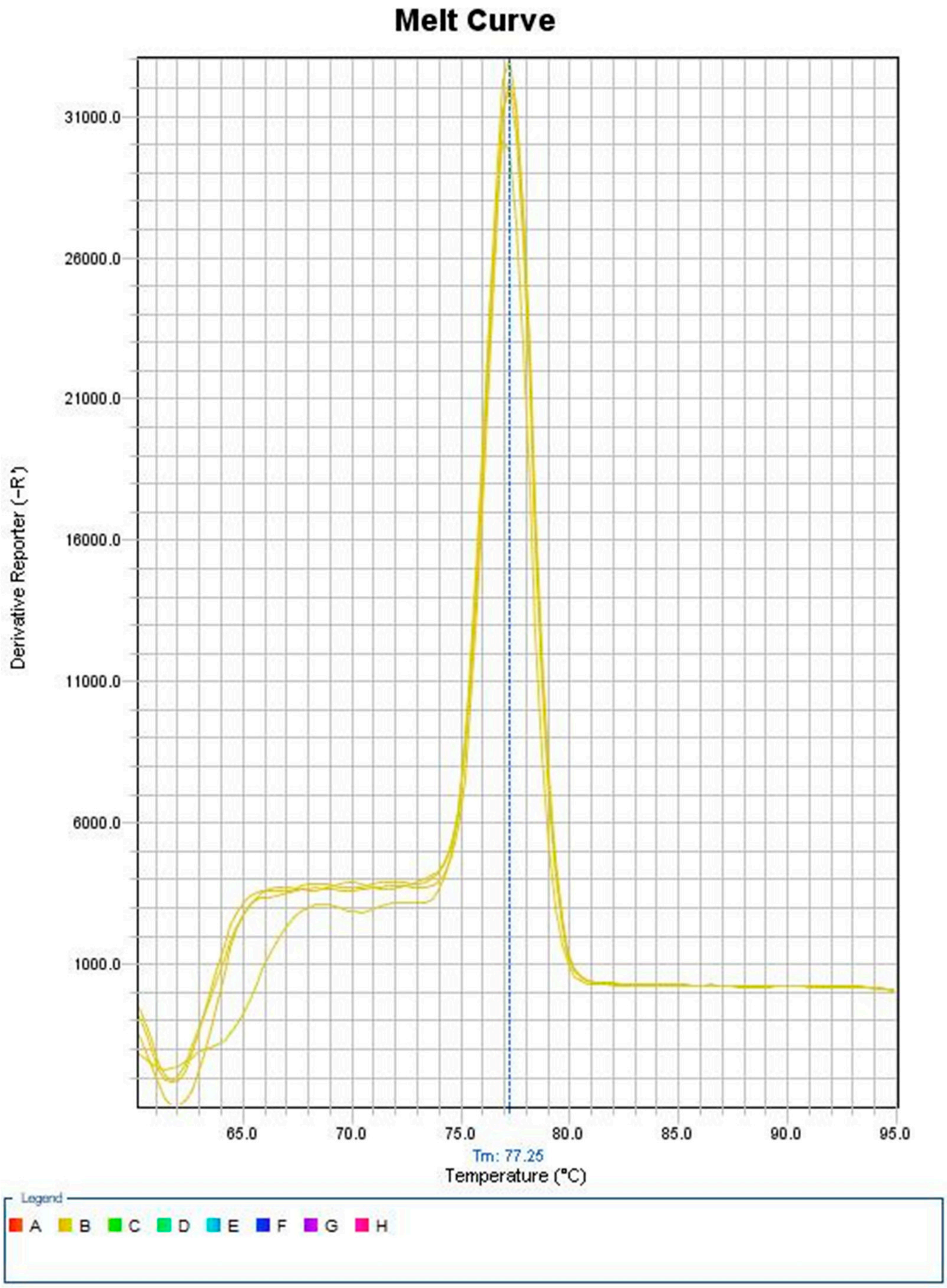
Melt Curve
31000.0
26000.0
21000.0
16000.0
11000.0
6000.0
1000.0
Derivative Reporter (-R')
65.0
70.0
75.0
80.0
85.0
90.0
95.0
Tm: 77.25
Temperature (°C)
Legend
A
B
C
D
E
F
G
H

Figure S15. Melt Curve obtained during amplification of hasA1 transcipts in RT-qPCR.

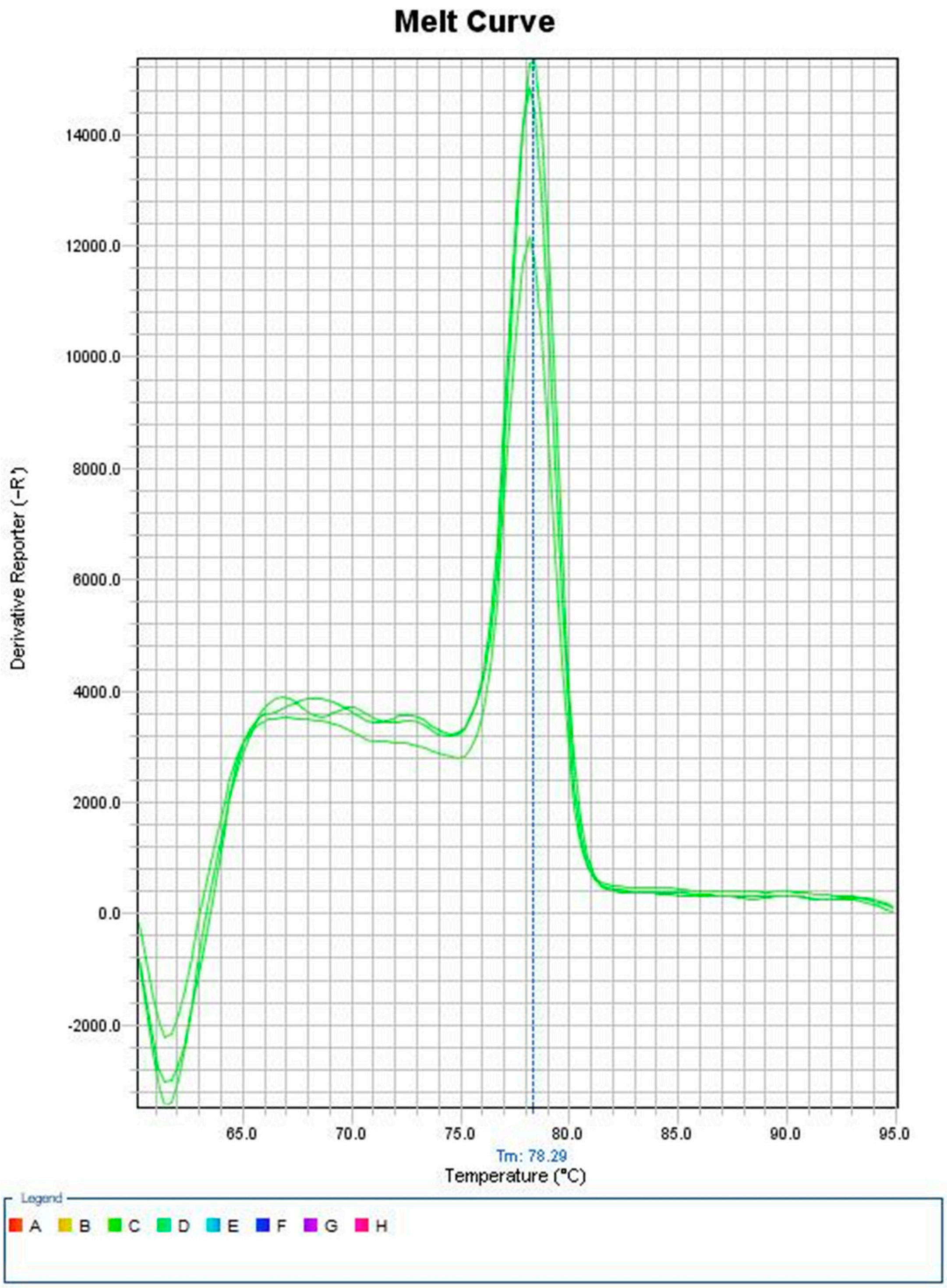

Figure S16. Melt Curve obtained during amplification of hasA3 transcipts in RT-qPCR.

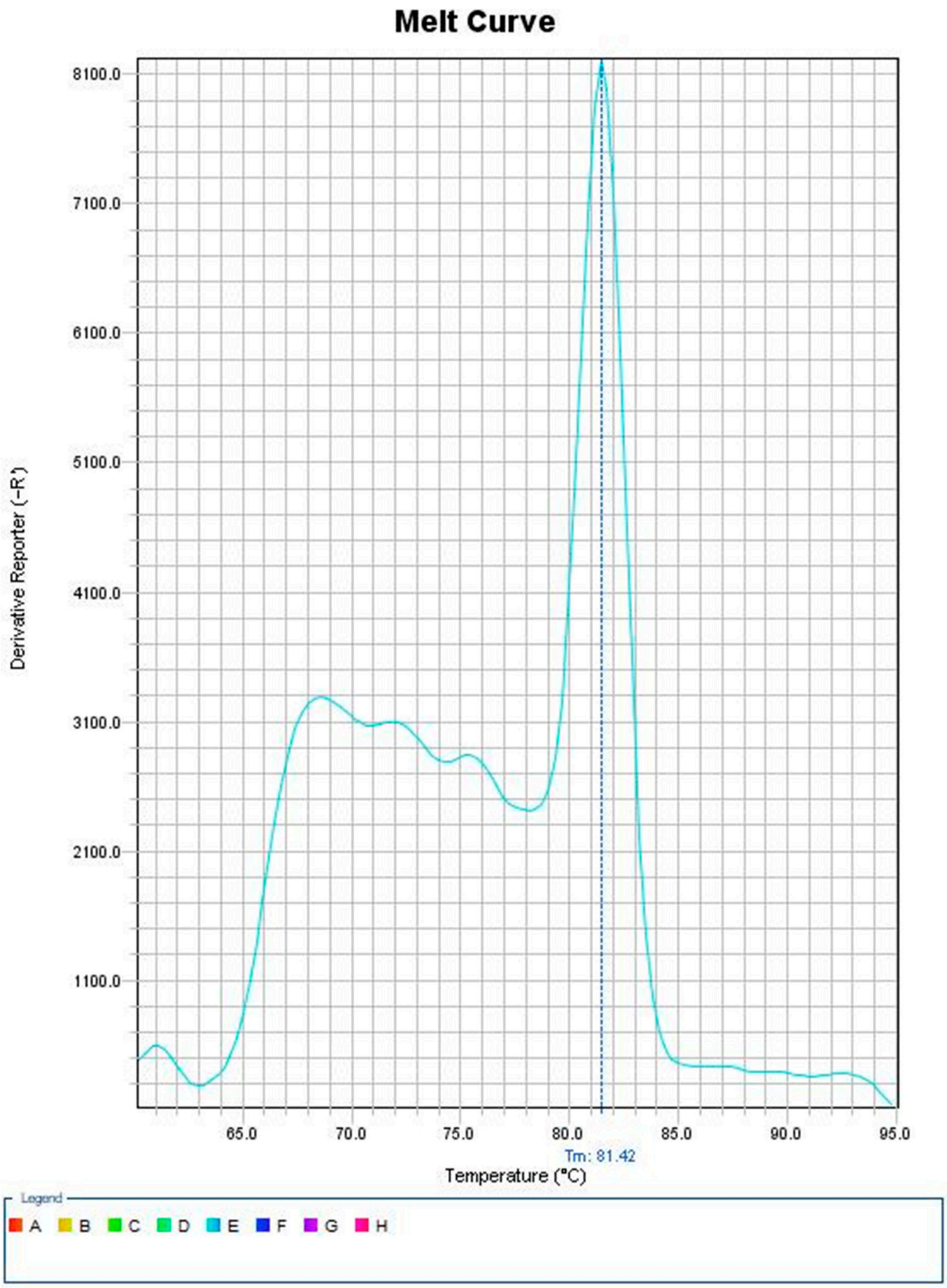

Figure S17. Melt Curve obtained during amplification of hasB transcipts in RT-qPCR.

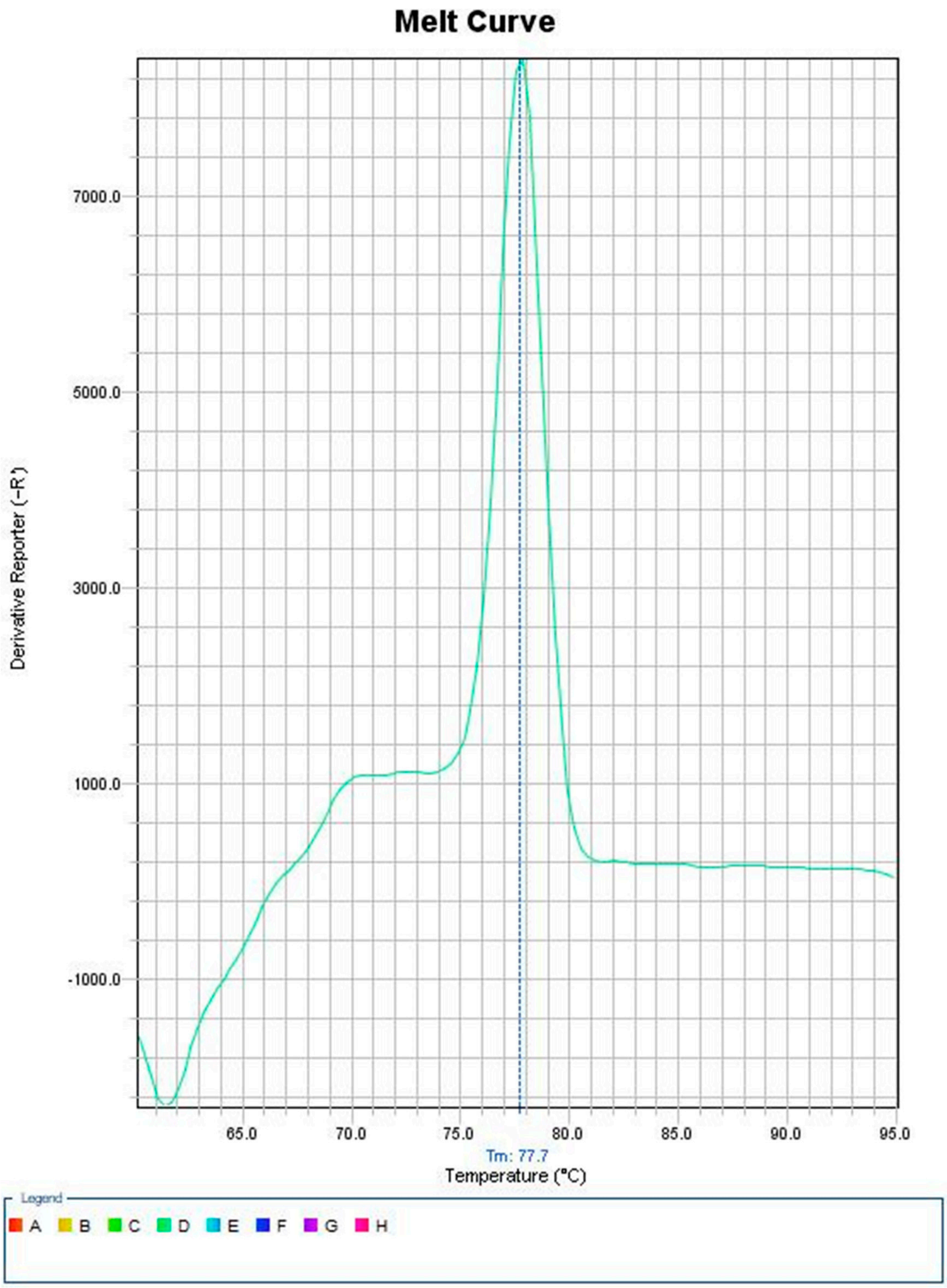

.

Figure S18. Melt Curve obtained during amplification of Actin transcipts in RT-qPCR.

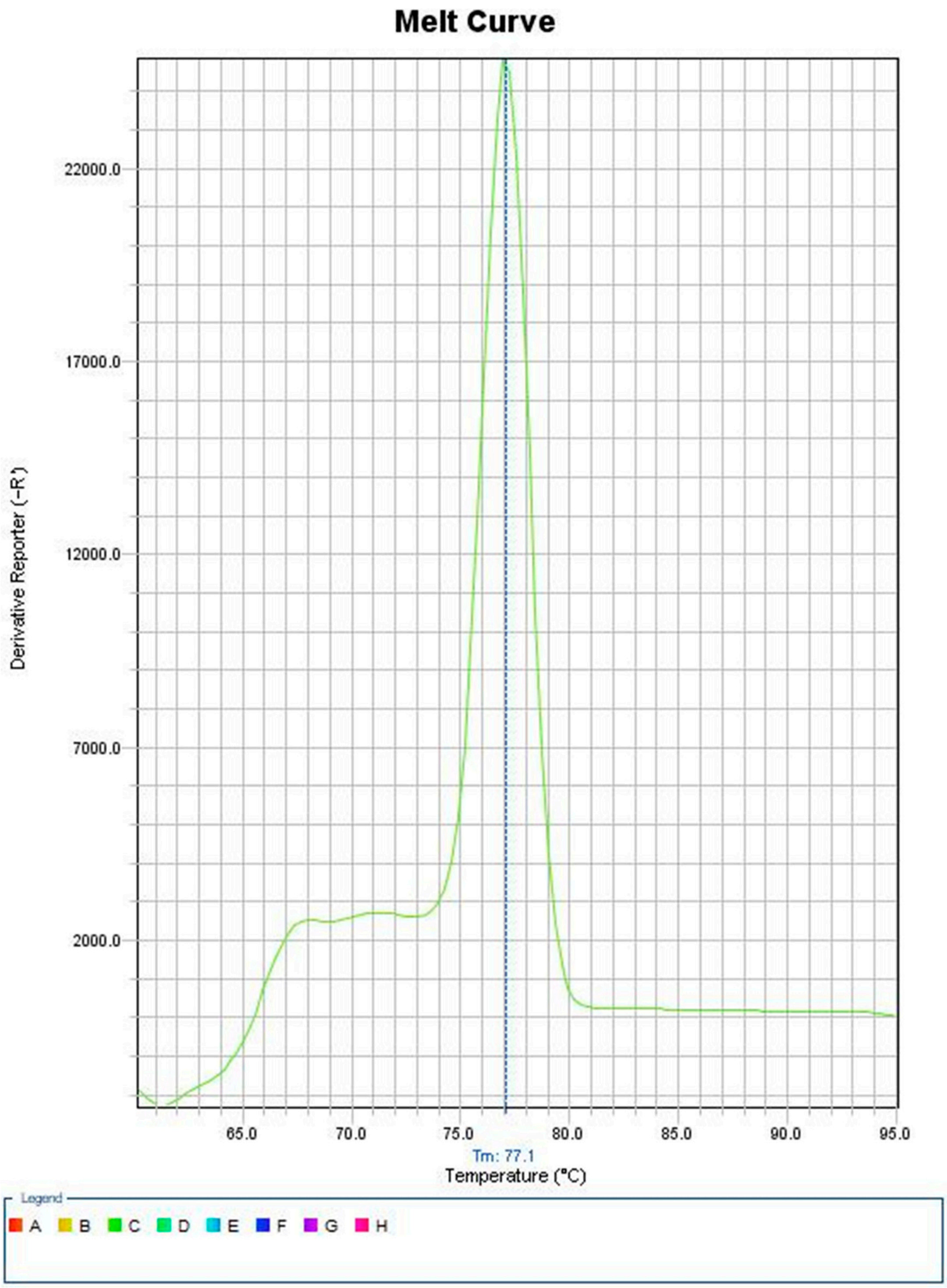

Figure S19. Growth kinetics of the K. lactis wild-type strain and strains constructed in the study. Wild type GG799 (diamond); BA1 (triangle); BA2 (straight line); BA3 (asterisks) BAP (square). Results were obtained from mean values of biological triplicate.

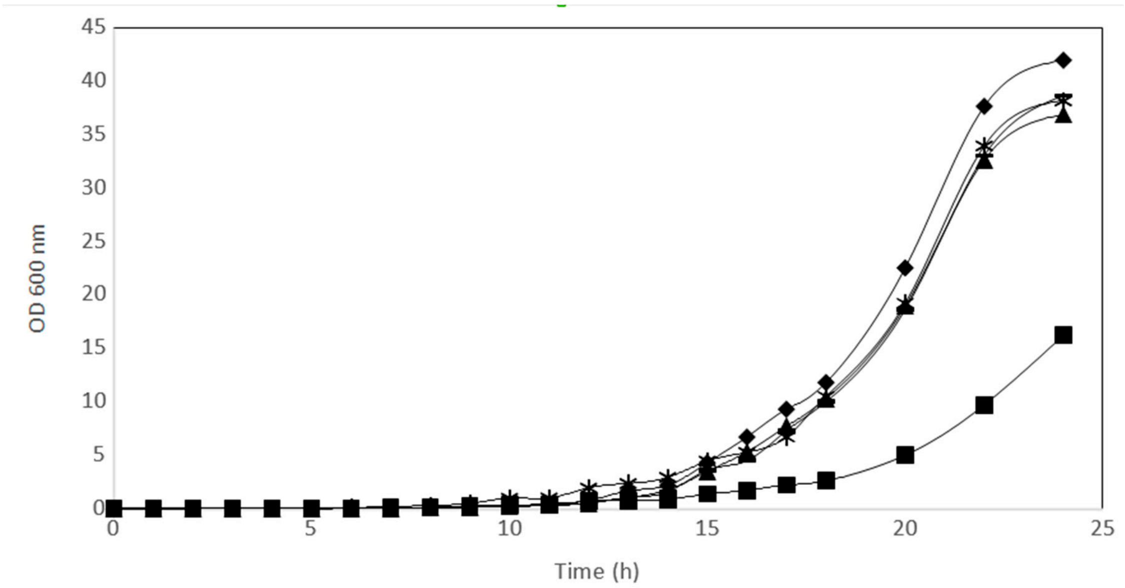

Figure S20. Relative expression of actin, xl*hasB* and *hasA* genes used in the study. The diagonally striped bar represents the transcript level of actin genes, the vertically striped bar represents the transcripts level of xl*hasB* and the gray bar represents the transcripts level of *hasA*. Experimental values obtained from biological triplicates.

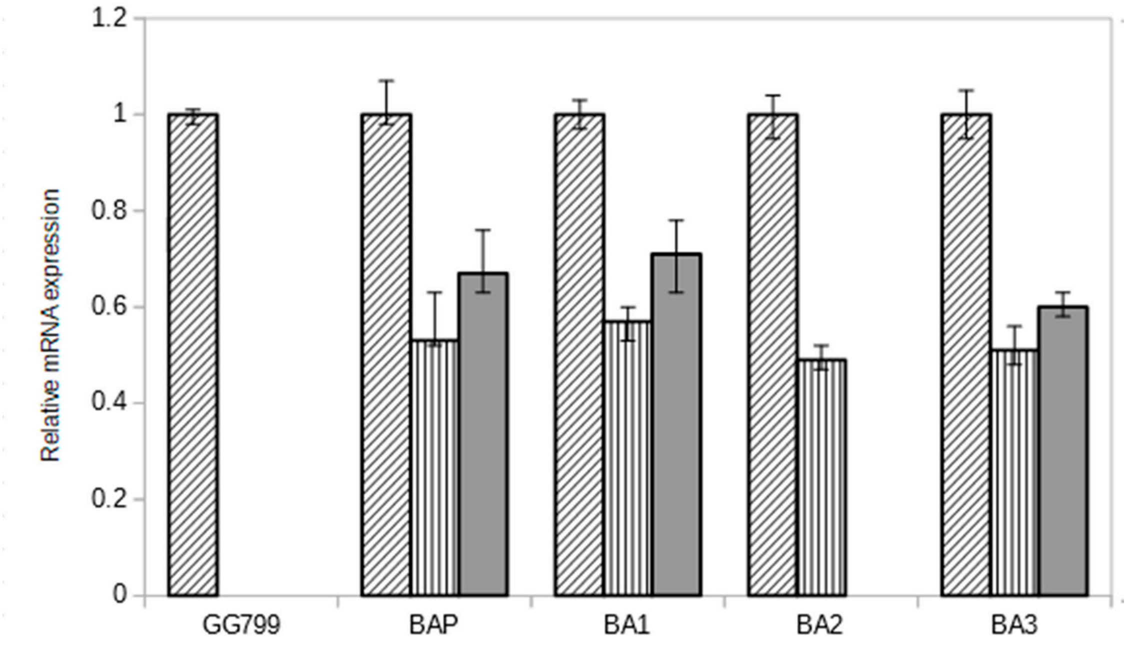

Figure S21. Gene copy number determination of pm*hasA* e xl*hasB* in *K. lactis* BAP strain. Experiment realized in Biological Triplicate.

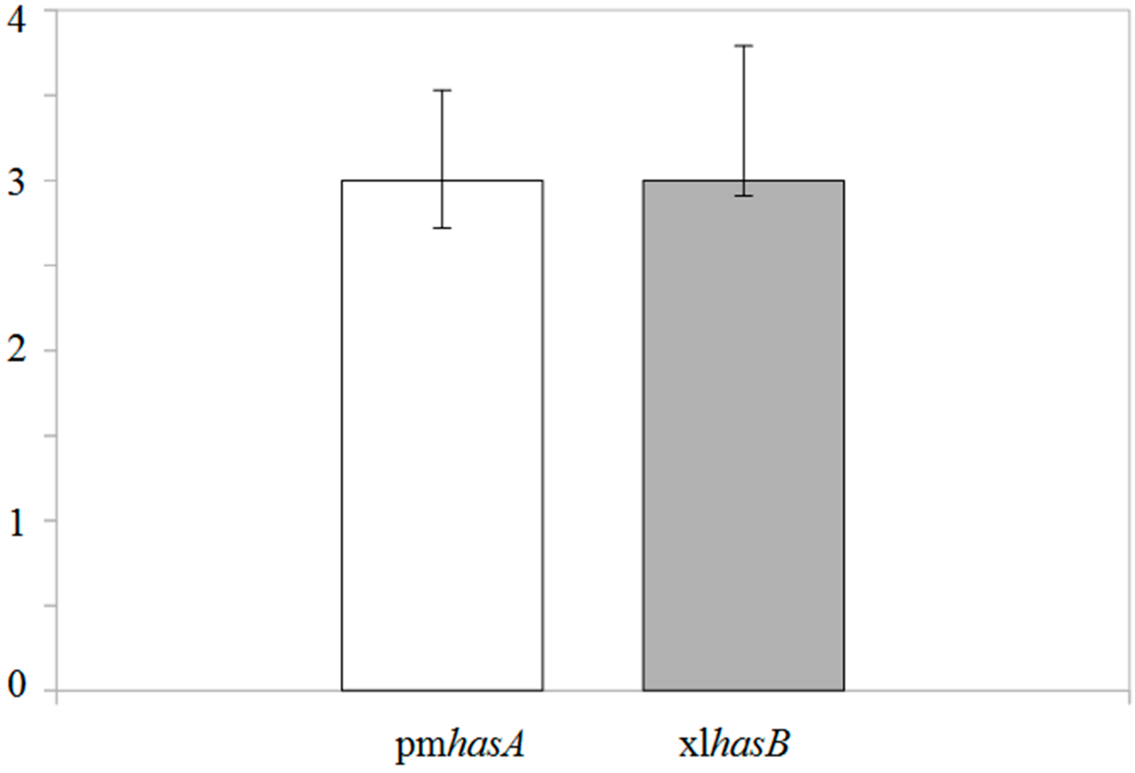

Figure S22. GPC analysis of the standard HA utilized in this study (Sigma). **Legend.** The peak on 32,75 ml represents the Hyaluronic Acid in the sample.

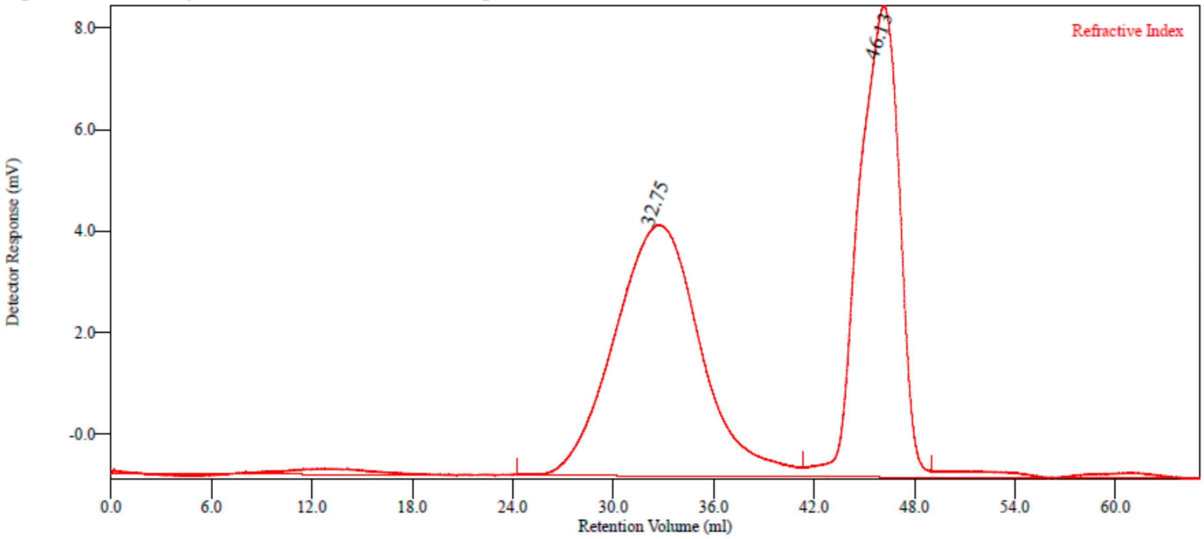

Figure S23.The standard curve for carbazole method constructed in this study and utilized for HA quantification. Experiment realized in Triplicate.

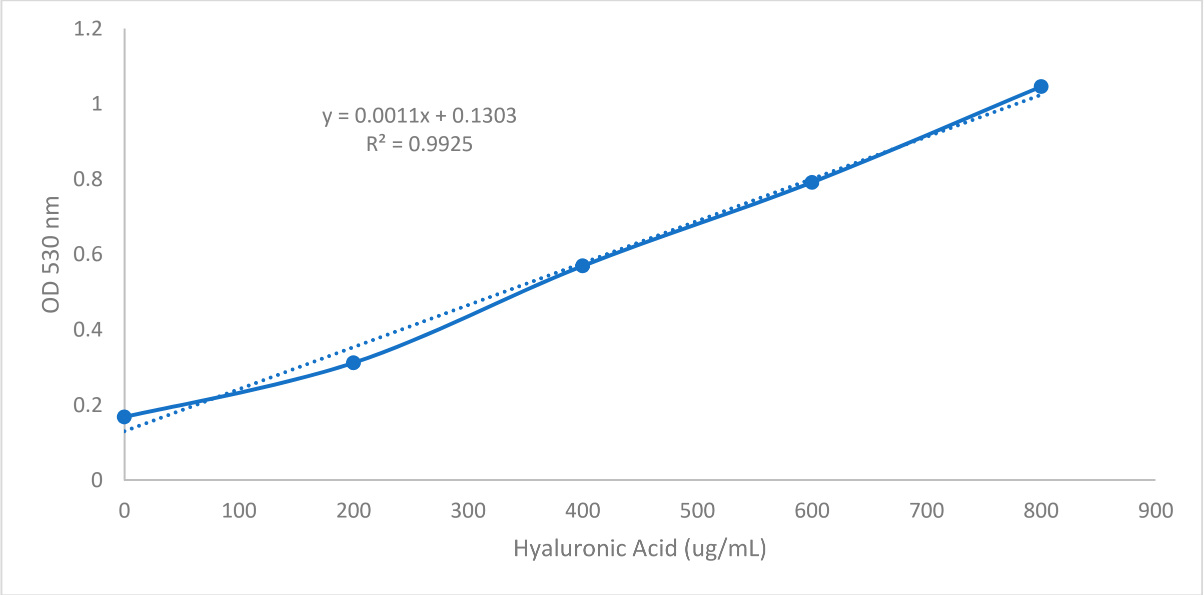

Supplement: Supplementary file 1 [file microorganisms-07-00294-s001.pdf]
